# Discovery of benzimidazole-based selective α-glucosidase inhibitors with an uncompetitive mode of action: synthesis, biological assays and computational studies

**DOI:** 10.1039/d6md00752j

**Published:** 2026-10-28

**Authors:** Francesca Mancuso, Giovanna Pitasi, Sonia Floris, Laura Ielo, Serena Vittorio, Christian Dank, Rosaria Gitto, Antonella Fais, Laura De Luca

**Affiliations:** a Department of Chemical, Biological, Pharmaceutical and Environmental Sciences, University of Messina Viale F. Stagno D'Alcontres 31 Messina I-98166 Italy framancuso@unime.it; b Department of Life and Environment Sciences, University of Cagliari Monserrato 09042 Cagliari Italy; c Institute of Applied Synthetic Chemistry TU Wien, Getreidemarkt 9 1060 Vienna Austria; d Department of Pharmaceutical Chemistry, University of Vienna Josef-Holaubek-Platz 2 A-1090 Vienna Austria; e Institute of Organic Chemistry, University of Vienna Währinger Straße 38 A-1090 Vienna Austria

## Abstract

A series of thirteen 1-(benzyloxy)-2-phenyl-1*H*-benzo[*d*]imidazole derivatives was designed, synthesized, and evaluated as α-glucosidase inhibitors. Structure–activity relationship analysis revealed that the presence of a chlorine atom at the C-6 position of the benzimidazole core, combined with suitable substituents on the pendant aromatic rings, was associated with relevant α-glucosidase inhibitory activity. Among the series, compounds 6 and 13 emerged as the most potent derivatives showing IC_50_ values of 32.35 μM and 15.70 μM, respectively, and greater potency than acarbose (IC_50_ = 139 μM) under the same experimental conditions. Importantly, the active compounds 6 and 13 did not appreciably inhibit α-amylase, thereby displaying a favorable selectivity profile over α-glucosidase. Kinetic studies highlighted an uncompetitive mechanism of inhibition for both compounds. Consistently, binding-site analysis and molecular docking suggested that they bind to a putative druggable pocket located at the entrance of the catalytic site, with chlorine substituents on the benzimidazole scaffold contributing to a more extensive network of hydrophobic interactions. Additional evaluation of antioxidant activity and cell viability reinforced the therapeutic promise of these compounds. Overall, these results identified this functionalized benzimidazole scaffold as a promising basis for the development of selective α-glucosidase inhibitors with potential application in the control of postprandial hyperglycemia.

## Abstract 2

Compound 13 emerged as a potent, selective and non-cytotoxic α-glucosidase inhibitor (IC_50_ 15.7 μM). Kinetic and computational studies clarified its uncompetitive inhibition mechanism.

## Graphical abstract

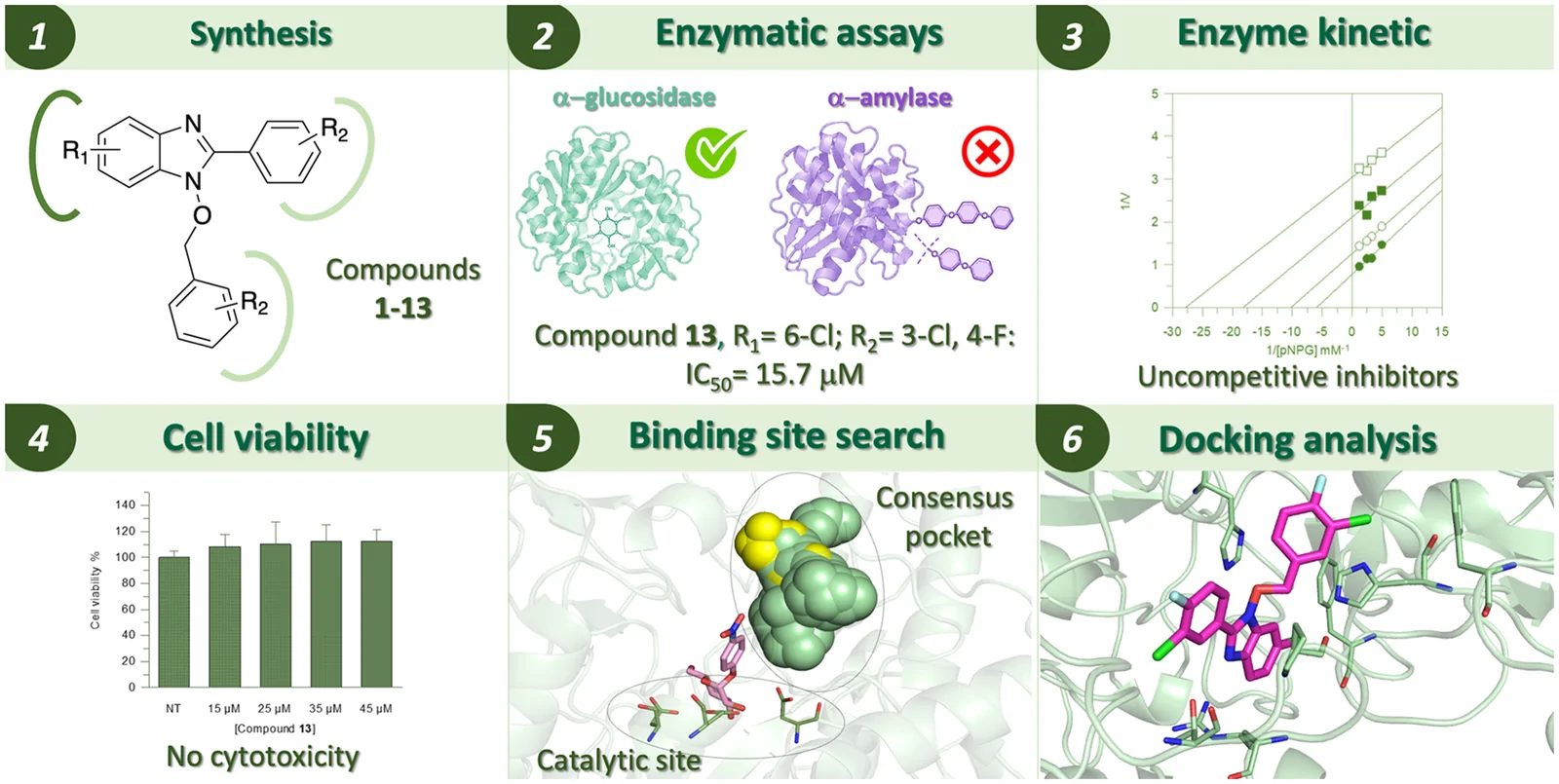

## Introduction

Diabetes mellitus (DM) is a disease characterized by high blood glucose levels that may be linked to an altered insulin production and metabolic disorder. It has been well established that DM disorder is due to insufficient insulin production by the pancreatic α-cells and/or altered cellular responses to the insulin produced by the β-cells, thus generating glucose dysregulation. DM is commonly categorized into type 1 and type 2 forms according to the underlying disease mechanisms. Type 1 diabetes develops following immune-mediated loss of the pancreatic β-cell population, with a consequent decline in insulin secretion. In contrast, type 2 diabetes is mainly characterized by reduced insulin responsiveness in peripheral tissues, which promotes sustained elevations in blood glucose even when insulin secretion is initially maintained. Both type 1 and type 2 diabetes diseases are often associated with distinct comorbidity phenomena that can cause metabolic impairments and hyperglycemia-induced complications in the liver, heart, brain and kidneys as evidenced in pathological conditions such as retinopathy, nephropathy and neuropathy as well as cardiovascular disorders; these pathological events are also associated with the high production of reactive oxygen species (ROS). The most frequently used hypoglycemic agents are generally effective in controlling blood glucose levels, but they are often related to several unwanted effects such as weight gain, cardiovascular risks, and gastrointestinal disturbances. One promising therapeutic strategy involves the inhibition of two key enzymes, α-glucosidase (EC 3.2.1.20) and α-amylase (EC 3.2.1.1), which are responsible for carbohydrate hydrolysis. Therefore, the inhibition of these enzymes decreases the rate of starch hydrolysis and consequently reduces glucose absorption and mitigates postprandial hyperglycemia occurring in type 2 diabetes. Specifically, the first step of hydrolysis is catalyzed by α-amylase, which converts polysaccharides to oligosaccharides; then, oligosaccharides are converted into glucose by α-glucosidase. Apart from α-glucosidase inhibitors (α-GIs, also known as AGIs), the current therapeutic approaches also include molecules able to inhibit dipeptidyl peptidase-IV (DPP-IV) or interact with the glucagon-like peptide-1 receptor (GLP-1R).^1–3^

α-Glucosidase is a member of the glycoside hydrolase family expressed in animals, plants, bacteria, and fungi. In the mammalian small intestine, α-glucosidase catalyzes the cleavage of α-1,4 glycosidic bonds at the non-reducing end of oligosaccharides, releasing glucose, which is absorbed into the blood stream. As a result, the inhibition of α-glucosidase induces a decrease in blood-glucose levels. Acarbose and miglitol (Fig. 1) are the most popular competitive synthetic inhibitors against α-glucosidase and/or α-amylase; however, their clinical use is restricted due to several side effects. In recent years, several classes of AGIs characterized by heterocyclic scaffolds, including benzofuran, imidazole, benzimidazole (I, Fig. 1), benzothiazole, imidazopyridine, isatin, triazole, and quinolone, have been developed.^4–7^

**Fig. 1:**
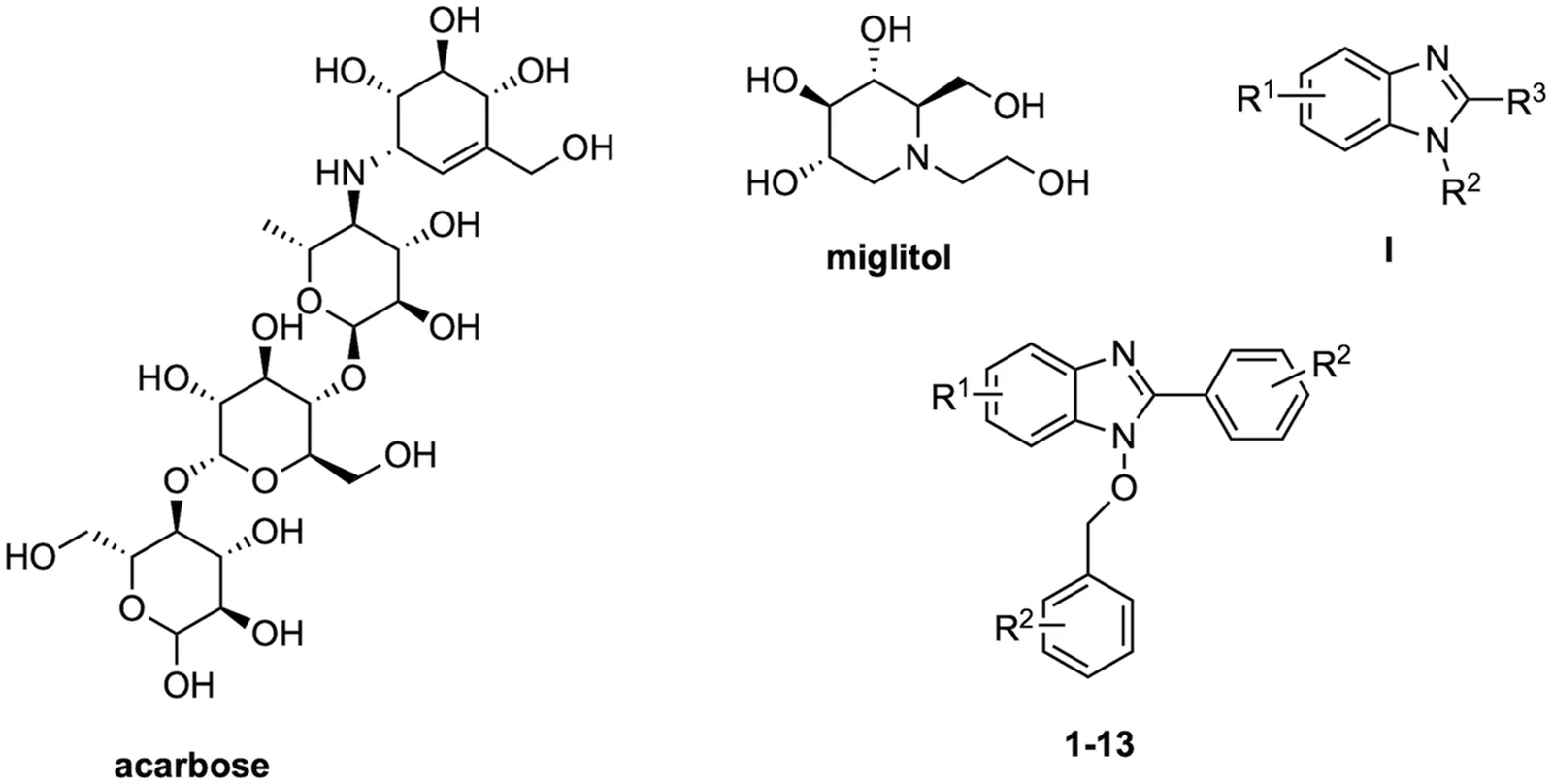
Chemical structures of acarbose and miglitol, two of the most widely used α-glucosidase inhibitors (AGIs). The general benzimidazole scaffold (I) is shown alongside the general structure of the novel designed 1-(benzyloxy)-2-phenyl-1*H*-benzo[*d*]imidazole derivatives 1–13 presented herein.

Next to acarbose and miglitol, a general benzimidazole scaffold (I) is shown next to the general structure of the herein presented 1-(benzyloxy)-2-phenyl-1*H*-benzo[*d*]imidazoles 1–13.

In this study, we report the synthesis and structural characterization of new α-glucosidase inhibitors 1–13 structurally related to previously reported benzimidazole-based compounds (I, Fig. 1)^8–12^ possessing both the core scaffold and appropriate moieties eliciting profitable interactions within key residues of catalytic cavities.^12–15^ Since oxidative stress plays a crucial role in the pathogenesis of diabetes and its complications, the identification of compounds endowed with both α-glucosidase inhibitory and antioxidant activities represents an attractive therapeutic strategy. Indeed, chronic hyperglycemia is known to promote the generation of ROS and to impair cellular antioxidant defense systems, thereby exacerbating oxidative damage. Therefore, for the most representative compounds, we first assessed cell viability in Caco-2 cells and subsequently evaluated their antioxidant effects in the same cell line. Finally, computational studies were performed to analyze the network of plausible interactions within the enzyme binding site for this series of benzimidazoles active as α-glucosidase inhibitors.

## Experimental section

### Materials and methods

#### α-Glucosidase inhibition assay

The inhibitory activity of the compounds against α-glucosidase was determined according to a previously reported method.^16^*Saccharomyces cerevisiae* α-glucosidase (0.125 U mL^−1^) was combined with each test compound at different concentrations in 0.1 M sodium phosphate buffer at pH 6.8. After incubation for 15 min at 37 °C, the reaction was initiated by adding *p*-nitrophenyl α-d-glucopyranoside (pNPG, 5 mM). The mixture was incubated for a further 15 min at the same temperature and the reaction was then terminated with sodium carbonate solution (0.2 M). Enzyme activity was quantified by monitoring the release of *p*-nitrophenol at 405 nm using a VANTAstar microplate reader (BMG LABTECH GmbH, Germany). DMSO was used as a vehicle control where necessary, and its final concentration was kept below 8% v/v, a level that did not interfere with enzymatic activity. The inhibition mechanism was investigated by carrying out assays at different inhibitor concentrations and analysing the data through Lineweaver–Burk double-reciprocal plots.

#### α-Amylase inhibition assay

The effects of compounds 6 and 13 on α-amylase activity were evaluated using 2-chloro-4-nitrophenyl-α-d-maltotrioside (CNPG3) as a chromogenic substrate. Porcine pancreatic α-amylase (40 μL, 1 mg mL^−1^) was mixed in a microplate well with sodium phosphate buffer (60 μL, 50 mM) at pH 7.0 and sodium chloride solution (20 μL, 1 M), with or without the test compounds. Following incubation at 37 °C for 10 minutes, the enzymatic reaction was started by adding CNPG3 solution (80 μL, 2.5 mM). Substrate hydrolysis was monitored at 405 nm by measuring the release of 2-chloro-nitrophenol with a VANTAstar microplate reader (BMG LABTECH GmbH, Germany).

#### Cell viability and intracellular ROS levels

Caco-2 cells obtained from American Type Culture Collection (ATCC, Manassas, VA, USA) were assessed for viability using the colorimetric 3-(4,5-dimethylthiazol-2-yl)-2,5-diphenyltetrazolium bromide (MTT) assay, as previously described.^12^ Metabolically active cells reduce MTT to formazan *via* dehydrogenase activity. The intracellular purple formazan crystals are subsequently solubilized and quantified using spectrophotometry (VANTAstar BMG LABTECH GmbH, Ortenberg, Germany). Caco-2 cells were treated with the same compound concentrations to measure cellular ROS levels using the 2′,7′-dichlorofluorescein diacetate (DCFH-DA) method. After 24 h, the cells were incubated with DCFH-DA (10 μM) at 37 °C for 30 min and then treated with 1 mM H_2_O_2_. The fluorescence intensity of DCF was immediately measured with a fluorescence plate reader at an excitation wavelength of 485 nm and an emission wavelength of 530 nm (VANTAstar BMG LABTECH GmbH, Ortenberg, Germany).

#### α-Glucosidase protein model preparation

The 3D structural model of α-glucosidase from *Saccharomyces cerevisiae* was predicted by AlphaFold3 through the AlphaFold server (https://alphafoldserver.com/, accessed on July 2026).^17^ The primary sequence was retrieved from the UniProt database (ID P53341) and used as input for structure prediction.^18^ The resulting model exhibited high global confidence, with a predicted TM-score (pTM) of 0.96, suggesting a highly reliable global fold. Furthermore, no disordered regions or steric clashes were detected, as indicated by a *fraction_disordered* value of 0.0 and a *has_clash* value of 0.00. The quality of the predicted structure was further assessed using the SAVES v6.0 validation server (https://saves.mbi.ucla.edu/, accessed on July 2026), incorporating PROCHECK, ERRAT, and Verify3D analyses. ERRAT yielded an overall quality factor of 94.72, indicating favourable non-bonded atomic interactions. Verify3D analysis showed that 98.63% of residues achieved an average 3D–1D score of ≥0.1, exceeding the acceptance threshold of 80%. In addition, Ramachandran plot analysis performed with PROCHECK revealed that 90.1% of residues were in the most favoured regions, 9.1% in additionally allowed regions, and only 0.4% in disallowed regions.

The Ramachandran plot is provided in the SI (Fig. S33).

The predicted structure was also evaluated using the SWISS-MODEL Structure Assessment tool (https://swissmodel.expasy.org/assess, accessed on July 2026).^19^ The model achieved a QMEANDisCo global score of 0.87 and a normalized QMEAN4 score of approximately 0.80, both indicative of strong agreement with experimentally determined protein structures. Collectively, these validation metrics confirmed the high quality and reliability of the predicted α-glucosidase model, supporting its use in subsequent computational studies. The so obtained structure was prepared by means of the Protein Preparation Workflow implemented in the Schrödinger Suite V. 2026-2.^20^

#### Binding site mapping and molecular docking

Considering the uncompetitive inhibition mechanism exerted by the tested compounds, molecular docking was performed on the enzyme-substrate complex generated as follows. The pNPG, which is routinely employed as a substrate in α-glucosidase inhibition assays, was docked into the catalytic pocket by means of GOLD V. 2026.1.1 using a scaffold constrain approach.^21^ This strategy was adopted to obtain docking solutions in which the glucopyranosyl moiety assumes a pose conducive to the reaction consistent with the crystallographic poses observed in the X-ray structures of yeast isomaltase complexed with substrate analogues (PDB ID 3AXI and 3AXH). The side chains of residues E276, D349 and D68 were set as flexible and ten poses were generated. ChemPLP was used as scoring function. The “*allow early termination*” option was deactivated. The top-ranked docking pose was selected. The resulting enzyme-substrate complex was optimized by energy minimization using the Prime module of Schrödinger Suite keeping the backbone fixed.^22^ The OLPS4 force field and VSGB solvation model were used for the calculation. The so optimized complex underwent binding site mapping to identify druggable pockets distinct from the catalytic site. The analysis was performed by means of SiteMap^23^ and FTMap^24^ (https://ftmap.bu.edu/, accessed on July 2026), using the default settings. Ligand structures were optimized by the AMMP tool implemented in VEGA ZZ suite using the CHARMM force field,^25^ while flexible torsions were explored using the Boltzmann jump method as implemented in VEGA ZZ. Docking of the tested compounds was performed on the enzyme–substrate complex within a consensus site identified by both SiteMap and FTMap methods, using the same protocol applied for the substrate. However, in this case, all protein residues were kept rigid throughout the calculation. The best docking pose was chosen for analysis and representation.

### Chemistry

Chemicals and solvents were sourced from Thermo Fisher Scientific-Alfa Aesar (Segrate, Italy) and Merck Sigma-Aldrich (Milan, Italy) and employed as received. Reaction progress and compound purity were monitored by analytical thin-layer chromatography (TLC) using glass-supported silica gel plates (Merck, 60F254). Nuclear magnetic resonance analyses, including ^1^H and ^13^C NMR, were carried out on a Varian Gemini 500 instrument (Palo Alto, CA, USA) with chloroform-*d* as the deuterated solvent. Resonance signals are reported as chemical shifts (*δ*) in parts per million (ppm), while scalar coupling constants (*J*) are expressed in hertz (Hz) for compounds 1–13. Additionally, a two-dimensional ^1^H–^13^C heteronuclear single quantum coherence (HSQC) NMR experiment was performed for compound 10 thus supporting the assignment of directly bonded proton and carbon resonances (Fig. S33, SI). Infrared characterization was performed by ATR-FTIR spectroscopy using a Shimadzu spectrophotometer. Melting temperatures were measured with Büchi B-545 apparatus (BÜCHI Labortechnik AG, Flawil, Switzerland) and are reported without correction. High-resolution mass spectrometric data were acquired on an Orbitrap Exploris mass spectrometer (MB11205C) employing an ACN/MeOH solvent mixture containing 1% H_2_O. Elemental composition (C, H, N) was determined using a Carlo Erba 1106 Analyzer, confirming that the final compounds (1–13) possessed a purity of at least 95%, with experimental values deviating by no more than ±0.4% from theoretical calculations.

#### General procedure for the synthesis of 1-(benzyloxy)-2-phenyl-1*H*-benzo[*d*]imidazoles 1–13

An appropriately substituted 2-nitroaniline (1.0 equiv., 250 mg) was dissolved in dry THF (≈7 mL mmol^−1^) and cooled in an ice bath. Sodium hydride (60% dispersion in mineral oil, 3.0 equiv.) was slowly added, and the resulting suspension was stirred for 10 min. The appropriate benzyl bromide (1.0 equiv.) was then added dropwise, and the reaction was heated at reflux for 2 h. Following cooling to room temperature, a second portion of sodium hydride (3.0 equiv.) and benzyl bromide (1.0 equiv.) was added in an ice bath. The mixture was heated at reflux for 2–4 h and monitored by TLC (*n*-hexane/ethyl acetate, 9 : 1) until the disappearance of the reaction intermediates. Upon completion, the reaction mixture was evaporated under vacuum, and the residue was extracted with dichloromethane/water (3 × 30 mL). The combined organic layers were washed with saturated aqueous NaCl solution, dried over anhydrous Na_2_SO_4_, filtered, and then concentrated under reduced pressure. The crude solid was triturated with *n*-hexane to afford the desired product in solid form. The full characterization data of all synthesized 1-(benzyloxy)-2-phenyl-1*H*-benzo[*d*]imidazoles derivatives 1–13 are given below.

##### 1-(Benzyloxy)-2-phenyl-1*H*-benzo[*d*]imidazole (1)


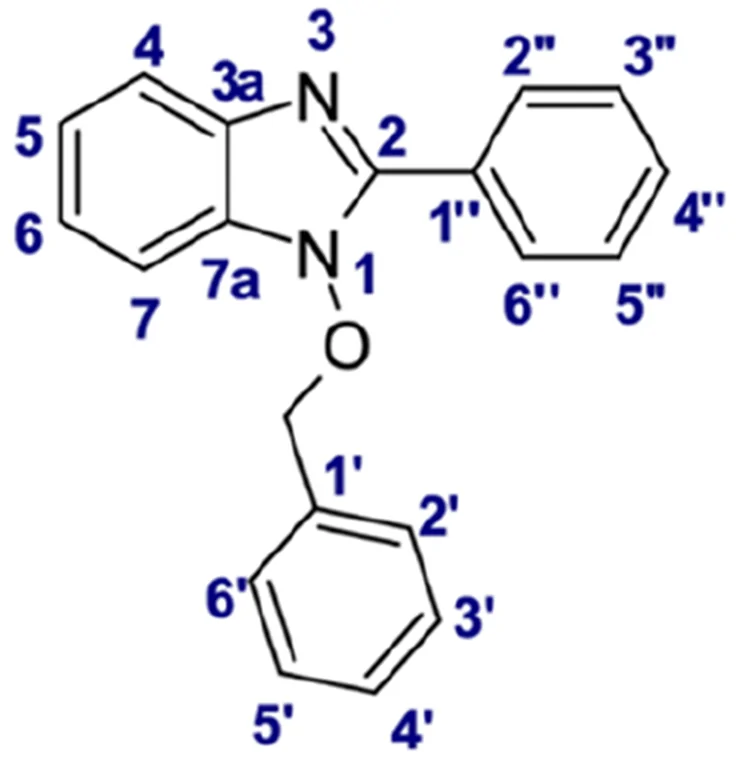
According to the general procedure by employing 2-nitroaniline (CAS no. 88-74-4) and benzyl bromide (CAS no. 100-39-0) to afford the title compound (CAS no. 168209-86-7, 1) as on off-white powder. Yield: 42% (228 mg, 0.76 mmol), mp 133–135 °C. ^1^H NMR (500 MHz, CDCl_3_): *δ* 5.04 (s, 2H, –CH_2_–), 7.22–7.24 (m, 2H, H-2′ and H-6′), 7.28–7.31 (m, 3H, H-3′, H-4′ and H-5′), 7.34–7.35 (m, 2H, H-5 and H-6), 7.44–7.46 (m, 1H, H-7), 7.49–7.50 (m, 3H, H-3″, H-4″ and H-5″), 7.78–7.80 (m, 1H, H-4), 8.16–8.18 (m, 2H, H-2″ and H-6″). ^13^C NMR (126 MHz, CDCl_3_): *δ* 80.16 (–CH_2_–), 108.85, 120.32, 122.84, 123.39, 128.48, 128.61, 128.62, 128.67, 129.56, 129.90, 130.13, 131.39, 132.85, 138.51, 147.62 (C=N). IR (ATR,_max_, cm^−1^): 3055, 3031, 2892, 1558, 1469, 1452, 1441, 1385, 1319, 1273, 1241, 1064, 1029, 950, 906, 833, 758, 729, 689, 673, 638, 612, 569, 534, 486, 440. HR-MS (ESI^+^): *m*/*z* calcd for C_20_H_16_N_2_O [M + H]^+^ 301.1335; found 301.1334. Elemental analysis calcd. for C_20_H_16_N_2_O: C, 79.98; H, 5.37; N, 9.33; found: C, 79.96; H, 5.39; N, 9.30.

##### 1-(Benzyloxy)-5-fluoro-2-phenyl-1*H*-benzo[*d*]imidazole (2)


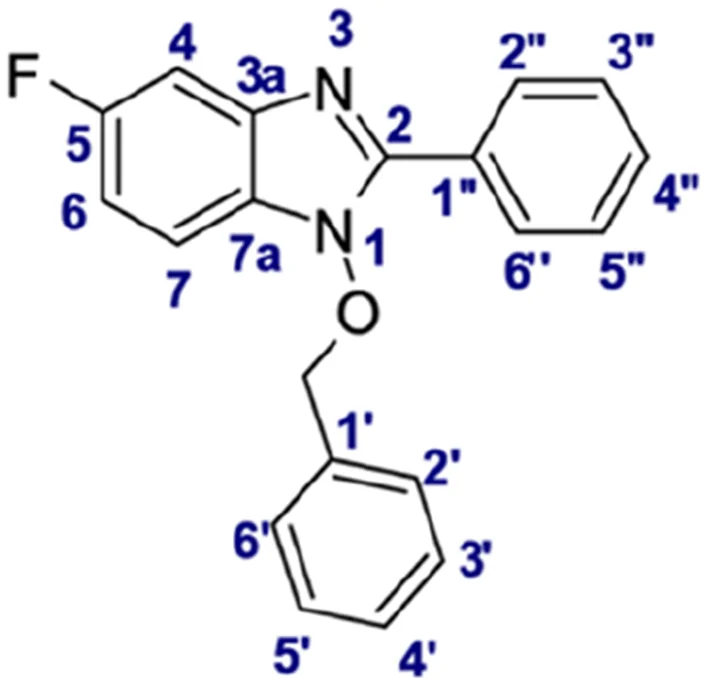
According to the general procedure by employing 4-fluoro-2-nitroaniline (CAS no. 364-78-3) and benzyl bromide (CAS no. 100-39-0) to afford the title compound 2 as pale yellow powder. Yield: 83% (422 mg, 1.33 mmol), mp 269–270 °C. ^1^H NMR (500 MHz, CDCl_3_): *δ* 5.01 (s, 2H, –CH_2_–), 7.03–7.04 (m, 1H, H-6), 7.06–7.07 (m, 1H, H-7), 7.20–7.21 (m, 2H, H-2′ and H-6′), 7.28–7.31 (m, 2H, H-3′ and H-5′), 7.34–7.35 (m, 1H, H-4′), 7.49–7.50 (m, 3H, H-3″, H-4″ and H-5″), 7.68–7.71 (m, 1H, H-4), 8.13–8.15 (m, 2H, H-2″ and H-6″). ^13^C NMR (126 MHz, CDCl_3_): *δ* 80.29 (–CH_2_–), 95.60 (d, ^2^*J*_C–F_ = 25.8 Hz), 111.32 (d, ^2^*J*_C–F_ = 25.8 Hz), 121.23 (d, ^3^*J*_C–F_ = 9.5 Hz), 128.31, 128.41, 128.70, 129.96, 131.56, 131.67, 132.61, 134.80 (d, ^4^*J*_C–F_ = 2.9 Hz), 148.42 (C=N), 159.99 (^1^*J*_C–F_ = 241.3 Hz), 181.59. IR (ATR,_max_, cm^−1^): 3031, 1739, 1630, 1560, 1491, 1469, 1455, 1442, 1383, 1360, 1304, 1267, 1240, 1217, 1161, 1115, 1051, 1028, 963, 945, 904, 848, 824, 805, 767, 754, 722, 689, 672, 638, 603. HR-MS (ESI^+^): *m*/*z* calcd for C_20_H_15_FN_2_O [M + H]^+^ 319.1246; found: 319.1238. Elemental analysis calcd. for C_20_H_15_FN_2_O: 75.46, H, 4.75, N, 8.80; found: C, 75.43, H, 4.78, N, 8.82.

##### 1-(Benzyloxy)-6-fluoro-2-phenyl-1*H*-benzo[*d*]imidazole (3)


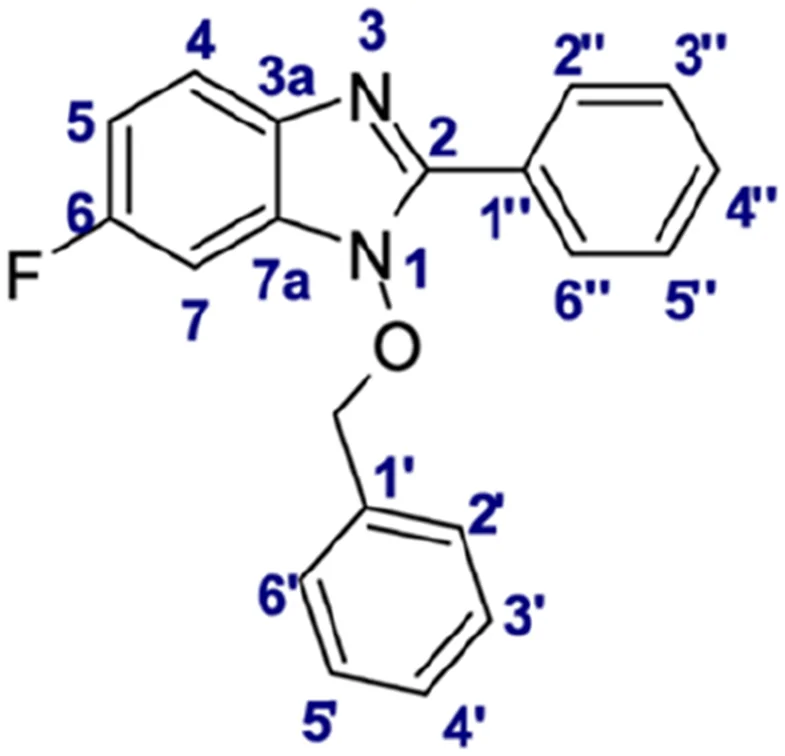
According to the general procedure by employing 5-fluoro-2-nitroaniline (CAS no. 2369-11-1) and benzyl bromide (CAS no. 100-39-0) to afford the title compound 3 as pale yellow powder. Yield: 39% (199 mg, 0.62 mmol), mp 158–160 °C. ^1^H NMR (500 MHz, CDCl_3_): 5.02 (s, 2H, –CH_2_–),7.03 (dd, ^3^*J* = 9.3 Hz, ^4^*J* = 2.5 Hz, 1H, H-5), 7.19–7.20 (m, 2H, H-2′ and H-6′), 7.27–7.31(m, 3H, H-4, H-3′ and H5′), 7.33–7.35 (m, 1H, H-4′), 7.45 (dd, ^3^*J* = 9.3 Hz, ^4^*J* = 2.5 Hz, 1H, H-7), 7.49–7.51 (m, 3H, H-3″, H-4″ and H-5″), 8.13–8.15 (m, 2H, H-2″ and H-6″). ^13^C NMR (126 MHz, CDCl_3_): *δ* 80.36, (–CH_2_–), 106.00 (d, ^2^*J*_C–F_ = 25.8 Hz), 109.32 (d, ^3^*J*_C–F_ = 10.5 Hz), 111.81 (d, ^2^*J*_C–F_ = 25.8 Hz), 128.04, 128.33, 128.43, 128.69 (d, ^4^*J*_C–F_ = 1.9 Hz), 129.67, 129.93, 130.35, 132.66, 138.88 (d, ^3^*J*_C–F_ = 13.4 Hz), 148.88 (C=N), 159.68 (d, ^1^*J* = 238.4 Hz). IR (ATR,_max_, cm^−1^): 3032, 1739, 1626, 1591, 1482, 1451, 1431, 1370, 1319, 1263, 1217, 1173, 1138, 1109, 1065, 1031, 950, 909, 886, 847, 791, 768, 755, 749, 716, 688, 679, 646, 609. HR-MS (ESI^+^): *m*/*z* calcd for C_20_H_15_FN_2_O [M + H]^+^ 319.1246; found: 319.1237. Elemental analysis calcd. for C_20_H_15_FN_2_O: C, 75.46, H, 4.75, N, 8.80; found: C, 75.42, H, 4.78, N, 8.81.

##### 1-(Benzyloxy)-5-chloro-2-phenyl-1*H*-benzo[*d*]imidazole (4)


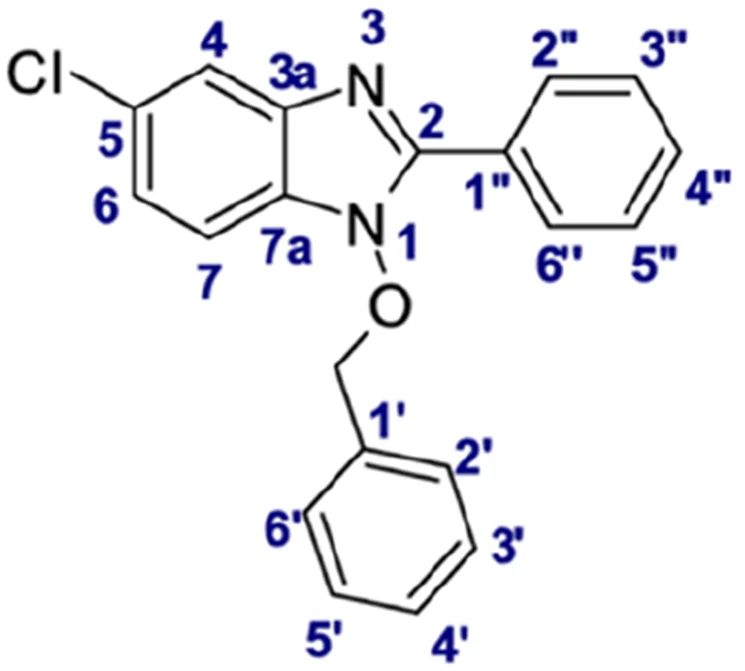
According to the general procedure by employing 4-chloro-2-nitroaniline (CAS no. 89-63-4) and benzyl bromide (CAS no. 100-39-0) to afford the title compound 4 as pale yellow powder. Yield: 48% (231 mg, 0.69 mmol), mp 135–136 °C. ^1^H NMR (500 MHz, CDCl_3_): *δ* 5.01 (s, 2H, –CH_2_–), 7.20–7.21 (d, ^3^*J* = 7.3 Hz, 2H, H-2′ and H-6′), 7.24 (d, ^3^*J* = 8.8 Hz, 1H, H-6), 7.30 (t, ^3^*J* = 6.9 Hz, 2H, H-3′ and H-5′), 7.35 (d, ^3^*J* = 6.9 Hz,1H, H-4′), 7. 37 (br s, 1H, H-4), 7.50–7.51 (m, 3H, H-3″, H-4″ and H-5″), 7.67 (d, ^3^*J* = 8.8 Hz, 1H, H-7), 8.13–8.15 (m, 2H, H-2″ and H-6″). ^13^C NMR (126 MHz, CDCl_3_): *δ* 80.40 (–CH_2_–), 108.93, 121.19, 123.58, 128.22, 128.43, 128.70, 128.70, 129.17, 129.74, 129.98, 130.40, 132.05, 132.52, 137.05, 148.44 (C=N). IR (ATR,_max_, cm^−1^): 3064, 3031, 2948, 2888, 1616, 1462, 1441, 1382, 1327, 1269, 1078, 932, 904, 836, 811, 767, 745, 696, 688, 679, 633, 590, 546, 488, 433. HR-MS (ESI^+^): *m*/*z* calcd for C_20_H_15_ClN_2_O [M + H]^+^ 335.0906; found 335.0945. Elemental analysis calcd. for C_20_H_15_ClN_2_O: C, 71.75; H, 4.52; N, 8.37; found: C, 71.73; H, 4.54; N, 8.39.

##### 1-(Benzyloxy)-6-chloro-2-phenyl-1*H*-benzo[*d*]imidazole (5)


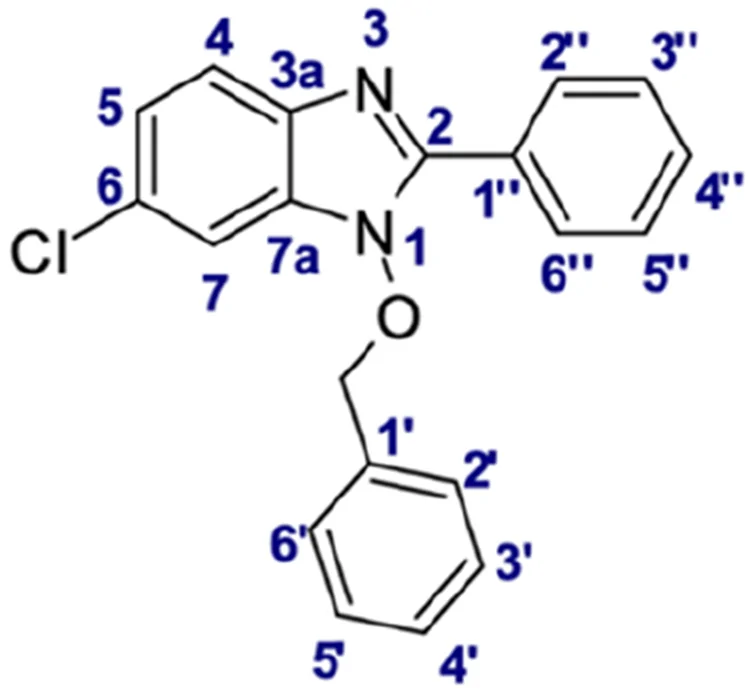
According to the general procedure by employing 5-chloro-2-nitroaniline (CAS no. 1635-61-6) and benzyl bromide (CAS no. 100-39-0) to afford the title compound (CAS no. 168209-91-4, 5) as off-white powder. Yield: 72% (347 mg, 1.03 mmol), mp 133–135 °C. ^1^H NMR (500 MHz, CDCl_3_): *δ* 5.01 (s, 2H, –CH_2_–), 7.17–7.18 (m, 2H, H-2′ and H-6′), 7.23–7.25 (dd, ^3^*J* = 8.3 Hz, ^4^*J* = 1.5 Hz, 1H, H-5), 7.28 (m, 2H, H-3′ and H-5′), 7.29 (d, ^3^*J* = 8.3 Hz, H-4), 7.32–7.34 (m, 1H, H-4′), 7.49–7.50 (m, 3H, H-3″, H-4″ and H-5″), 7.74 (d, ^4^*J* = 1.5 Hz, H-7), 8.12–8.14 (m, 2H, H-2″ and H-6″). ^13^C NMR (126 MHz, CDCl_3_): 80.42 (–CH_2_–), 109.70, 117.74, 119.99, 123.82, 127.67, 128.16, 128.40, 128.50, 128.70, 128.71, 129.72, 129.95, 130.12, 130.46, 132.56, 139.19, 148.76 (C=N). IR (ATR,_max_, cm^−1^): 3032, 1588, 1459, 1422, 1383, 1359, 1306, 1244, 1217, 1168, 1127, 1056, 1032, 970, 949, 920, 906, 886, 843, 790, 765, 744, 698, 688, 681, 671, 642. HR-MS (ESI^+^): *m*/*z* calcd for C_20_H_15_ClN_2_O [M + H]^+^ 335.0951; found 335.0946. Elemental analysis calcd. for C_20_H_15_ClN_2_O: C 71.75, H 4.52, N, 8.37; found: C 71.73, H 4.54, N 8.40.

##### 6-Chloro-1-((3-chlorobenzyl)oxy)-2-(3-chlorophenyl)-1*H*-benzo[*d*]imidazole (6)


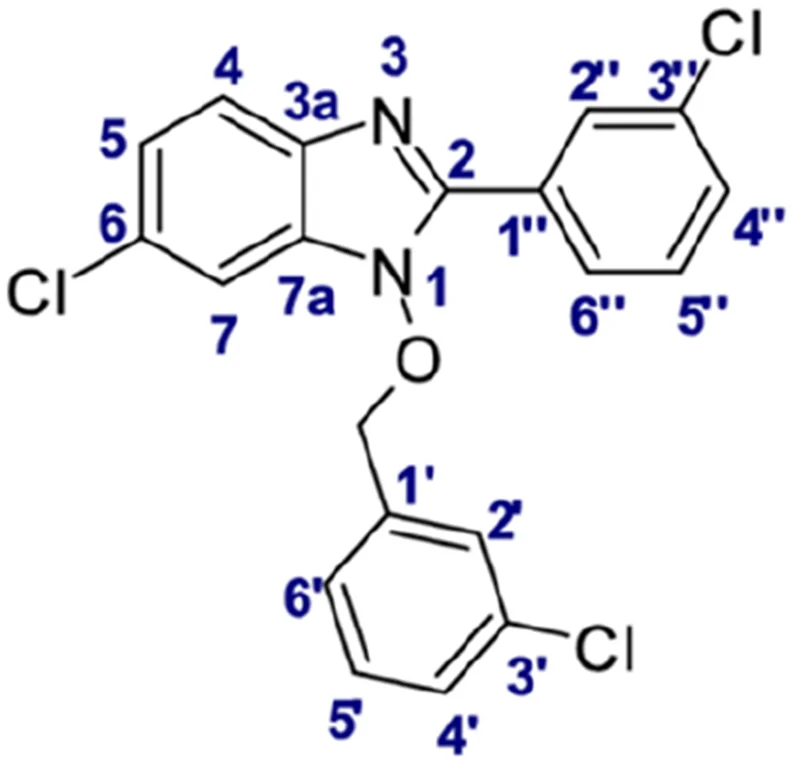
According to the general procedure by employing 5-chloro-2-nitroaniline (CAS no. 1635-61-6) and 3-chlorobenzyl bromide (CAS no. 766-80-3) to afford the title compound 6 as light pink powder. Yield: 56% (273 mg, 0.68 mmol), mp 133–134 °C. ^1^H NMR (500 MHz, CDCl_3_): *δ* 5.01 (s, 2H, –CH_2_–), 6.92–6.94 (m, 1H, H-5″), 7.14–7.15 (m, 1H, H-5), 7.18–7.19 (dd, ^3^*J* = 7.8 Hz, ^4^*J* = 2.9 Hz, 1H, H-6′), 7.30–7.31 (d, ^4^*J* = 2.9 Hz, 1H, H-2′), 7.31–7.32 (d, ^4^*J* = 1.5 Hz, 1H, H-4), 7.35–7.37 (dd, ^3^*J* = 7.8 Hz, ^4^*J* = 2.9 Hz, 1H, H-4′), 7.40–7.42 (dd, ^3^*J* = 7.8 Hz, ^4^*J* = 2.9, 1H, H-5′), 7.45–7.46 (m, 1H, H-4″), 7.76 (d, ^4^*J* = 1.5 Hz, 1H, H-7), 7.91–7.93 (m, 1H, H-6″). 8.01–8.02 (m, 1H, H-2″). ^13^C NMR (126 MHz, CDCl_3_): *δ* 79.55 (–CH_2_–), 109.67, 120.36, 124.37, 126.52, 128.04, 128.40, 128.83, 129.62, 129.67, 129.89, 129.97, 129.99, 130.19, 130.49, 134.01, 134.69, 134.72, 139.18, 147.46 (C=N). IR (ATR,_max_, cm^−1^): 3022, 2919, 1739, 1593, 1481, 1456, 1403, 1376, 1307, 1280, 1250, 1216, 1160, 1125, 1092, 1068, 1053, 942, 920, 910, 896, 874, 843, 787, 762, 749, 738, 696, 671, 645, 599. HR-MS (ESI^+^): *m*/*z* calcd for C_20_H_13_Cl_3_N_2_O [M + H]^+^ 403.0171; found: 403.0169. Elemental analysis calcd. for C_20_H_13_Cl_3_N_2_O: C, 59.51, H, 3.25, N, 6.94; found: C, 59.49, H, 3.29, N, 6.91;

##### 6-Chloro-1-((3-methylbenzyl)oxy)-2-(*m*-tolyl)-1*H*-benzo[*d*]imidazole (7)


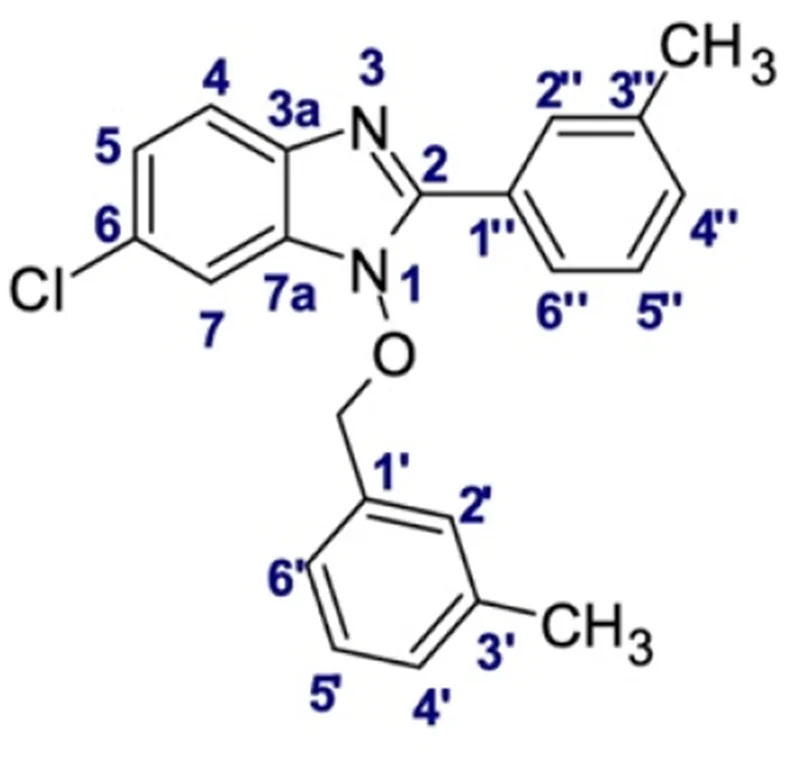
According to the general procedure by employing 5-chloro-2-nitroaniline (CAS no. 1635-61-6) and 3-methylbenzyl bromide (CAS no. 620-13-3) to afford the title compound 7 as white powder. Yield: 28% (146 mg, 0.40 mmol), mp 74–76 °C. ^1^H NMR (500 MHz, CDCl_3_): *δ* 2.27 (s, 3H, –CH_3_-3′), 2.41 (s, 3H, –CH_3_-3″), 4.97 (s, 2H, –CH_2_–), 6.96 (m, 1H, H-4′), 7.0 (br s, 1H, H-2′), 7.14–7.19 (m, 2H, H-6′ and H-4″), 7.25 (dd, ^3^*J* = 8.3 Hz, ^4^*J* = 1.47 Hz, 1H, H-5), 7.30–7.33 (m, 2H, H-4 and H-5′), 7.37–7.40 (m, 1H, H-5″), 7.74 (d, ^4^*J* = 1.5 Hz, H-7), 7.94–7.95 (m, 2H, H-2″ and H-6″). ^13^C NMR (126 MHz, CDCl_3_): *δ* 21.20 (–CH_3_), 21.37, (–CH_3_), 80.48 (–CH_2_–), 109.67, 119.95, 123.69, 125.59, 127.01, 128.04, 128.31, 128.52, 128.58, 129.12, 130.13, 130.38, 130.76, 131.23, 132.49, 138.38, 138.47, 139.22, 148.95 (C=N). IR (ATR,_max_, cm^−1^): 3022, 2919, 1739, 1593, 1481, 1456, 1403, 1376, 1307, 1280, 1250, 1216, 1160, 1125, 1092, 1068, 1053, 942, 920, 910, 896, 874, 843, 787, 762, 749, 738, 696, 671, 645, 599. HR-MS (ESI^+^): *m*/*z* calcd for C_22_H_19_ClN_2_O [M + H]^+^ 363.1264; found: 363.1255. Elemental analysis calcd. for C_22_H_19_ClN_2_O: C, 72.82, H, 5.28, N, 7.72; found: C, 72.78, H, 5.30, N, 7.75.

##### 6-Chloro-1-((4-fluorobenzyl)oxy)-2-(4-fluorophenyl)-1*H*-benzo[*d*]imidazole (8)


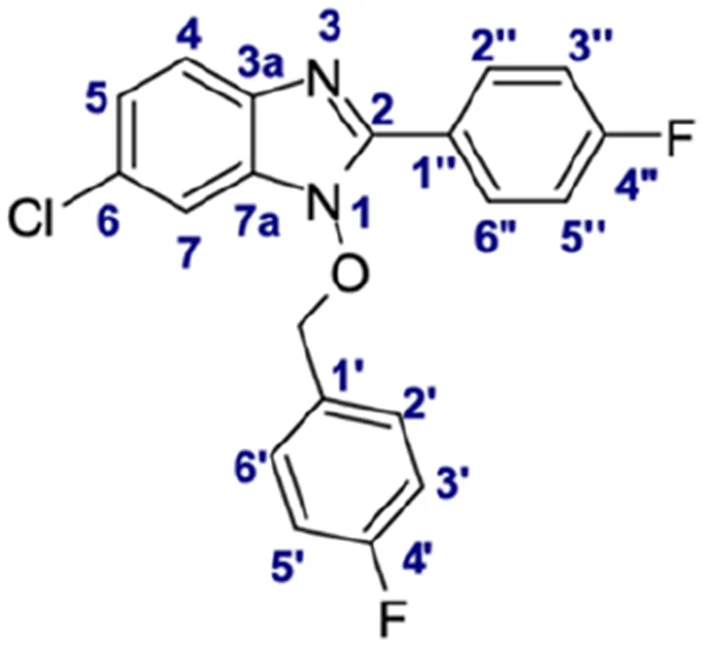
According to the general procedure by employing 5-chloro-2-nitroaniline (CAS no. 1635-61-6) and 4-fluorobenzyl bromide (CAS no. 459-46-1) to afford the title compound 8 as light pink powder. Yield: 72% (384 mg, 1.03 mmol), mp 104–106 °C. ^1^H NMR (500 MHz, CDCl_3_): *δ* 4.97 (s, 2H, –CH_2_–), 6.90 (t, ^3^*J* = 8.3 Hz, 2H, H-3′ and H-5′), 7.03–7.04 (m, 2H, H-3″ and H-5″), 7.15 (t, ^3^*J* = 8.3 Hz, 2H, H-2′ and H-6′), 7.24 (d, ^3^*J* = 8.8 Hz, H-4), 7.28 (d, ^3^*J* = 8.8 Hz, ^4^*J* = 1.5 Hz, H-5), 7.72 (d, ^4^*J* = 1.5 Hz, 1H, H-7), 8.01–8.03 (m, 2H, H-2″ and H-6″). ^13^C NMR (126 MHz, CDCl_3_): *δ* 79.33 (–CH_2_–), 109.64, 115.70 (d, ^2^*J*_C–F_ = 21.9 Hz), 115.76 (d, ^2^*J*_C–F_ = 21.9 Hz), 120.10, 123.94, 124.39 (d, ^4^*J*_C–F_ = 3.8 Hz), 128.32 (d, ^4^*J*_C–F_ = 3.8 Hz), 128.56, 129.7, 130.63 (d, ^3^*J*_C–F_ = 8.6 Hz), 132.06 (d, ^3^*J*_C–F_ = 8.6 Hz), 139.19, 148.10 (C=N), 163.53 (d, ^1^*J*_C–F_ = 249.9 Hz), 163.95 (d, ^1^*J*_C–F_ = 249.9 Hz). IR (ATR,_max_, cm^−1^): 3050, 2948, 1739, 1603, 1531, 1511, 1472, 1462, 1432, 1415, 1380, 1310, 1297, 1286, 1226, 1155, 1125, 1099, 1065, 1055, 1015, 953, 923, 886, 860, 841, 825, 807, 784, 749, 724, 711, 676, 659, 630, 606. HR-MS (ESI^+^): *m*/*z* calcd for C_20_H_13_ClF_2_N_2_O [M + H]^+^ 371.0726; found: 371.0754. Elemental analysis calcd. for C_20_H_13_ClF_2_N_2_O: C, 64.79, H, 3.53, N, 7.56; found: C, 64.75, H, 3.55, N, 7.58.

##### 6-Chloro-1-((4-chlorobenzyl)oxy)-2-(4-chlorophenyl)-1*H*-benzo[*d*]imidazole (9)


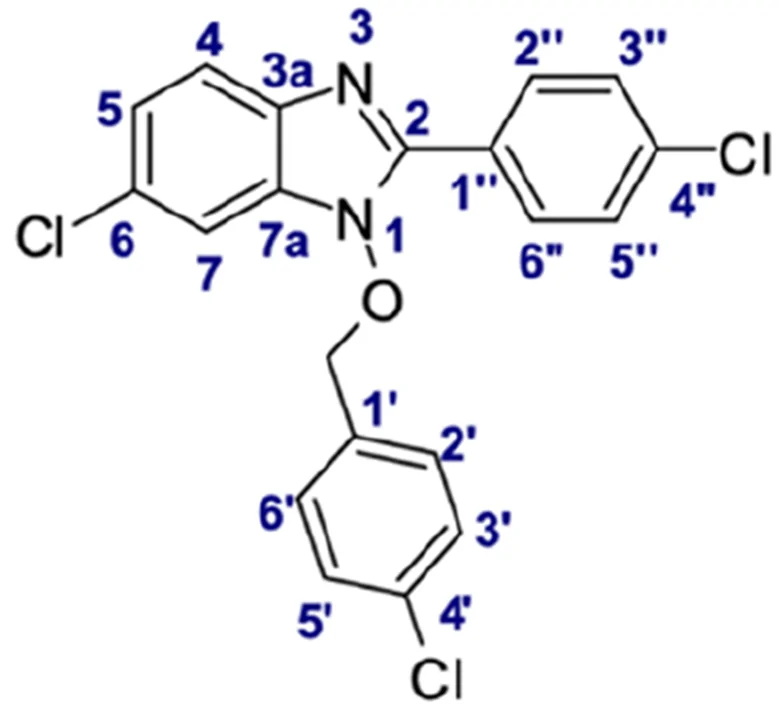
According to the general procedure by employing 5-chloro-2-nitroaniline (CAS no. 1635-61-6) and 4-chlorobenzyl bromide (CAS no. 622-95-7) to afford the title compound 9 (CAS no. 338978-84-0) as white powder. Yield: 57% (274 mg, 0.68 mmol), mp 174–176 °C. ^1^H NMR (500 MHz, CDCl_3_): *δ* 5.0 (s, 2H, –CH_2_–), 7.0 (d, ^3^*J* = 8.8 Hz, 2H, H-2′ and H-6′), 7.20 (d, ^3^*J* = 8.31 Hz, 2H, H-3′ and H-5′), 7.29 (dd, ^3^*J* = 8.3 Hz, ^4^*J* = 1.96 Hz, 1H, H-5), 7.33 (d, ^3^*J* = 8.3 Hz, 1H, H-4), 7.45 (d, ^3^*J* = 8.3 Hz, 2H, H-3″ and H-5″), 7.74 (d, ^4^*J* = 2.0 Hz, 1H, H-7), 7.96 (d, ^3^*J* = 8.8 Hz, 2H, H-2″ and H-6″). ^13^C NMR (126 MHz, CDCl_3_): *δ* 79.42 (–CH_2_–), 109.63, 120.20, 124.19, 126.51, 128.81, 128.84, 128.90, 129.75, 131.12, 136.11, 136.70, 191.13, 147.85 (C=N). IR (ATR,_max_, cm^−1^): 2948, 1739, 1600, 1512, 1494, 1462, 1431, 1411, 1379, 1312, 1277, 1241, 1171, 1125, 1092, 1056, 1015, 953, 923, 879, 861, 844, 834, 810, 787, 767, 749, 729, 716, 673, 661, 632, 606. HR-MS (ESI^+^): *m*/*z* calcd for C_20_H_13_Cl_3_N_2_O [M + H]^+^ 403.0171; found: 403.0166. Elemental analysis calcd. for C_20_H_13_Cl_3_N_2_O: C, 59.51, H, 3.25, N, 6.94; found: C, 59.54, H, 3.27, N, 6.90.

##### 6-Chloro-1-((4-methylbenzyl)oxy)-2-(*p*-tolyl)-1*H*-benzo[*d*]imidazole (10)


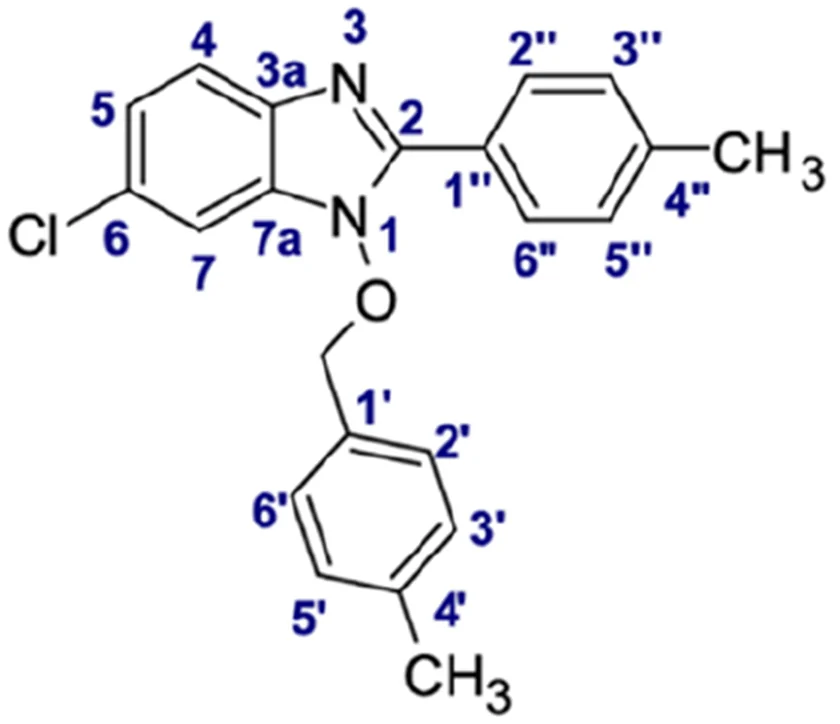
According to the general procedure by employing 5-chloro-2-nitroaniline (CAS no. 1635-61-6) and 4-methylbenzyl bromide (CAS no. 104-81-4) to afford the title compound 10 as off-white powder. Yield: 63% (329 mg, 0.91 mmol). Mp. 160–162 °C. ^1^H NMR (500 MHz, CDCl_3_): *δ* 2.24 (s, 3H, –CH_3_-4′), 2.45 (s, 3H, –CH_3_-4″), 4.96 (s, 2H, –CH_2_–), 7.10 (br s, 4H, H-2′, H-3′, H-5′ and H-6′), 7.23–7.24 (m, 1H, H-4), 7.27–7.28 (m, 1H, H-5), 7.31 (d, ^3^*J* = 7.8 Hz, 2H, H-3″ and H-5″), 7.73 (br s, 1H, H-7), 8.06 (d, ^3^*J* = 7.8 Hz, 2H, H-2″ and H-6″). ^13^C NMR (126 MHz, CDCl_3_): *δ* 21.27 (–CH_3_), 21.54 (–CH_3_), 80.20, 109.61, 119.83, 123.53, 125.41, 128.23, 128.39, 129.37, 129.73, 129.97, 130.19, 139.24, 139.76, 140.76, 148.86 (C=N). IR (ATR,_max_, cm^−1^): 2919, 1739, 1573, 1517, 1472, 1455, 1413, 1380, 1312, 1284, 1243, 1183, 1167, 1114, 1065, 1052, 1022, 953, 919, 880, 851, 820, 807, 797, 778, 747, 716, 679, 658, 608. HR-MS (ESI^+^): *m*/*z* calcd for C_22_H_19_ClN_2_O [M + H]^+^ 363.1264; found: 363.1258. Elemental analysis calcd. for C_22_H_19_ClN_2_O: C, 72.82, H, 5.28, N, 7.72; found: C, 72.85, H, 5.24, N, 7.69.

##### 6-Chloro-1-((4-(trifluoromethyl)benzyl)oxy)-2-(4-(trifluoromethyl)phenyl)-1*H*-benzo[*d*]imidazole (11)


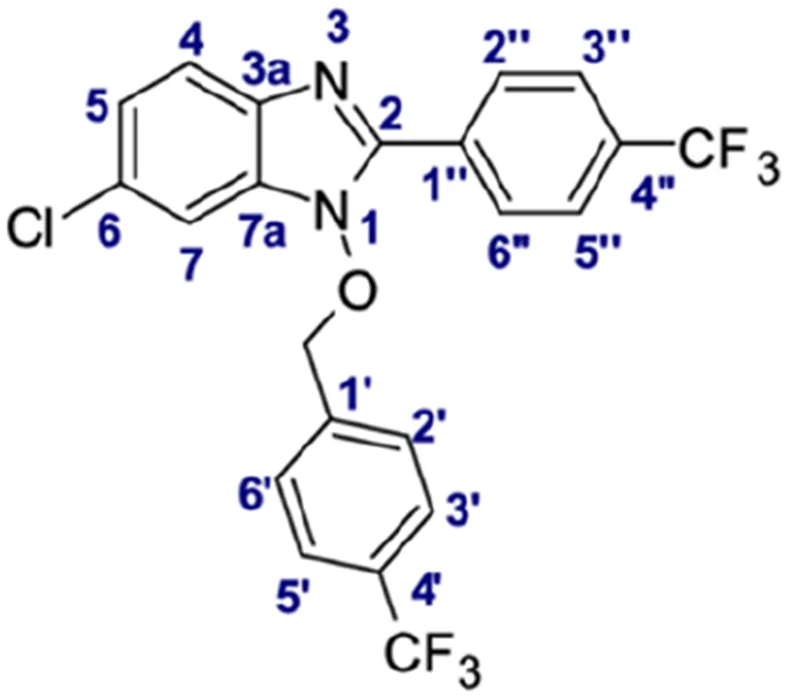
According to the general procedure by employing 5-chloro-2-nitroaniline (CAS no. 1635-61-6) and 4-(trifluoromethyl)benzyl bromide (CAS no. 402-49-3) to afford the title compound 11 as pale yellow powder. Yield: 47% (323 mg, 0.67 mmol), mp 142–144 °C. ^1^H NMR (500 MHz, CDCl_3_): *δ* 5.11 (s, 2H, –CH_2_–), 7.12 (d, ^3^*J* = 8.3 Hz, 2H), 7.34 (dd, ^3^*J* = 8.3 Hz, ^4^*J* = 2.0 Hz, 1H, H-5), 7.39 (d, ^3^*J* = 8.8 Hz, 1H, H-4), 7.42 (d, ^3^*J* = 8.3 Hz, 2H), 7.67 (d, ^3^*J* = 8.3 Hz, 2H, H-3″ and H-5″), 7.78 (d, ^4^*J* = 2.0 Hz, 1H, H-7), 8.03 (d, ^3^*J* = 8.3 Hz, 2H, H-2″ and H-6″). ^13^C NMR (126 MHz, CDCl_3_): *δ* 79.36 (–CH_2_–), 109.73, 120.60, 122.43, 122.66, 124.60, 124.69, 124.80, 125.43 (q, ^3^*J*_C–F_ = 3.9 Hz), 125.454 (q, ^3^*J*_C–F_ = 3.9 Hz), 128.79, 129.10, 129.45, 130.34, 131.96 (d, ^2^*J*_C–F_ = 32.4 Hz), 132.01 (d, ^2^*J*_C–F_ = 33.4 Hz), 135.76, 139.31, 147.60 (C=N). IR (ATR,_max_, cm^−1^): 2969, 1739, 1621, 1560, 1461, 1432, 1416, 1320, 1246, 1160, 1107, 1066, 1058, 1016, 960, 940, 924, 886, 847, 824, 798, 751, 734, 709, 695, 685, 676, 653, 600. HR-MS (ESI^+^): *m*/*z* calcd for C_22_H_13_ClF_6_N_2_O [M + H]^+^ 471.0699; found: 471.0693. Elemental analysis calcd. for C_22_H_13_ClF_6_N_2_O: C, 56.13, H, 2.78, N, 5.95; found: C, 56.15, H, 2.79, N, 5.98.

##### 6-Chloro-1-((3,5-dimethylbenzyl)oxy)-2-(3,5-dimethylphenyl)-1*H*-benzo[*d*]imidazole (12)


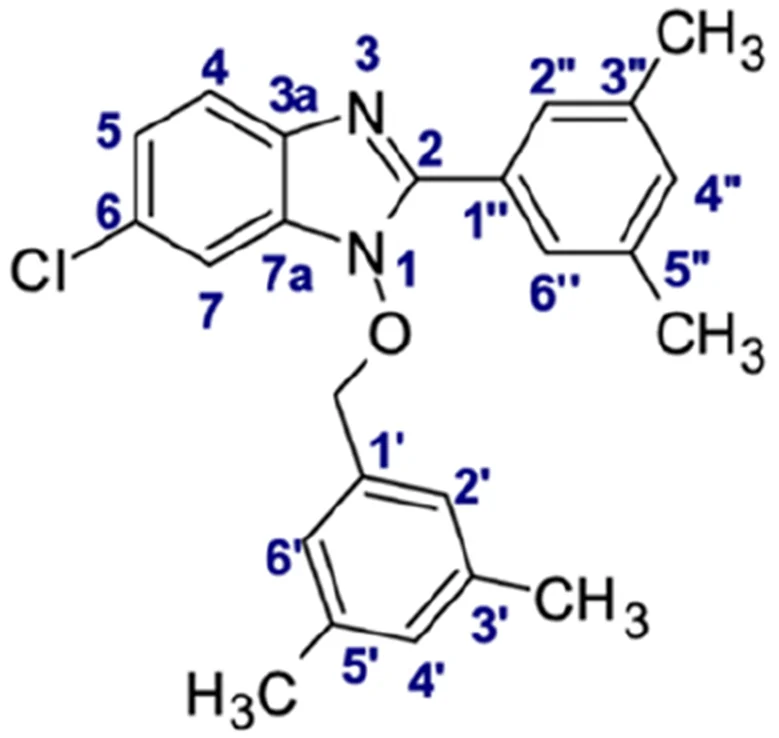
According to the general procedure by employing 5-chloro-2-nitroaniline (CAS no. 1635-61-6) and 3,5-dimethylbenzyl bromide (CAS no. 27129-86-8) to afford the title compound 12 as off-white powder. Yield: 50% (285 mg, 0.73 mmol), mp 137–139 °C. ^1^H NMR (500 MHz, CDCl_3_): *δ* 2.27 (s, 6H, –CH_3_-3′ and –CH_3_-5′), 2.41 (s, 6H, –CH_3_-3″ and –CH_3_-5″), 4.93 (s, 2H, –CH_2_–), 6.83 (br s, 2H, H-2′ and H-6′), 7.0 (br s, 1H, H-4′), 7.15 (br s, 1H, H-4″), 7.27 (dd, ^3^*J* = 8.3 Hz, ^4^*J* = 1.5 Hz, H-5), 7.35 (d, ^3^*J* = 8.3 Hz, H-4), 7.75 (d, ^4^*J* = 1.5 Hz, H-7), 7.80 (br s, 2H, H-2″ and H-6″). ^13^C NMR (126 MHz, CDCl_3_): *δ* 21.08 (–CH_3_), 21.26 (–CH_3_), 80.60 (–CH_2_–), 109.59, 119.92, 123.61, 126.23, 127.88, 127.93, 128.27, 130.13, 131.20, 132.11, 132.43, 138.20, 138.36, 139.25, 149.04 (C=N). IR (ATR,_max_, cm^−1^): 2916, 1739, 1603, 1455, 1416, 1373, 1306, 1277, 1227, 1155, 1125, 1085, 1053, 932, 912, 883, 876, 856, 843, 782, 749, 714, 704, 675, 655, 603. HR-MS (ESI^+^): *m*/*z* calcd for C_24_H_23_ClN_2_O [M + H]^+^ 391.1577; found: 391.1571. Elemental analysis calcd. for C_24_H_23_ClN_2_O: C, 73.74, H, 5.93, N, 7.17; found: C 73.71, H 5.97, N 7.19.

##### 6-Chloro-1-((3-chloro-4-fluorobenzyl)oxy)-2-(3-chloro-4-fluorophenyl)-1H-benzo[d]imidazole (13)


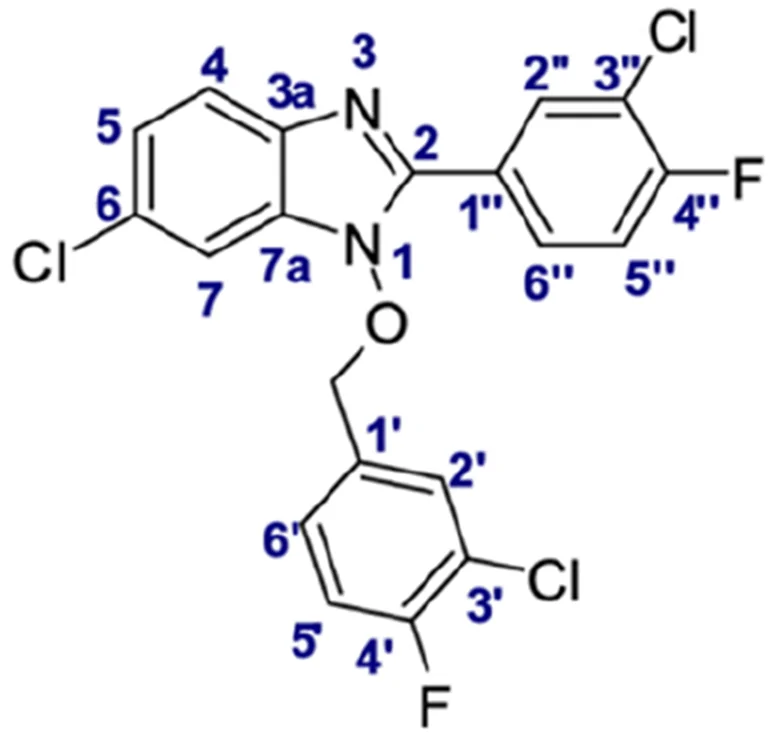
According to the general procedure by employing 5-chloro-2-nitroaniline (CAS no. 1635-61-6) and 3-chlorobenzyl bromide (CAS no. 766-80-3) to afford the title compound 13 as pale yellow powder. Yield: 31% (198 mg, 0.45 mmol), mp 142–144 °C. ^1^H NMR (500 MHz, CDCl_3_): *δ* 5.00 (s, 2H, –CH_2_–), 6.85–6.87 (m, 1H, H-2′), 6.99 (t, ^3^*J* = 8.3, 1H, H-5′), 7.16 (d, ^3^*J* = 8.3 Hz, 1H, H-6′), 7.24 (t, ^3^*J* = 7.3 Hz, 1H, H-5″), 7.32 (d, ^3^*J* = 8.8 Hz, 1H, H-5), 7.37 (d, ^3^*J* = 8.8 Hz, 1H, H-4), 7.75 (br s, 1H, H-7), 7.86–7.88 (m, 1H, H-2″), 8.05 (d, ^3^*J* = 7.3 Hz, 1H, H-6″). ^13^C NMR (126 MHz, CDCl_3_): 78.84 (–CH_2_–), 109.62, 116.90 (d, ^2^*J*_C–F_ = 21.9 Hz), 116.8 (d, ^2^*J*_C–F_ = 21.0 Hz), 120.41, 121.63 (d, ^2^*J*_C–F_ = 18.1 Hz), 121.67 (d, ^2^*J*_C–F_ = 18.1 Hz), 124.49, 125.18 (d, ^4^*J*_C–F_ = 3.8 Hz), 128.53 (d, ^3^*J* = 7.6 Hz), 129.23 (d, ^3^*J* = 4.8 Hz), 129.4, 129.99 (d, ^3^*J* = 7.6 Hz), 130.79, 132.64, 139.14, 146.84 (C=N), 158.98 (d, ^1^*J*_C–F_ = 253.7 Hz), 159.25 (d, ^1^*J*_C–F_ = 254.6 Hz). IR (ATR,_max_, cm^−1^): 2921, 1739, 1580, 1502, 1472, 1429, 1415, 1380, 1363, 1312, 1286, 1253, 1184, 1168, 1125, 1114, 1052, 1022, 955, 922, 899, 880, 853, 818, 808, 793, 784, 748, 716, 692, 679, 659, 623, 609. HR-MS (ESI^+^): *m*/*z* calcd for C_20_H_11_Cl_3_F_2_N_2_O [M + H]^+^ 438.9983; found: 438.9983. Elemental analysis calcd. for C_20_H_11_Cl_3_F_2_N_2_O: C, 54.64, H, 2.52, N, 6.37; found: C, 54.61, H, 2.54, N, 6.33.

### Relative lipophilicity determination

The experimental determination of relative lipophilicity was performed as previously described,^26^ with minor modifications. Further details are reported in the SI.

## Results and discussion

As part of our ongoing studies on a series of *N*-aryl-benzimidazoles, previously reported as inhibitors of (pancreatic porcine) elastase^26^ and proposed as new pharmacological tools for the development of newer agents for the treatment of skin pathologies, we encountered an unexpected finding during their multistep synthesis. Specifically, in the first step of the synthetic procedure, we serendipitously isolated an unexpected by-product that was subsequently identified as an *N*-oxide-benzimidazole chemotype as confirmed by NMR, IR and HRMS analyses.

Given that the benzimidazole scaffold is recognized as a privileged structure for glucosidase inhibition,^12^ we hypothesized that this serendipitously identified chemotype could represent a promising starting point for the development of new glucosidase inhibitors. Building on this insight, we designed and synthesized a small series of analogues, as detailed in the chemistry section, with the aim to explore the effect of halogen substitution on the benzimidazole nucleus together with the influence of differently substituted benzyl and phenyl moieties on the physicochemical and biological properties of the target compounds.

### Biochemical data & SAR exploration

We evaluated the inhibitory activity of the target compounds 1–13 against α-glucosidase according to previously reported protocols,^27^ as briefly described in the experimental section, and the results are summarized in Table 1. In more detail, compounds 1–5 were selected for an initial screening campaign aimed at investigating the effect of substituents on the benzene-fused ring of the benzimidazole scaffold. The unsubstituted derivative (1) was used as the starting point, while the influence of halogen substitution was explored through the introduction of fluorine or chlorine atoms at the 5- or 6-position of the benzimidazole nucleus. The preliminary structure–activity relationship (SAR) study revealed that there was a clear impact on the inhibitory activity by the nature and position of the halogen substituent. Among the tested derivatives, compound 5, bearing a chlorine atom at the 6-position, emerged as the most active inhibitor, displaying 55.14 ± 0.90% inhibition at a fixed concentration of 50 μM. Based on this result, the 6-chlorobenzimidazole motif was selected as the preferred core for further optimization. Subsequent efforts were therefore directed toward the modulation of the 2-phenyl moiety and the *N*-1-benzyloxy substituent through the introduction of halogens (F, Cl) or methyl groups at the *meta* and *para* positions of the aromatic rings, affording compounds 6–13. The substitution patterns for these groups are always the same due to the synthetic procedure. As shown in Table 1, compounds 6 and 7, 9 and 10, and 12 and 13 exhibited inhibitory activity against α-glucosidase. Unfortunately, compounds 8 and 11 could not be accurately evaluated due to their limited solubility in the assay medium; they were reported as not tested (N.T.) in Table 1. Compounds 6 and 13 emerged as the most potent AGIs, displaying inhibition percentages of 81.37 ± 0.90% and 99.35 ± 1.81%, respectively. Given their remarkable activity, their IC_50_ values were subsequently determined and compared with that of acarbose, used as a reference inhibitor.^12^

**Table 1:** Chemical structure, substitution pattern and inhibitory activity for the synthesized compounds 1–13 and acarbose against α-glucosidase

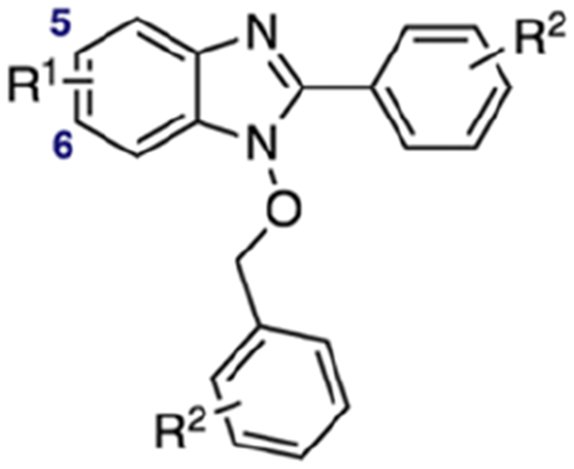				
Cmpd. name	R^1^	R^2^	α-Glucosidase	IC_50_ (μM)
			% inib. @50 μM	
1	H	H	13.07 ± 3.18	N.D.
2	5-F	H	5.96 ± 4.11	N.D.
3	6-F	H	24.09 ± 2.30	N.D.
4	5-Cl	H	17.26 ± 4.41	N.D.
5	6-Cl	H	55.14 ± 0.90	N.D.
6	6-Cl	3-Cl	81.37 ± 0.90	32.35 ± 0.35
7	6-Cl	3-CH_3_	70.88 ± 0.83	N.D.
8	6-Cl	4-F	N.T.^a^	N.D.
9	6-Cl	4-Cl	65.58 ± 3.37	N.D.
10	6-Cl	4-CH_3_	67.50 ± 9.69	N.D.
11	6-Cl	4-CF_3_	N.T.^a^	N.D.
12	6-Cl	3,5-(CH_3_)_2_	73.68 ± 3.14	N.D.
13	6-Cl	3-Cl, 4-F	99.35 ± 1.81	15.7 ± 0.28
**Acarbose**	—	—	—	139 ± 11.3

a Not tested because of limited solubility in the assay medium. N.D, not determined. IC_50_ values were determined for compounds showing ≥80% inhibition at the tested concentration of 50 μM.

Compound 6 exhibited an IC_50_ value of 32.35 ± 0.35 μM, whereas compound 13 proved to be the most potent derivative of the series showing an IC_50_ value of 15.70 ± 0.28 μM. Notably, 6 and 13 displayed greater inhibitory potency than the reference inhibitor acarbose (IC_50_ value of 139.0 ± 11.3 μM) in the same test. Overall, the SAR findings suggest that the deliberate substitution on the benzimidazole core and the two pendant aromatic moieties might represent a key structural feature for improving α-glucosidase inhibitory activity. Further, more detailed studies have shown that the Lineweaver–Burk plots indicated that both compounds 6 and 13 exhibited uncompetitive inhibition, as the kinetic analysis yields a family of parallel lines with increasing compound concentration (Fig. 2). The equilibrium constant for binding to the enzyme–substrate complex (*K*_IS_) was determined by replotting the intercepts (1/*V*_max_) against inhibitor concentration, resulting in IC_50_ values of 18.03 and 4.23 μM for compounds 6 and 13, respectively (Fig. 3).

**Fig. 2:**
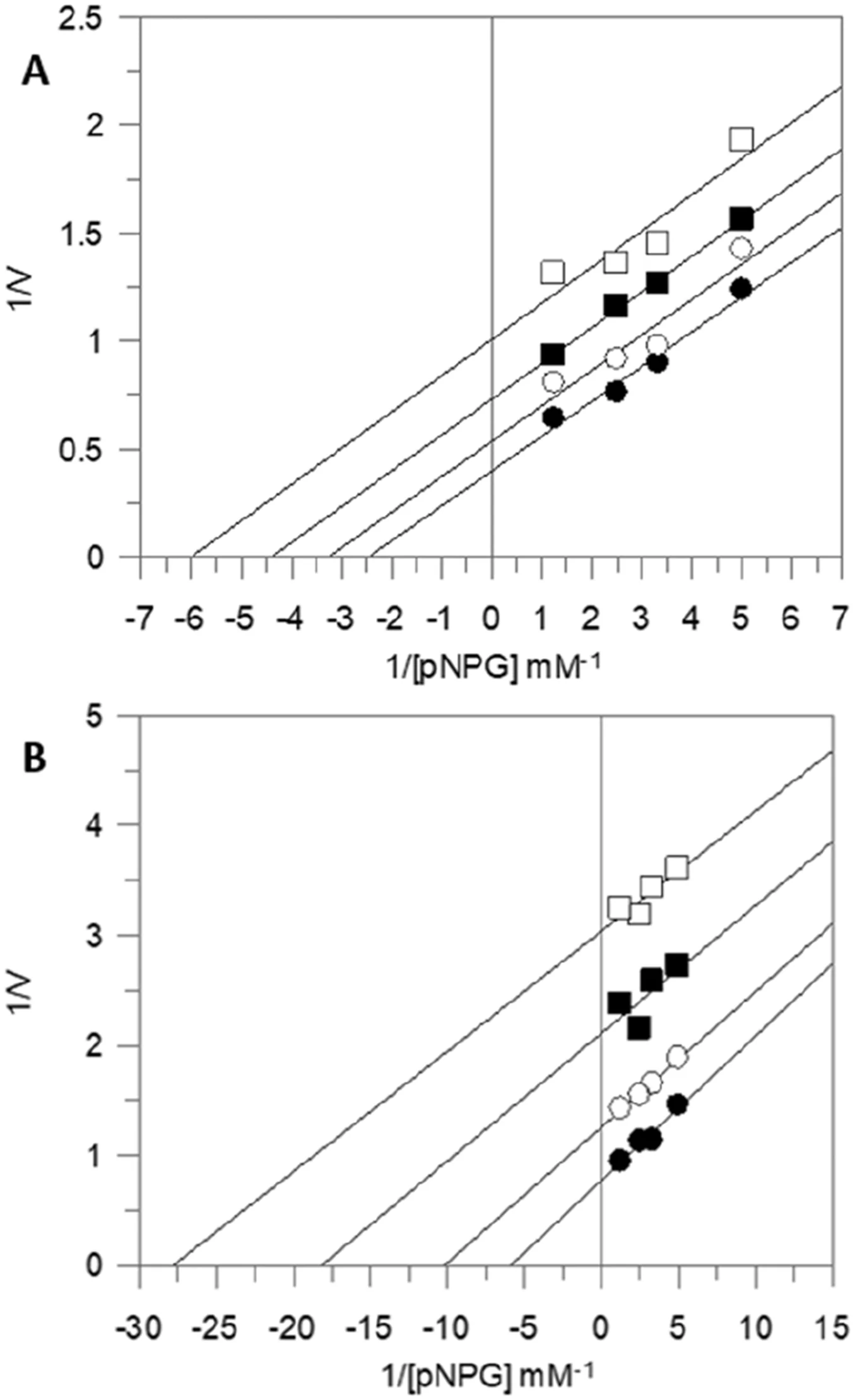
Inhibition of α-glucosidase activity. The Lineweaver–Burk analysis using concentrations of 0 (●), 10 (○), 20 (■) and 30 (□) μM of compound 6 (panel A) and concentrations of 0 (●), 5 (○), 10 (■) and 15 (□) μM of compound 13 (panel B).

**Fig. 3:**
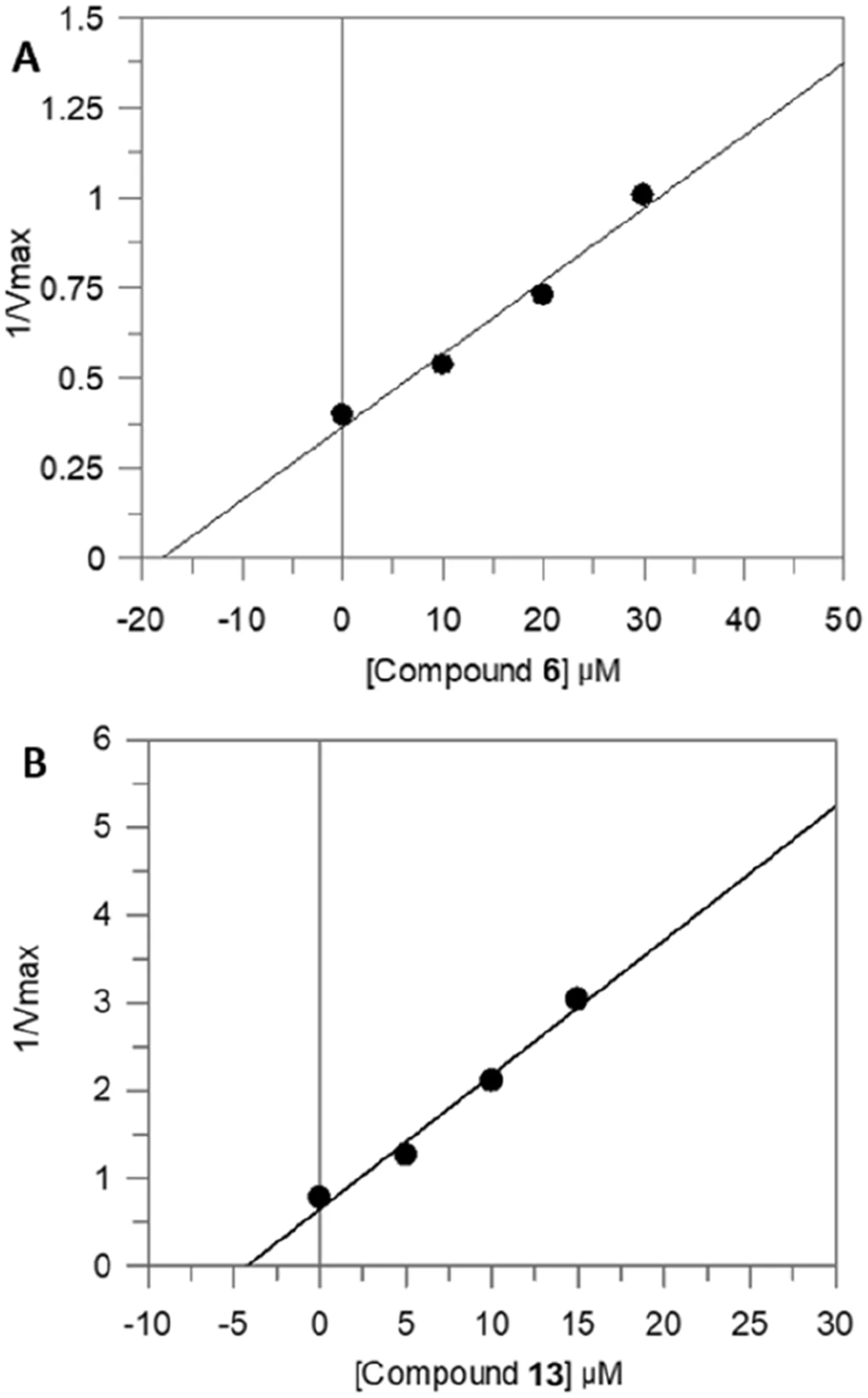
Inhibition of α -glucosidase activity. Secondary plots using different concentrations of compounds 6 (panel A) and 13 (panel B).

It is well-known that the development of selective α-glucosidase inhibitors, characterized by reduced activity against α-amylase, represents a promising approach for controlling postprandial hyperglycemia. This is because excessive α-amylase inhibition has been associated with gastrointestinal side effects, resulting from increased transit of undigested carbohydrates to the colon and subsequent fermentation by the gut microbiota.^18,19^ Therefore, compounds 6 and 13 demonstrating the highest inhibitory activity against α-glucosidase were subsequently assessed for their effects on α-amylase. Notably, neither compound exhibited significant activity against α-amylase (10% inhibition under the same experimental conditions as the α-glucosidase assay), indicating significant selectivity for α-glucosidase.

### Cell viability and cellular antioxidant activity

Compounds 6 and 13 exhibiting high inhibitory activity against α-glucosidase were subjected to cytotoxicity testing, as assessing cytotoxicity provides essential information on their cellular safety and potential to induce cell damage, thereby supporting the evaluation of their therapeutic potential. Caco-2 cells were used for this assay because they are derived from human colon adenocarcinoma and exhibit structural and functional characteristics like those of differentiated intestinal epithelial cells; furthermore, they contain enzymes typical of the small-intestinal brush-border epithelium. As shown in Fig. 4, the treatment with compound 6 maintained cell viability above 80% across all tested concentrations, including the highest concentration of 60 μM. Compound 13 preserved complete cell viability across all concentrations evaluated.

**Fig. 4:**
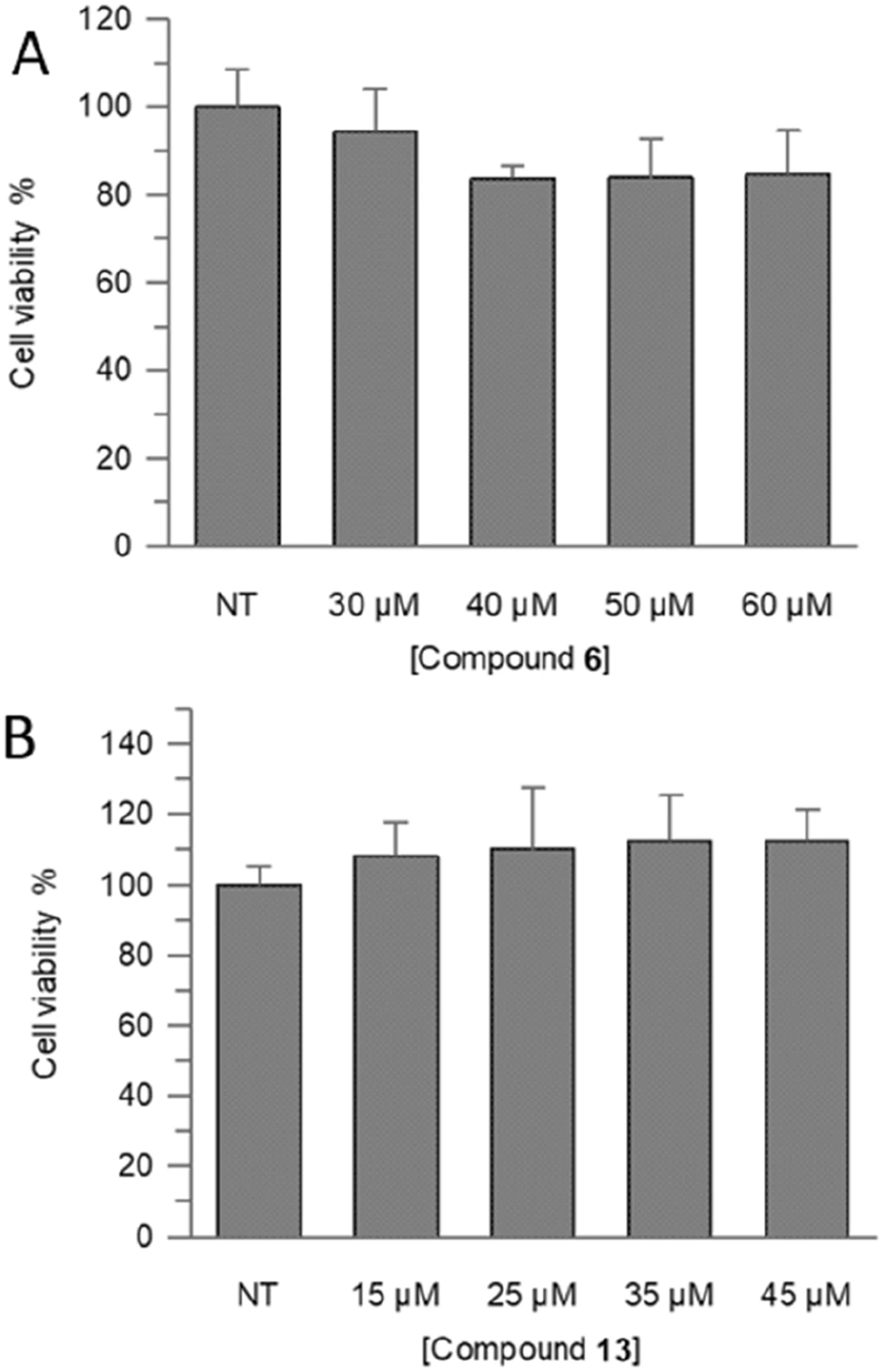
Effects of compounds 6 and 13 on Caco-2 cell viability using the MTT assay. Cells were treated with different concentrations of compounds 6 (A) and 13 (B). Data are expressed as a percentage of the control. Data represent the mean (±standard deviation) of six independent experiments.

As previously mentioned, altered glucose metabolism due to hyperglycemia leads to ROS production; accordingly, compounds 6 and 13 were evaluated for their antioxidant capacity at the cellular level (see Fig. 5). Both compounds significantly decreased H_2_O_2_-induced ROS production in a dose-dependent manner. Antioxidant effects were evident at all tested concentrations, including those equivalents to their IC_50_ values for α-glucosidase inhibition. Compound 13 exhibited antioxidant activity at a concentration of 15 μM, confirming that it is also one of the most active compounds in terms of antioxidant efficacy.

**Fig. 5:**
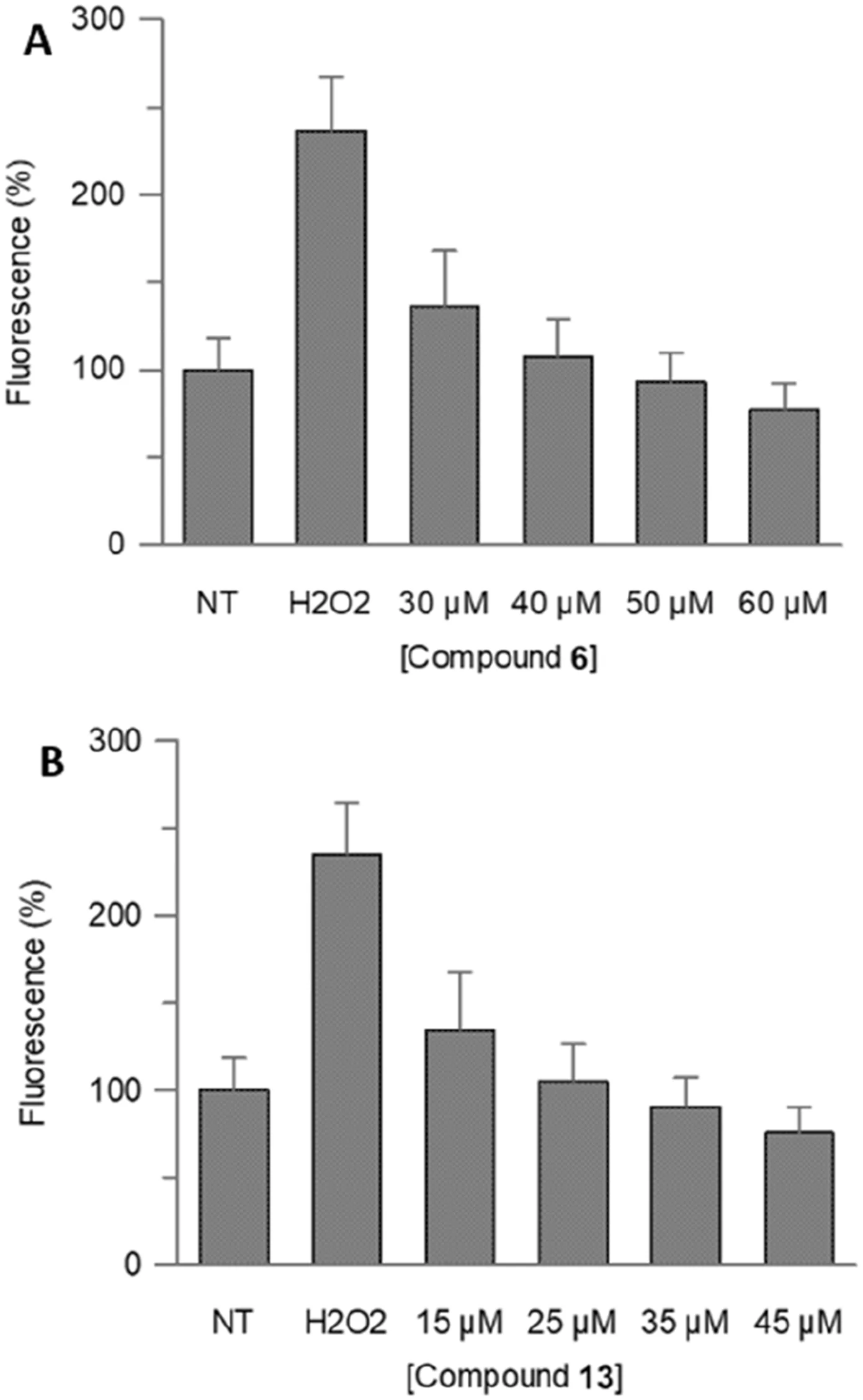
ROS levels (expressed as fluorescence) in Caco-2 cells pretreated with different concentrations of compounds 6 (A) and 13 (B) incubated with 2 mM of H_2_O_2_ up to 120 min. NT for non-treated cells; H_2_O_2_ for cells only treated with H_2_O_2_. Data represent the mean (±standard deviation) of six independent experiments.

### Computational studies

The binding mode of the most active compounds, 6 and 13, was elucidated by molecular docking performed on a 3D structural model of the α-glucosidase protein from *Saccharomyces cerevisiae*, predicted by the AlphaFold webserver, in complex with the substrate pNPG. According to kinetic studies, both inhibitors exhibited an uncompetitive inhibition mechanism, indicating that they preferentially bind to the enzyme–substrate complex. This interaction hinders the conformational changes required for catalysis, thus locking the substrate within the active site and preventing the hydrolytic process. To gain structural insights into the observed inhibition mode, a binding site search was conducted to identify putative druggable pockets distinct from the catalytic site by using two different tools, namely SiteMap and FTMap. Interestingly, both approaches identified a common druggable pocket located at the entrance of the active site (Fig. 6). These results agree with previously reported computational studies performed on uncompetitive and mixed-type α-glucosidase inhibitors, which reported binding to the same protein region.^16,28^ Notably, this consensus binding site was ranked first by both algorithms, supporting its druggability and potential functional relevance. Based on these outcomes, molecular docking studies were focused on this pocket.

**Fig. 6:**
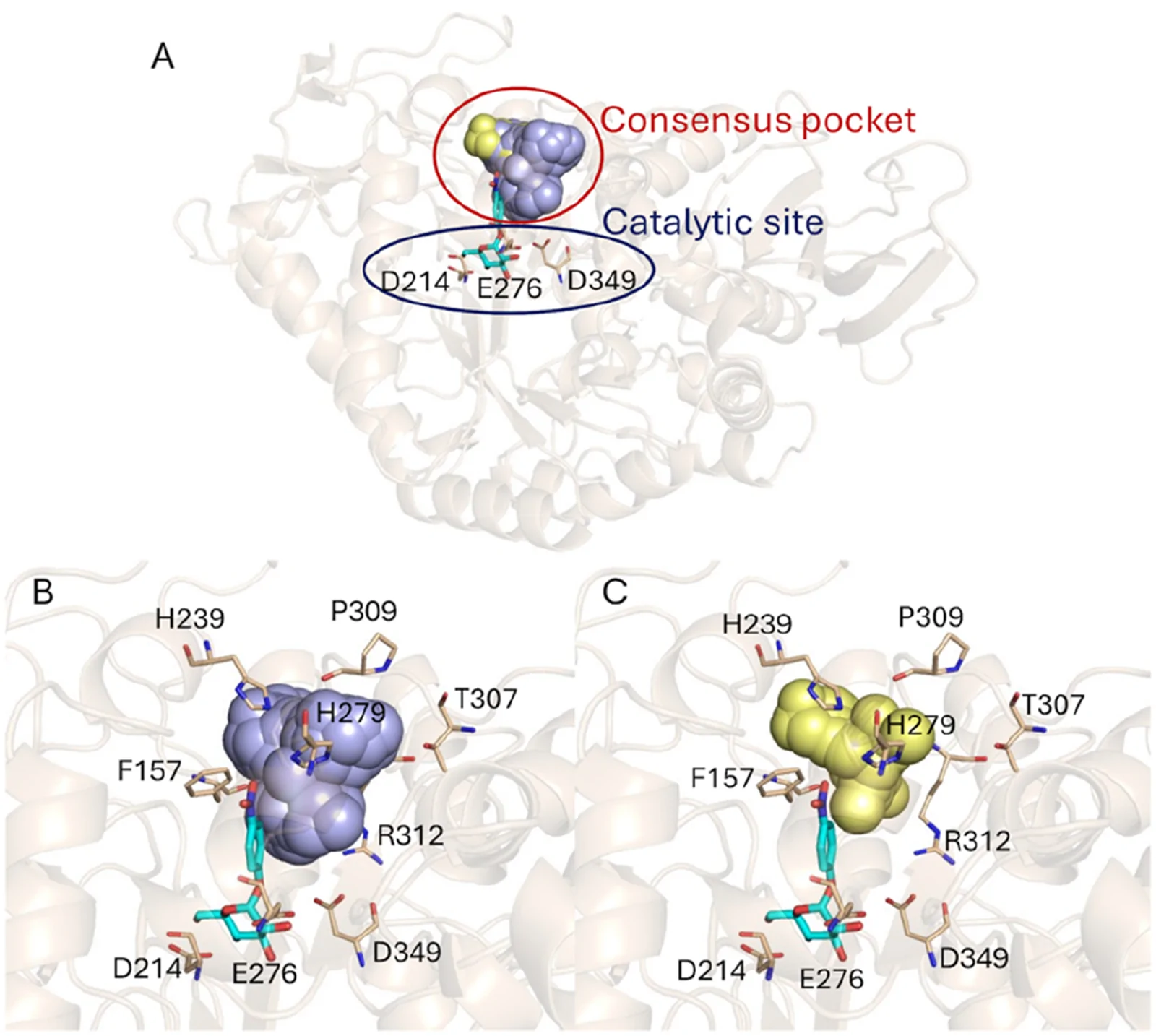
Overview of the consensus druggable binding site defined by the overlap between the binding regions, highlighted as spheres, identified by SiteMap (violet spheres) and FTMap (yellow spheres) (A). Close view of the top-ranked binding sites predicted by SiteMap (B) and FTMap (C). The substrate pNPG is displayed as cyan sticks.

The results suggested that both inhibitors 6 and 13 could interact with α-glucosidase assuming similar binding modes as shown in Fig. 7A and B, respectively. In more detail, the benzyloxy moiety was implicated in aromatic interactions with H279 and H239, while the chlorine substituent allowed for additional hydrophobic contacts with H239 and F231. The benzimidazole system established hydrophobic interactions with P309 and an H-bond with T307. Additionally, the chlorine atom at the C6-position engaged in hydrophobic contacts with F157, further stabilizing the ligand–protein complex. Finally, the C2-phenyl ring was involved in hydrophobic interactions with P309. For comparison purposes, docking simulation was also carried out for compounds 1 (Fig. 7C) and 2 (Fig. 7D), which exhibited the weakest α-glucosidase inhibitory activity. The results showed that the absence of the chlorine atom at the C6 position induced a nearly 180° rotation of the 2-phenylbenzimidazole scaffold relative to the binding orientation predicted for compounds 6 and 13. Despite this conformational change, the benzimidazole moiety retained both the hydrophobic interaction with P309 and the hydrogen bond with T307. Instead, the benzyloxy moiety maintained the same orientation observed for the most active derivatives, preserving the aromatic interactions with H279 and H239. However, the lack of chlorine substituents on both the benzyloxy ring and the benzimidazole nucleus hampered the formation of additional hydrophobic contacts, resulting in a less extensive interaction network within the binding pocket. A 2D interaction diagram of the ligand–protein interactions is provided in Fig. S35 of the SI.

**Fig. 7:**
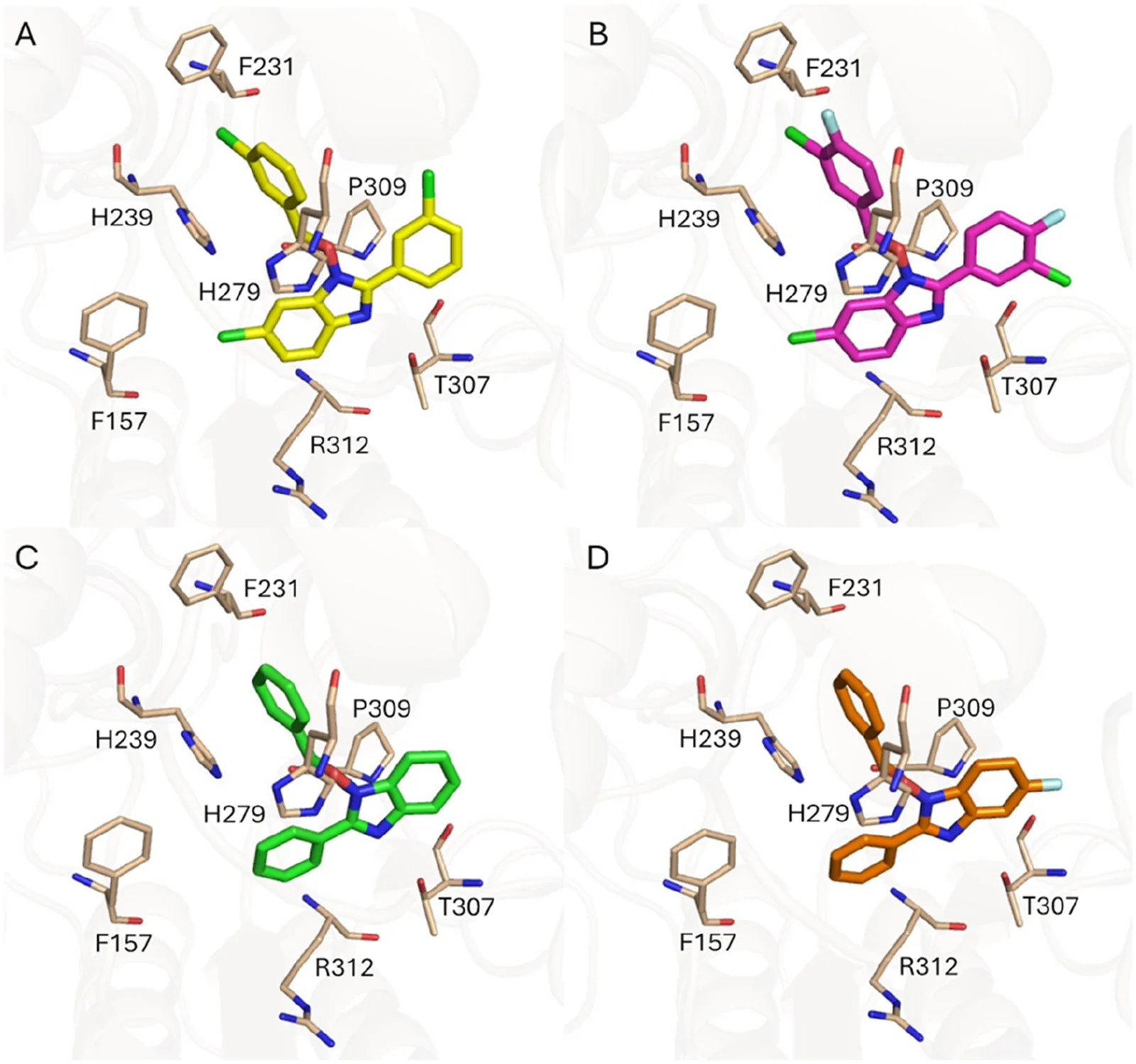
Docking poses of compounds 6 (A), 13 (B), 1 (C) and 2 (D) within the consensus pocket identified by SiteMap and FTMap.

Overall, these findings suggested that chlorine substitution on the C6-position plays a critical role in enhancing α-glucosidase inhibition by strengthening ligand–enzyme interactions and increasing complex stability. The additional contacts elicited by compounds 6 and 13 within the proposed binding site located at the entrance of the catalytic pocket align well with their uncompetitive inhibition mechanisms as well as their highest inhibitory activity against α-glucosidase.

## Chemistry

The preparation of the target benzimidazoles 1–13 is outlined in Scheme 1. The synthetic route involved the reaction of substituted 2-nitroanilines 14–18 with benzyl bromides 19–27 in the presence of sodium hydride in dry THF under reflux conditions. Following the optimized reaction conditions, the desired benzimidazole *N*-oxide derivatives were obtained in moderate to good yields (39–83%) after 4–7 h of reaction time.

**Scheme 1:**
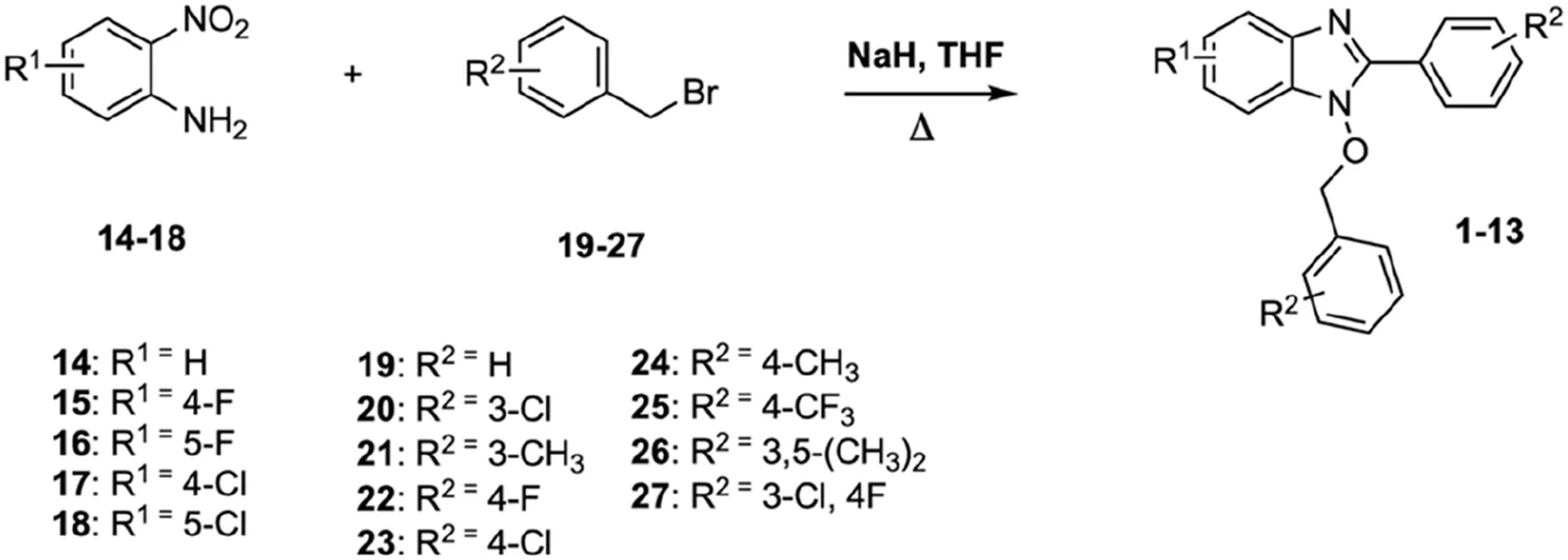
Synthesis of target compounds 1–13; reagents and conditions: NaH, THF, reflux, 4–7 h.

A noteworthy feature of this transformation is the occurrence of a one-pot alkylation-heterocyclization-*O*-alkylation cascade. Following the initial *N*-alkylation of the *o*-nitroaniline precursor, the intermediate rapidly undergoes cyclization to generate the benzimidazole *N*-oxide scaffold, which is subsequently trapped by *in situ O*-alkylation. Such tandem processes appear to have limited precedent in the literature.^29^ The reaction also displays a high degree of chemoselectivity. Despite the presence of an aniline NH group in the putative *N*-alkyl intermediates, no evidence of competitive *N*,*N*-dialkylation was obtained. Likewise, reaction monitoring did not reveal the accumulation of benzimidazole *N*-oxide intermediates or other partially transformed species. These observations suggest that, once initiated, the reaction rapidly proceeds through hetero-cyclization and subsequent *O*-alkylation (see additional details in the SI), preventing the accumulation of detectable intermediates and leading directly to the final benzimidazole *N*-oxide derivatives. The further attractive feature of the proposed synthetic protocol is the complete avoidance of chromatographic purification. Indeed, all target compounds were isolated in high purity by straightforward precipitation from *n*-hexane. Besides simplifying the work-up procedure, this approach offers clear advantages in terms of scalability, cost-effectiveness, and reduced environmental footprint, making the methodology particularly appealing for the preparation of compound libraries.

All synthesized compounds (1–13) were thoroughly characterized by ^1^H NMR, ^13^C NMR, HRMS, and ATR-FTIR spectroscopy to confirm their structures and assess their purity. The ^1^H and ^13^C NMR spectra were consistent with the proposed molecular structures. A 2D ^1^H–^13^C HSQC NMR spectrum was also acquired for compound 10, and is provided in the SI (Fig. S33). HRMS analyses provided molecular ion peaks in agreement with the calculated exact masses, further supporting the identity of the synthesized derivatives. In addition, ATR-FTIR spectroscopy confirmed the presence of the characteristic functional groups through the observation of the expected absorption bands. Complete spectroscopic and analytical data, including ^1^H NMR, ^13^C NMR, HRMS, and IR spectra, are provided in the experimental section (*vide infra*) and SI (Fig. S1–S32: ^1^H NMR and ^13^C NMR for compounds 1–13).

The relative lipophilicity of compounds 1–13 was determined by reverse phase thin layer chromatography (RP-TLC) following the previously reported method.^30^ The calculated lipophilicity parameters, expressed as *R*_M_ values, ranged from 0.219 to 0.865, revealing a relatively broad distribution of lipophilic properties within the series and highlighting the influence of structural modifications on this class of functionalized benzimidazoles. As expected, compound 12, bearing two 3,5-dimethylbenzyl moieties, displayed the highest lipophilicity, whereas the unsubstituted derivative 1 and the 5-halogen-substituted compounds 2 and 3 exhibited the lowest lipophilic character. The obtained lipophilicity data were subsequently compared with the values predicted using the SWISSADME platform (accession date June 17th, 2026, https://www.swissadme.ch).^30^ By means of this free web tool, we estimated other ADME parameters (Table S1 in the SI); all target compounds 1–13 were predicted to be free of PAINS alerts; in terms of drug-likeness, all compounds were generally estimated to display gastrointestinal absorption GI; however, they were predicted as high lipophilic molecules, thus featuring a violation of the Lipinski rule. Overall, these estimated data represent a strong limitation for the most active compounds 6 and 13, thus furnishing the prompt to design new analog compounds to overcome the lack of drug-likeness.

## Conclusions

This work highlighted new substituted benzimidazoles as selective AGIs. The results showed that appropriate substitution of both the benzimidazole core and the pendant aromatic rings improved inhibitory activity against α-glucosidase while maintaining negligible activity against α-amylase. This relevant selectivity was a particularly valuable feature and supported the further investigation of these compounds as targeted modulators of postprandial glucose control. Kinetic studies indicated an uncompetitive inhibition mechanism for the most active derivatives, while binding-site analysis and molecular docking suggested their interaction with a putative druggable pocket located at the entrance of the catalytic site. The predicted binding modes also highlighted the contribution of the chlorine substituent at the C-6 position of the benzimidazole core to an extended network of hydrophobic interactions, providing a plausible structural explanation for the higher activity of compounds 6 and 13. Overall, these findings offer a useful basis for the rational design of new analogues. Further studies should therefore focus on improving potency and physicochemical properties, particularly by reducing lipophilicity, while preserving α-glucosidase selectivity and clarifying the molecular determinants underlying recognition of the proposed binding region.

## Author contributions

Conceptualization, FM, RG, AF and LDL; methodology, FM, GP, SV, LI, CD, SF and AF; investigation, FM, LI, CD, SF and AF; formal analysis, GP, SV, SF and AF; software, GP and SV; validation, GP, SV, SF and AF; data curation, FM, GP, SV, LI, CD, SF and AF; writing – original draft, FM, RG and LDL; writing – review and editing, all authors; supervision, RG and LDL; all authors have read and approved the final version of the manuscript.

## Conflicts of interest

There are no conflicts to declare.

## Supplementary Material

MD-OLF-D6MD00752J-s001

## Data Availability

The data supporting this article have been included in the supplementary information (SI). Supplementary information: Fig. S1–S32: ^1^H NMR and ^13^C NMR spectra of synthetized compounds 1–13. Fig. S33: 2D ^1^H–^13^C HSQC NMR spectrum of compound 10. Table S1: structural and physicochemical descriptors of compounds 1–13, including SMILES notation, experimentally determined chromatographic retention parameter (*R*_M_), and selected SwissADME descriptors, including consensus log *P* values (clog*P*), gastrointestinal absorption, PAIN alert, and Lipinski rule violations. Fig. S34: Ramachandran plot of the generated α-glucosidase model predicted by AlphaFold3. Fig. S35: 2D depiction of the interactions between alpha glucosidase and compounds 6, 13, 1 and 2 predicted by Discovery Studio Visualizer. See DOI: https://doi.org/10.1039/d6md00752j.
