# Supplementary material for: Discovery of benzimidazole-based selective α-glucosidase inhibitors with an uncompetitive mode of action: synthesis, biological assays and computational studies
Source: RSC Med Chem. 2026 Oct 28. Online ahead of print. doi: 10.1039/d6md00752j (PMC13741480; doi:10.1039/d6md00752j)

Supplementary Information (SI) for RSC Medicinal Chemistry.
This journal is © The Royal Society of Chemistry

SUPPLEMENTARY INFORMATION

Discovery of benzimidazole-based selective α-glucosidase inhibitors with an uncompetitive mode of action: synthesis, biological assays and computational studies

Francesca Mancuso, *[a] Giovanna Pitasi [a], Sonia Floris, [b] Laura Ielo, [c] Serena Vittorio, [a] Christian Dank, [d,e] Rosaria Gitto, [a] Antonella Fais, [b] Laura De Luca,[a]

[a]Department of Chemical, Biological, Pharmaceutical and Environmental Sciences, University of Messina, Viale F. Stagno D'Alcontres 31, Messina I-98166, Italy
[b]Department of Life and Environment Sciences, University of Cagliari, Monserrato 09042, Cagliari, Italy
[c]Institute of Applied Synthetic Chemistry, TU Wien, Getreidemarkt 9, 1060 Vienna, Austria
[d]Department of Pharmaceutical Chemistry, University of Vienna, Josef-Holaubek-Platz 2, A-1090 Vienna, Austria
[e]Institute of Organic Chemistry, University of Vienna, Währinger Straße 38, A-1090 Vienna, Austria

Table of contents:

- **Figures 1S-19S**: ^{1}H-NMR spectra for the synthetized compounds **1-13**.
- **Figures 20S-32S**: ^{13}C-NMR spectra for the synthetized compounds **1-13**.
- **Figure 33S**: 2D ^{1}H–^{13}C HSQC NMR spectrum for compound **13**.
- Determination of lipophilicity by experimental method.
- **Table 1S**: Computational prediction of ADME properties an drug-likeness.
- **Figure 34S**: Ramachandran plot of the generated α-glucosidase model predicted by AlphaFold3.
- **Figure 35S**. 2D depiction of the interactions between alpha glucosidase and compounds **6**, **13**, **1** and **2** predicted by Discovery Studio Visualizer.

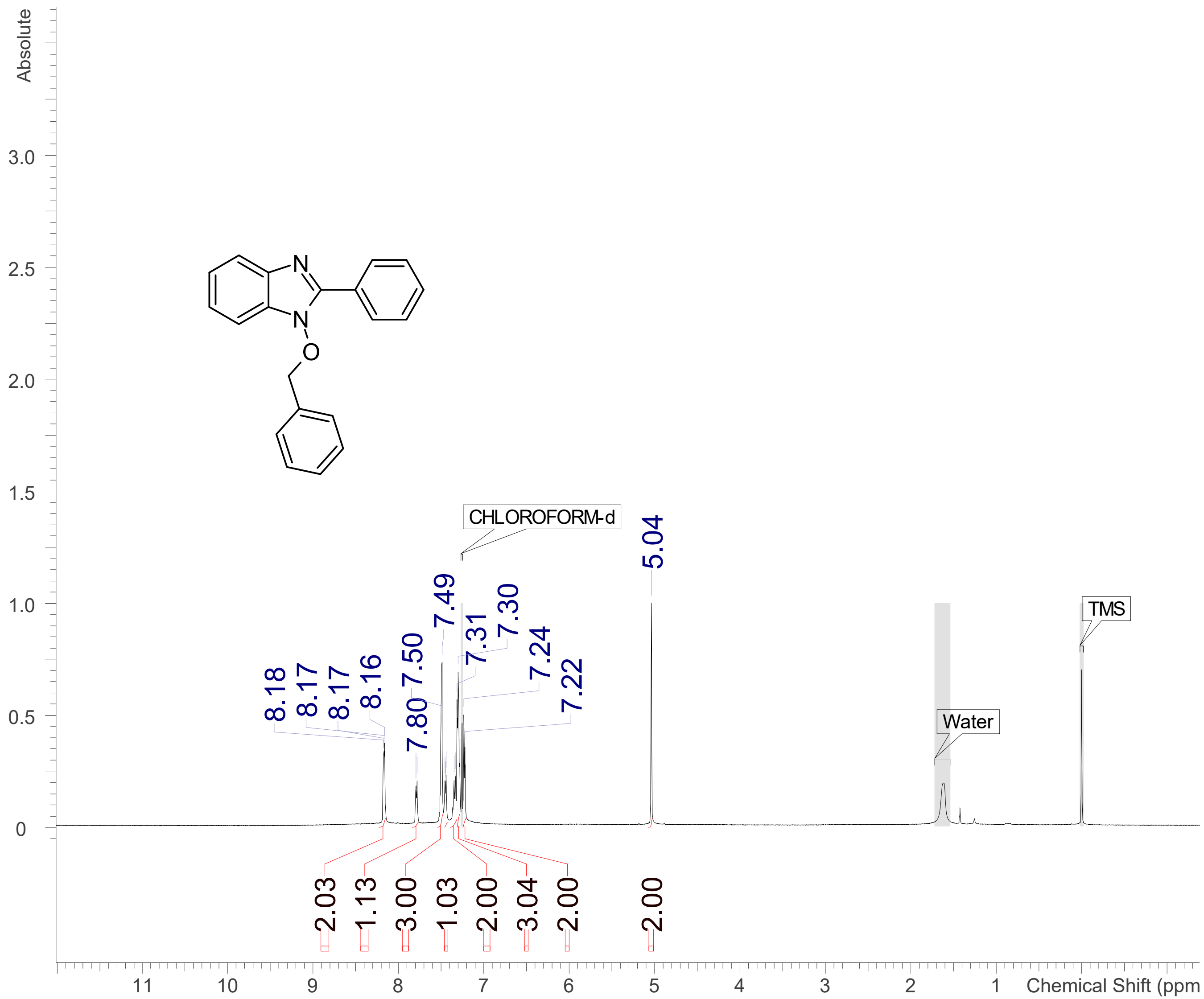

Figure 1S. ^{1}H NMR (500 MHz, CHLOROFORM-*d*) for 1-(Benzyloxy)-2-phenyl-1*H*-benzo[*d*]imidazole **(1)**.

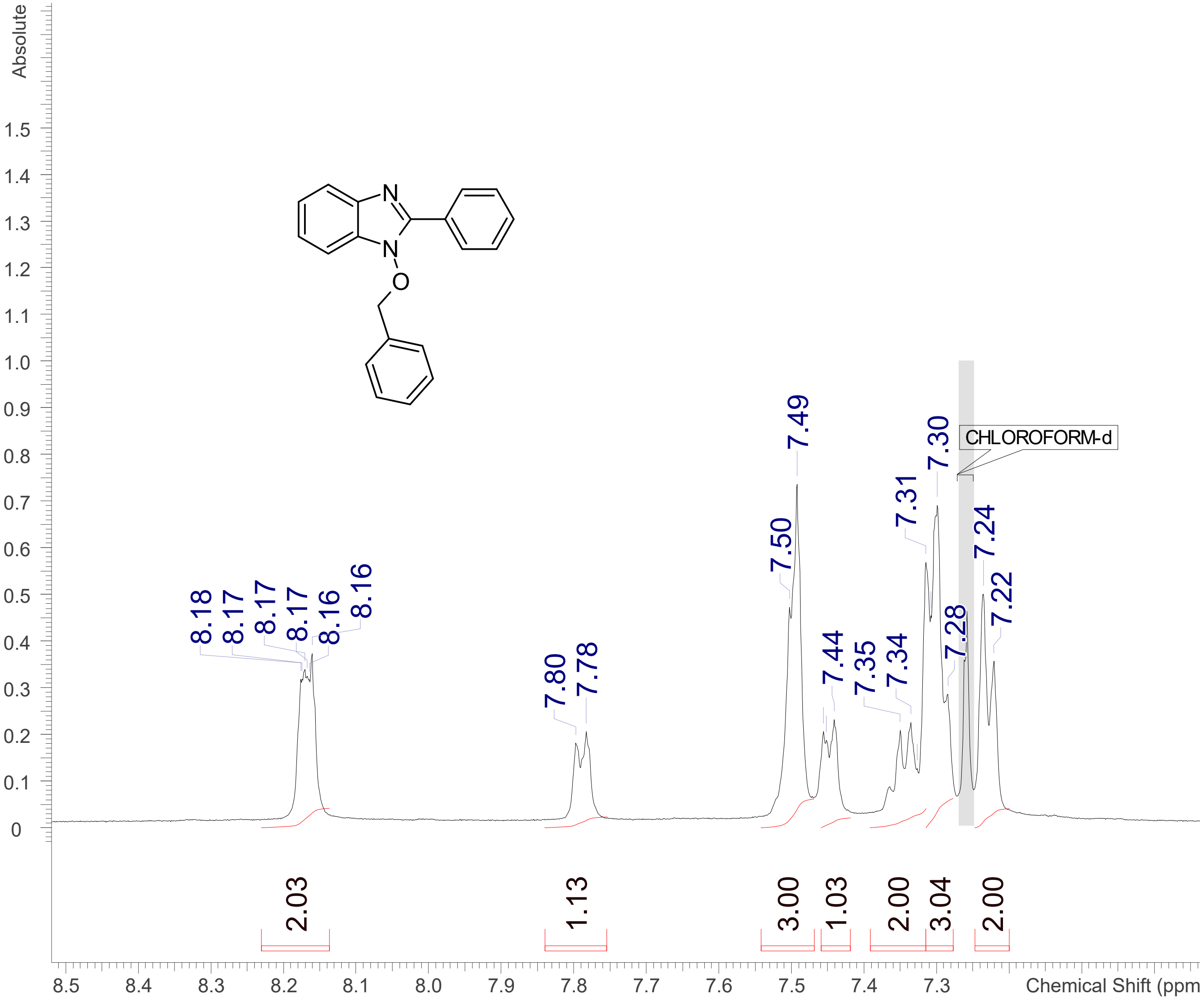

Figure 2S. Expanded region of the ^{1}H-NMR (Chloroform-*d*) spectrum for 1-(Benzyloxy)-2-phenyl-1*H*-benzo[*d*]imidazole **(1)** highlighting the aromatic proton signals.

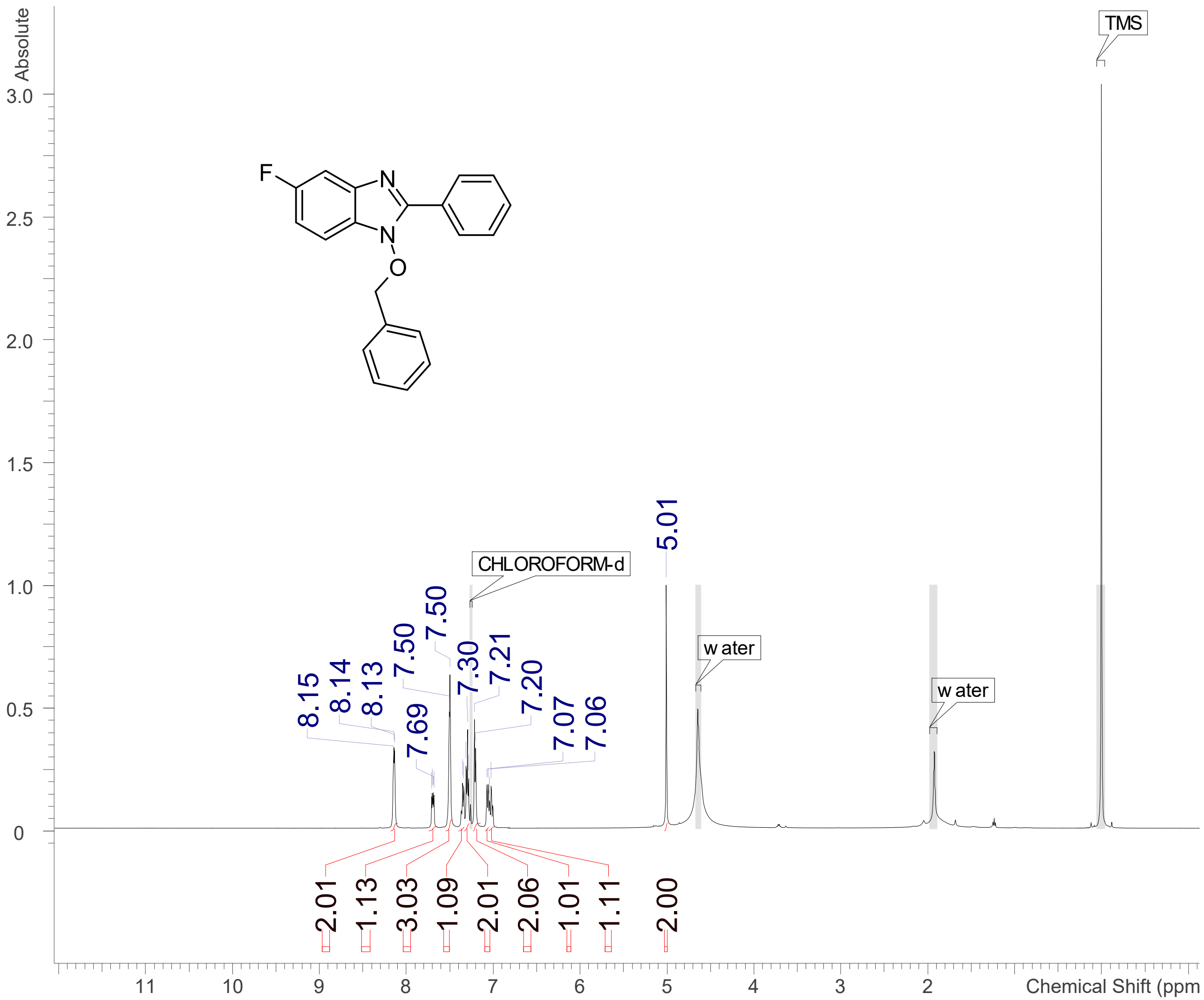

Figure 3S. ^{1}H NMR (500 MHz, CHLOROFORM-*d*) for 1-(Benzyloxy)-5-fluoro-2-phenyl-1*H*-benzo[*d*]imidazole **(2)**.

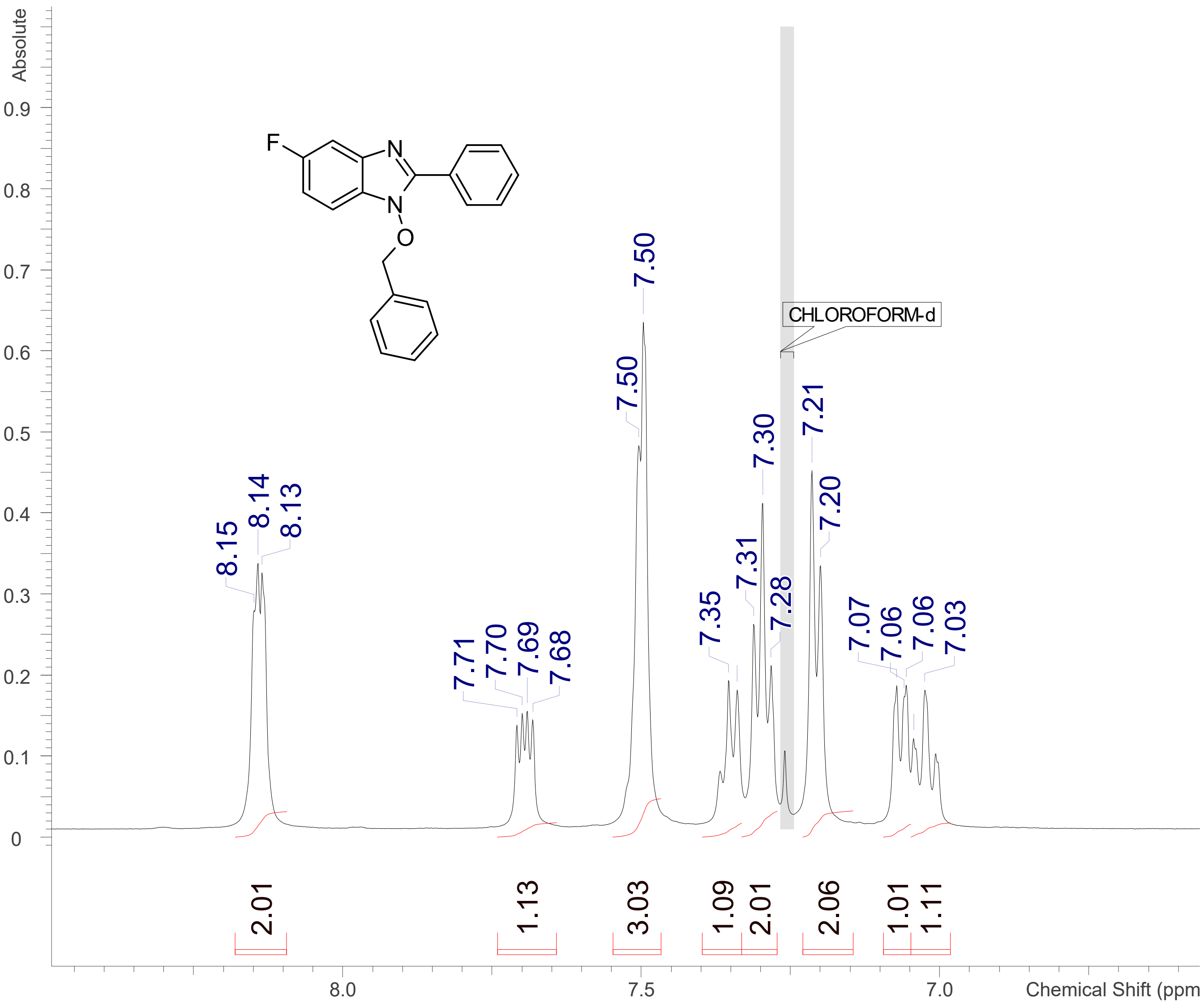

Figure 4S. Expanded region of the ^{1}H-NMR (Chloroform-*d*) spectrum for 1-(Benzyloxy)-5-fluoro-2-phenyl-1*H*-benzo[*d*]imidazole **(2)**

highlighting the aromatic proton signals.

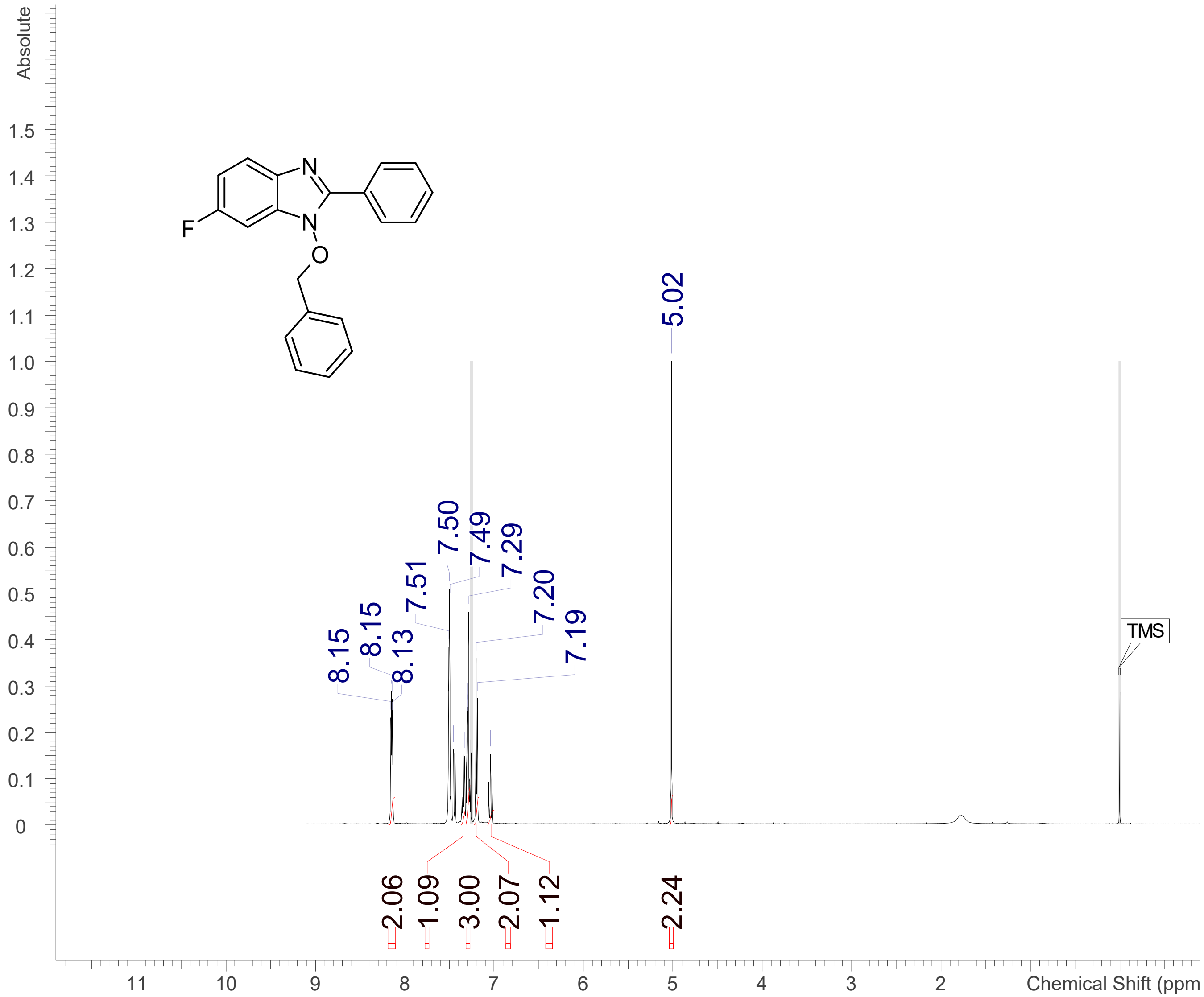

Figure 5S. ^{1}H NMR (500 MHz, CHLOROFORM-*d*) for 1-(Benzyloxy)-6-fluoro-2-phenyl-1*H*-benzo[*d*]imidazole **(3)**.

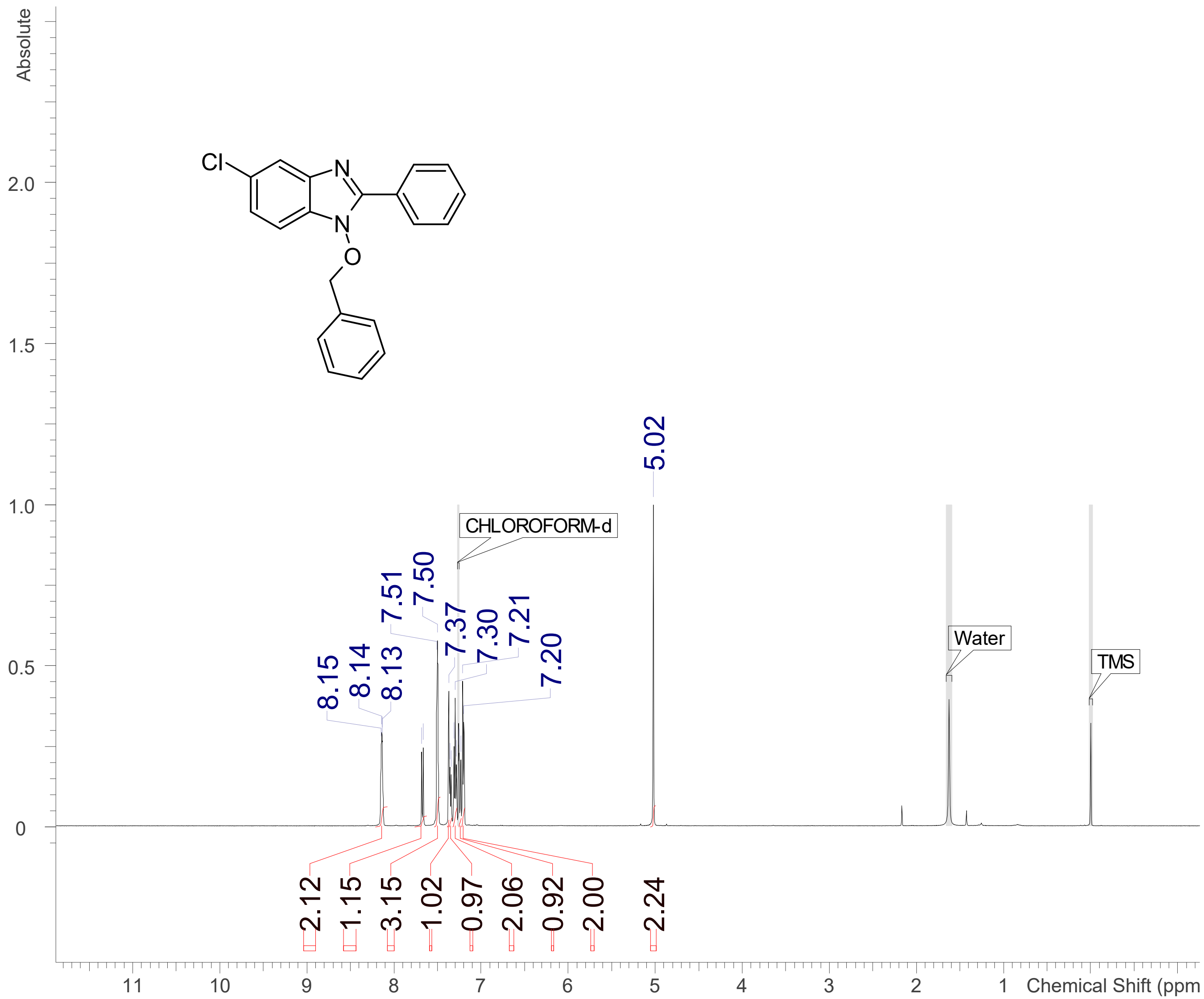

Figure 6S. ^{1}H NMR (500 MHz, CHLOROFORM-*d*) for 1-(Benzyloxy)-5-chloro-2-phenyl-1*H*-benzo[*d*]imidazole **(4)**.

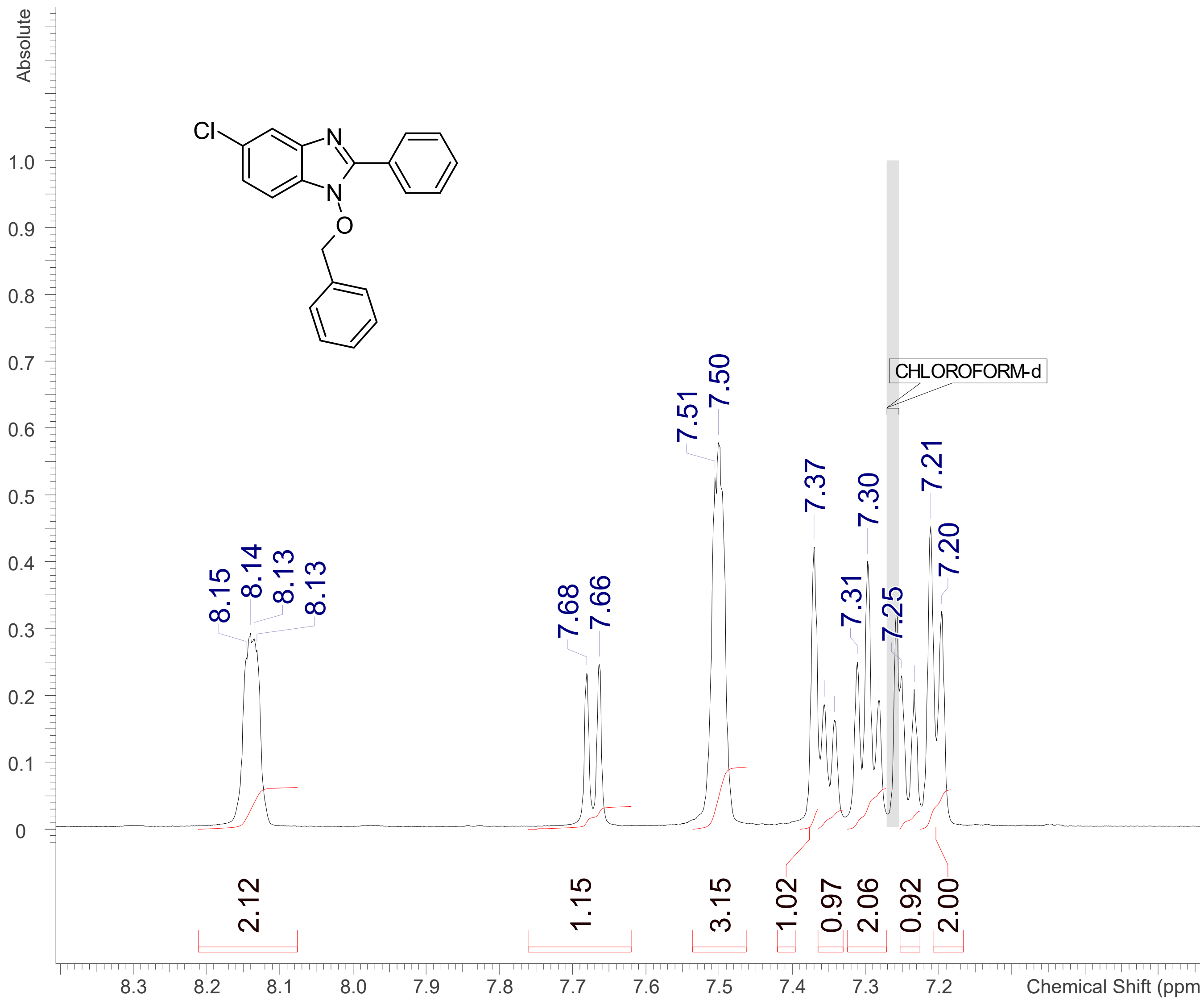

Figure 7S. Expanded region of the ^{1}H-NMR (Chloroform-*d*) spectrum for 1-(Benzyloxy)-5-chloro-2-phenyl-1*H*-benzo[*d*]imidazole **(4)** highlighting the aromatic proton signals.

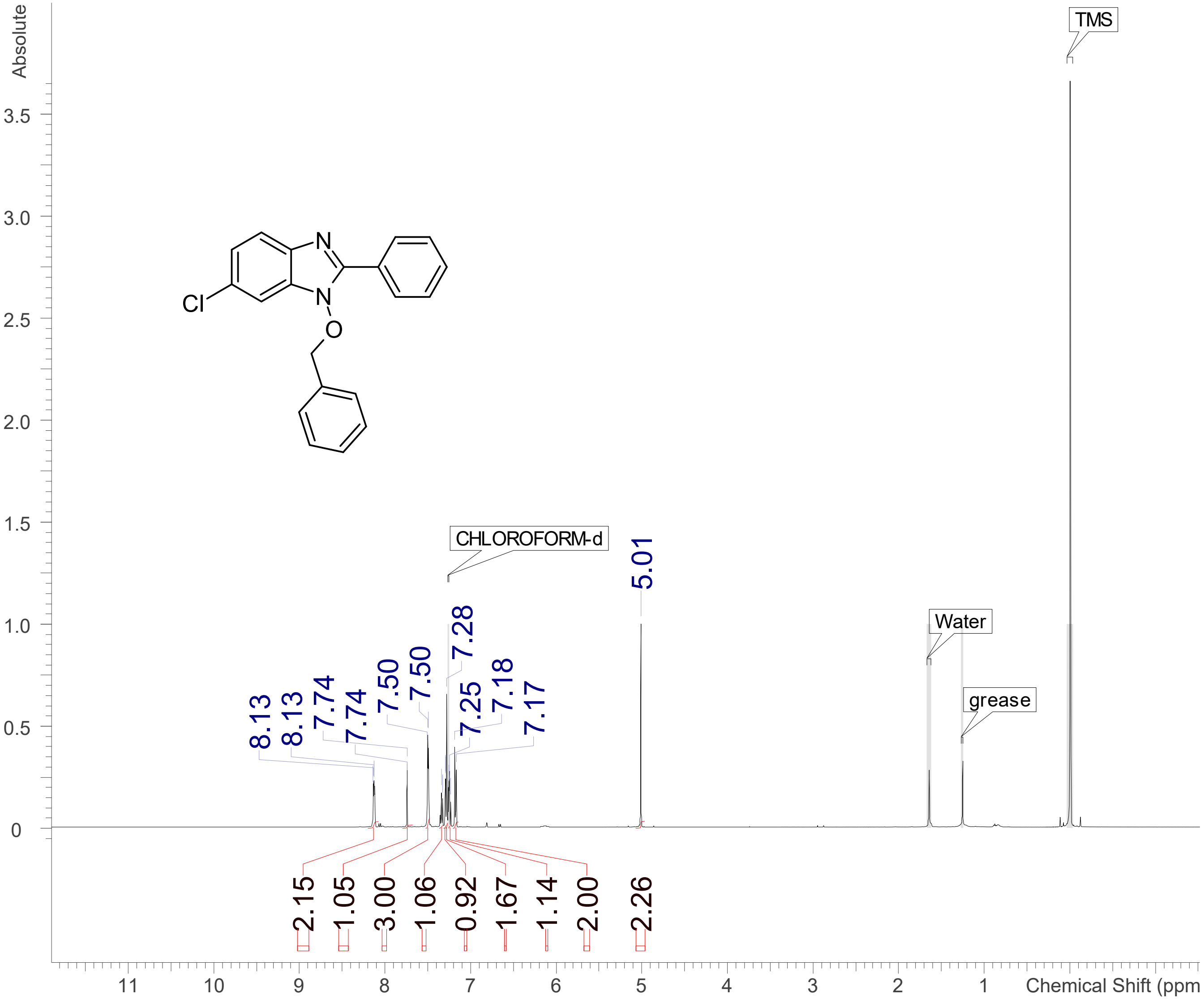

Figure 8S. ^{1}H NMR (500 MHz, CHLOROFORM-*d*) for 1-(Benzyloxy)-6-chloro-2-phenyl-1*H*-benzo[*d*]imidazole **(5)**.

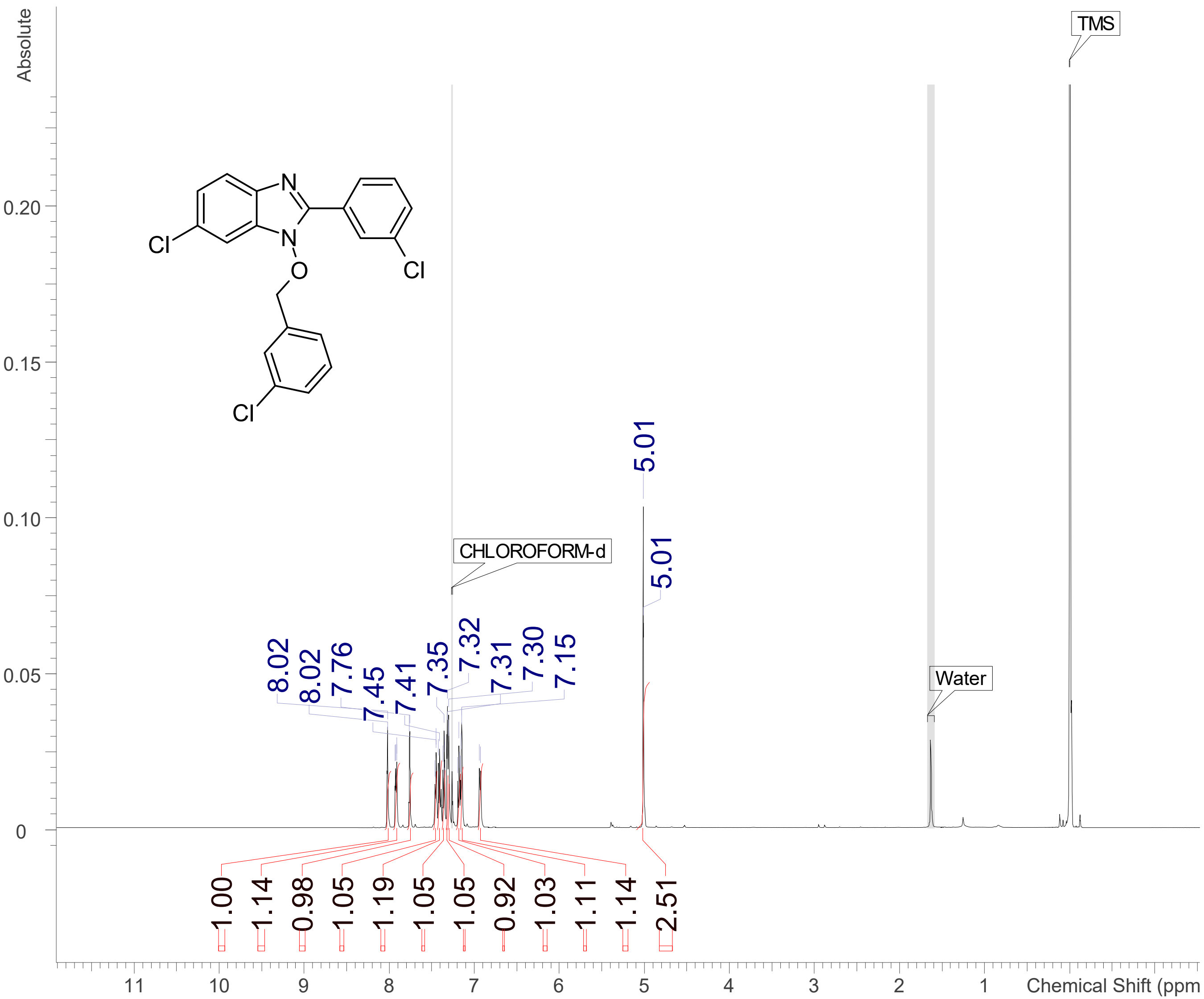

Figure 9S. ^{1}H NMR (500 MHz, CHLOROFORM-*d*) for 6-chloro-1-((3-chlorobenzyl)oxy)-2-(3-chlorophenyl)-1*H*-benzo[*d*]imidazole **(6)**.

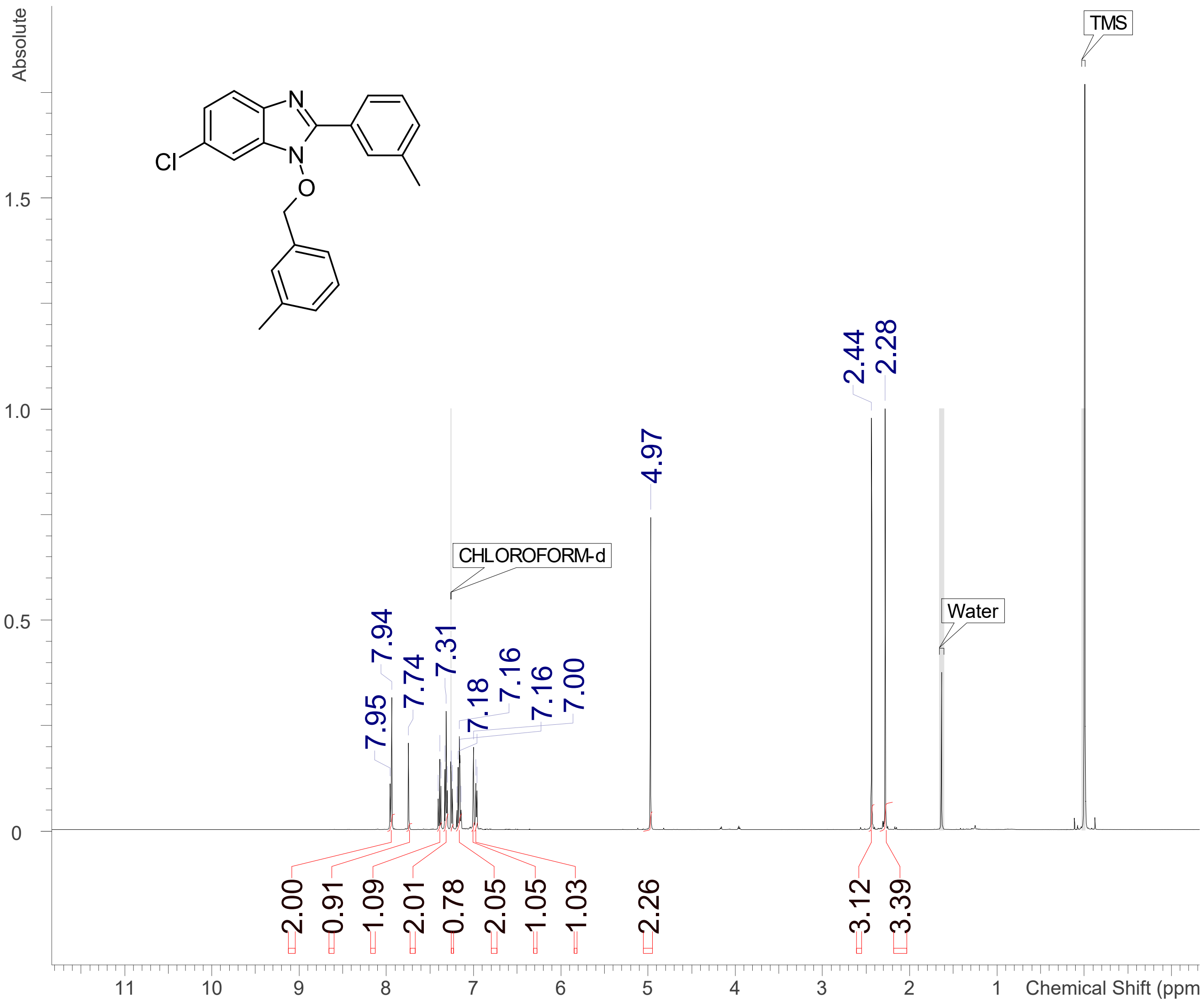

Figure 10S. ^{1}H NMR (500 MHz, CHLOROFORM-*d*) for 6-Chloro-1-((3-methylbenzyl)oxy)-2-(m-tolyl)-1*H*-benzo[*d*]imidazole **(7)**.

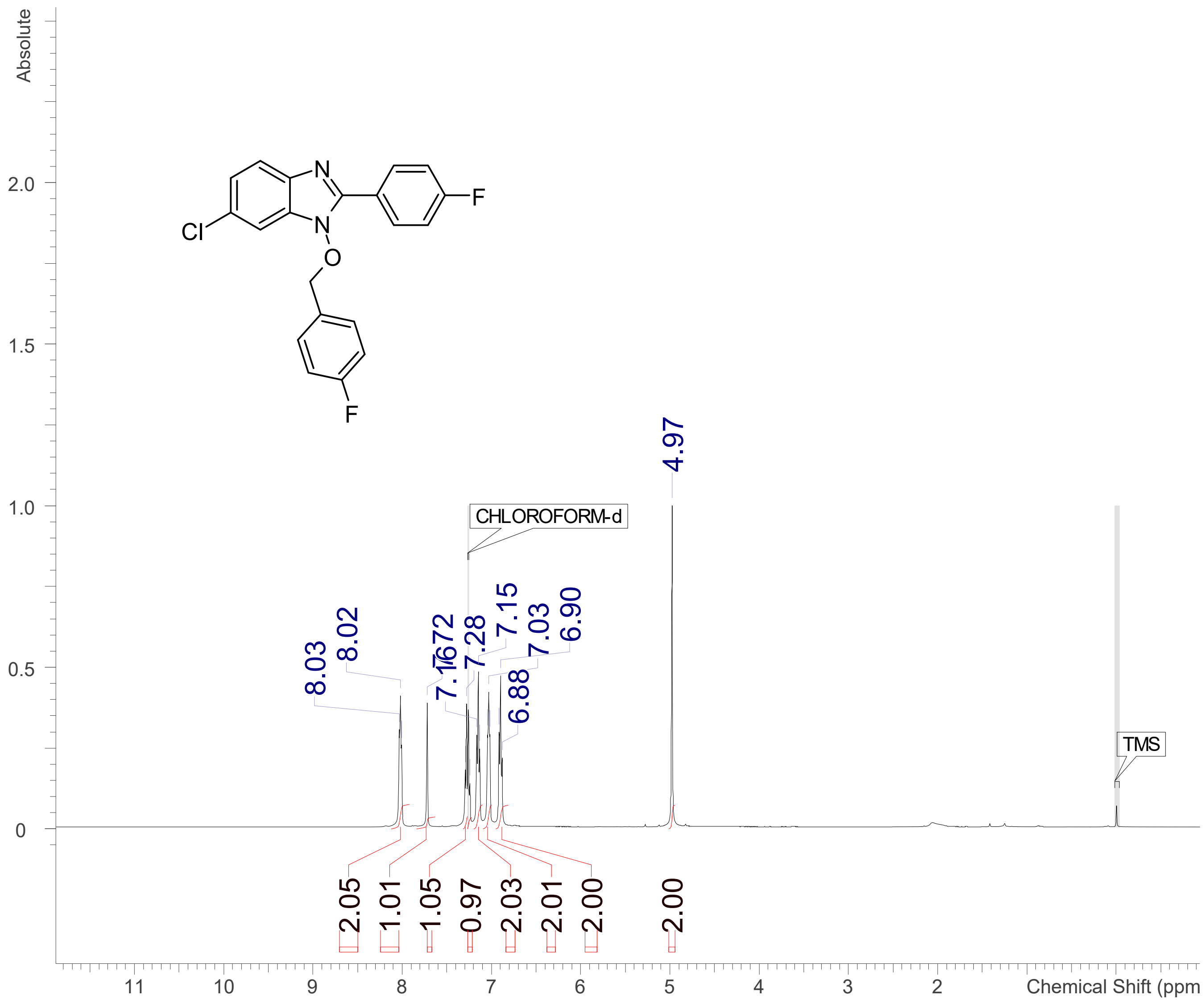

Figure 11S. ^{1}H NMR (500 MHz, CHLOROFORM-*d*) for 6-Chloro-1-((4-fluorobenzyl)oxy)-2-(4-fluorophenyl)-1*H*-benzo[*d*]imidazole **(8)**.

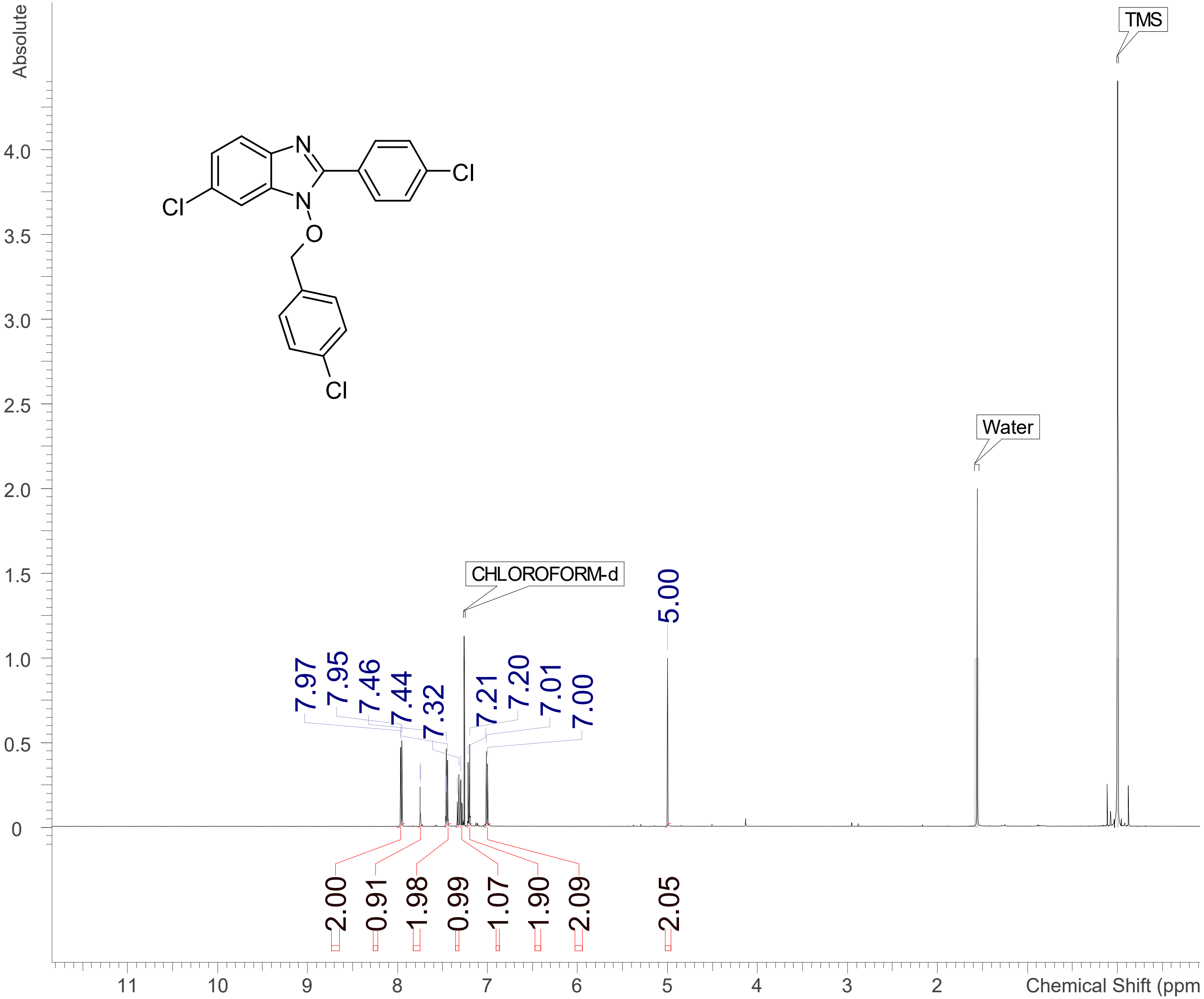

Figure 12S. ^{1}H NMR (500 MHz, CHLOROFORM-*d*) for 6-Chloro-1-((4-chlorobenzyl)oxy)-2-(4-chlorophenyl)-1*H*-benzo[*d*]imidazole **(9)**.

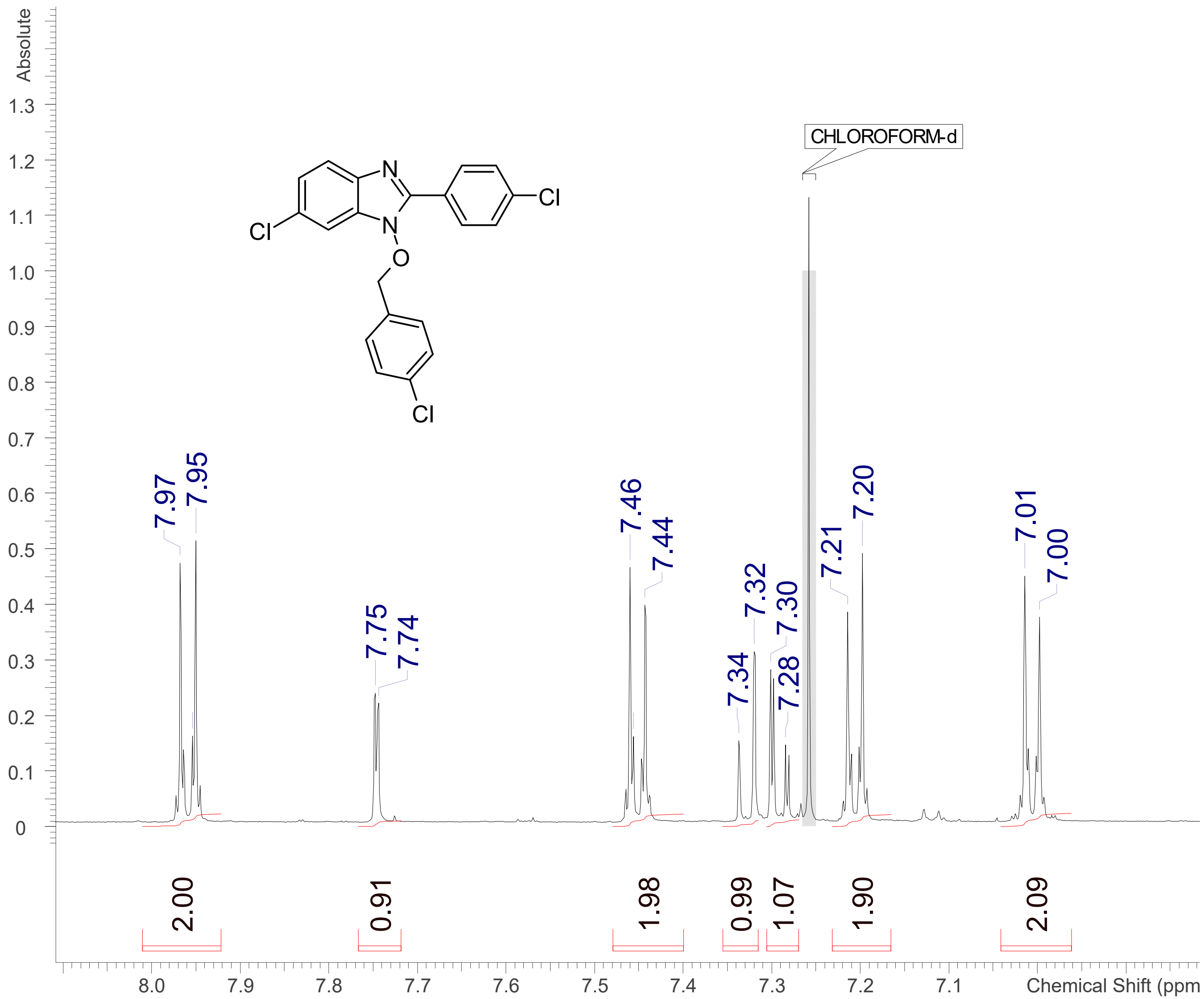

Figure 13S. Expanded region of the ^{1}H-NMR (Chloroform-*d*) spectrum for 6-Chloro-1-((4-chlorobenzyl)oxy)-2-(4-chlorophenyl)-1*H*-benzo[*d*]imidazole **(9)** highlighting the aromatic proton signals.

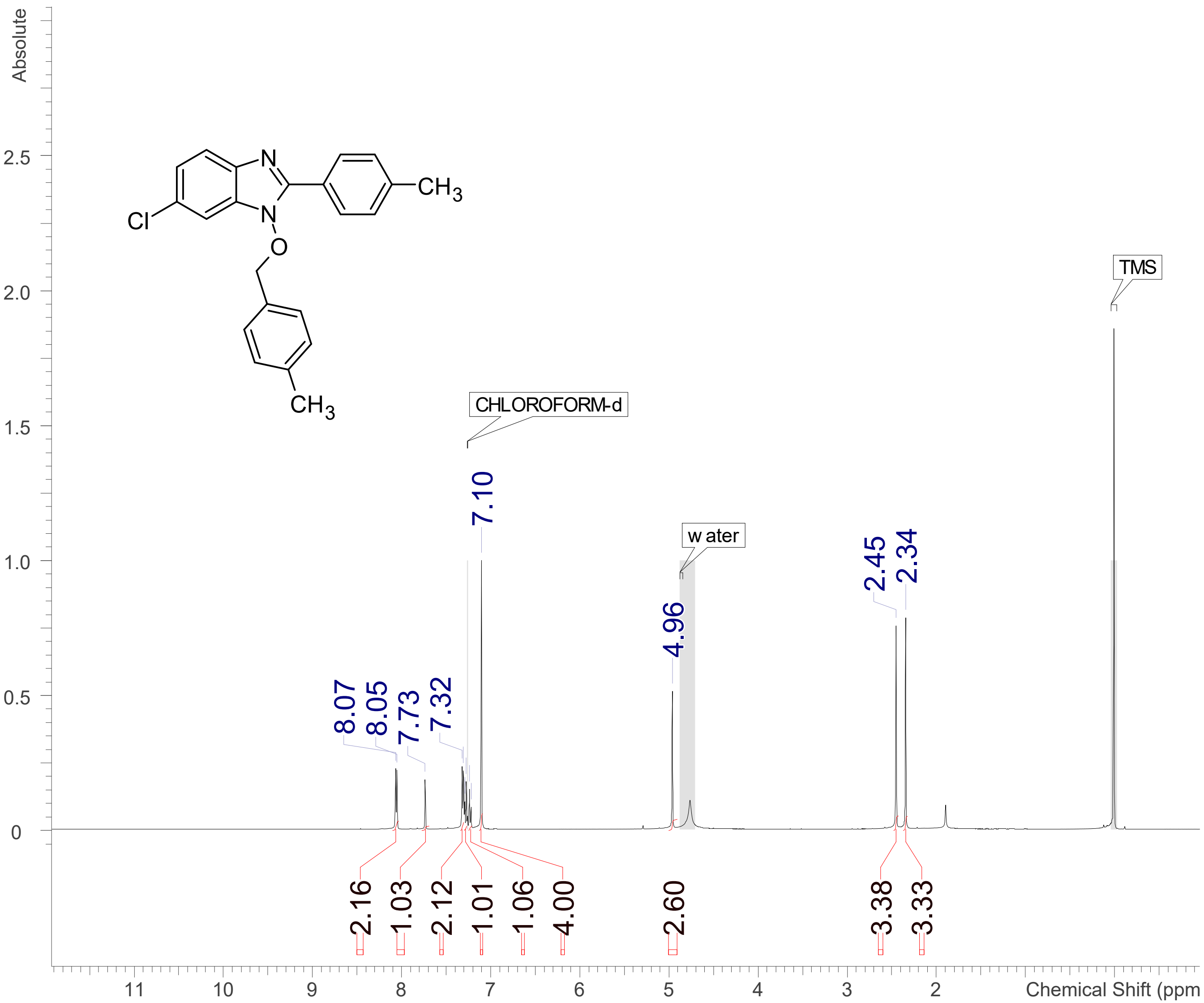

Figure 14S. ^{1}H NMR (500 MHz, CHLOROFORM-*d*) for 6-Chloro-1-((4-methylbenzyl)oxy)-2-(p-tolyl)-1*H*-benzo[*d*]imidazole **(10)**.

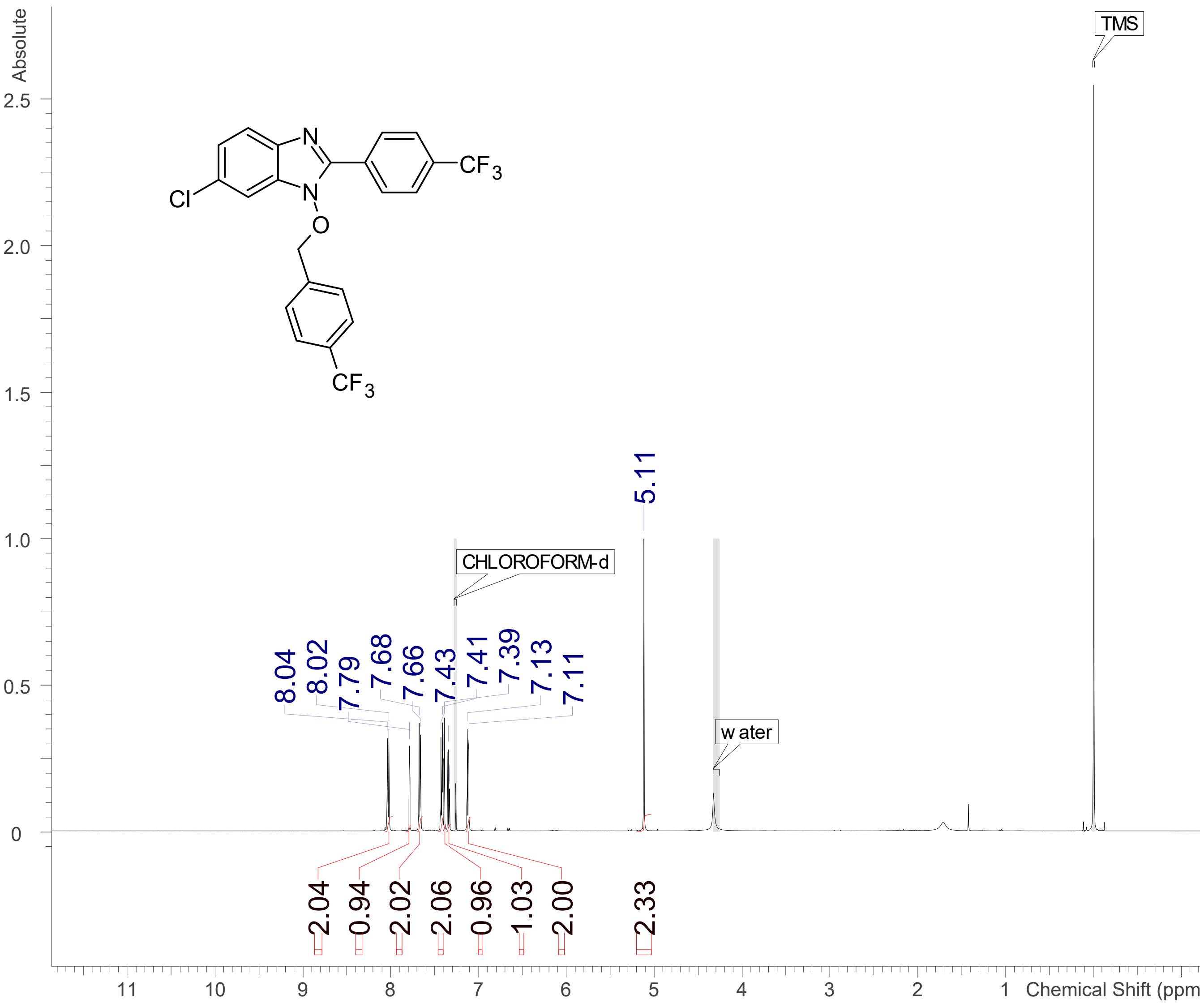

Figure 15S. ^{1}H NMR (500 MHz, CHLOROFORM-*d*) for 6-Chloro-1-((4-(trifluoromethyl)benzyl)oxy)-2-(4-(trifluoromethyl)phenyl)-1*H*-benzo[*d*]imidazole **(11)**.

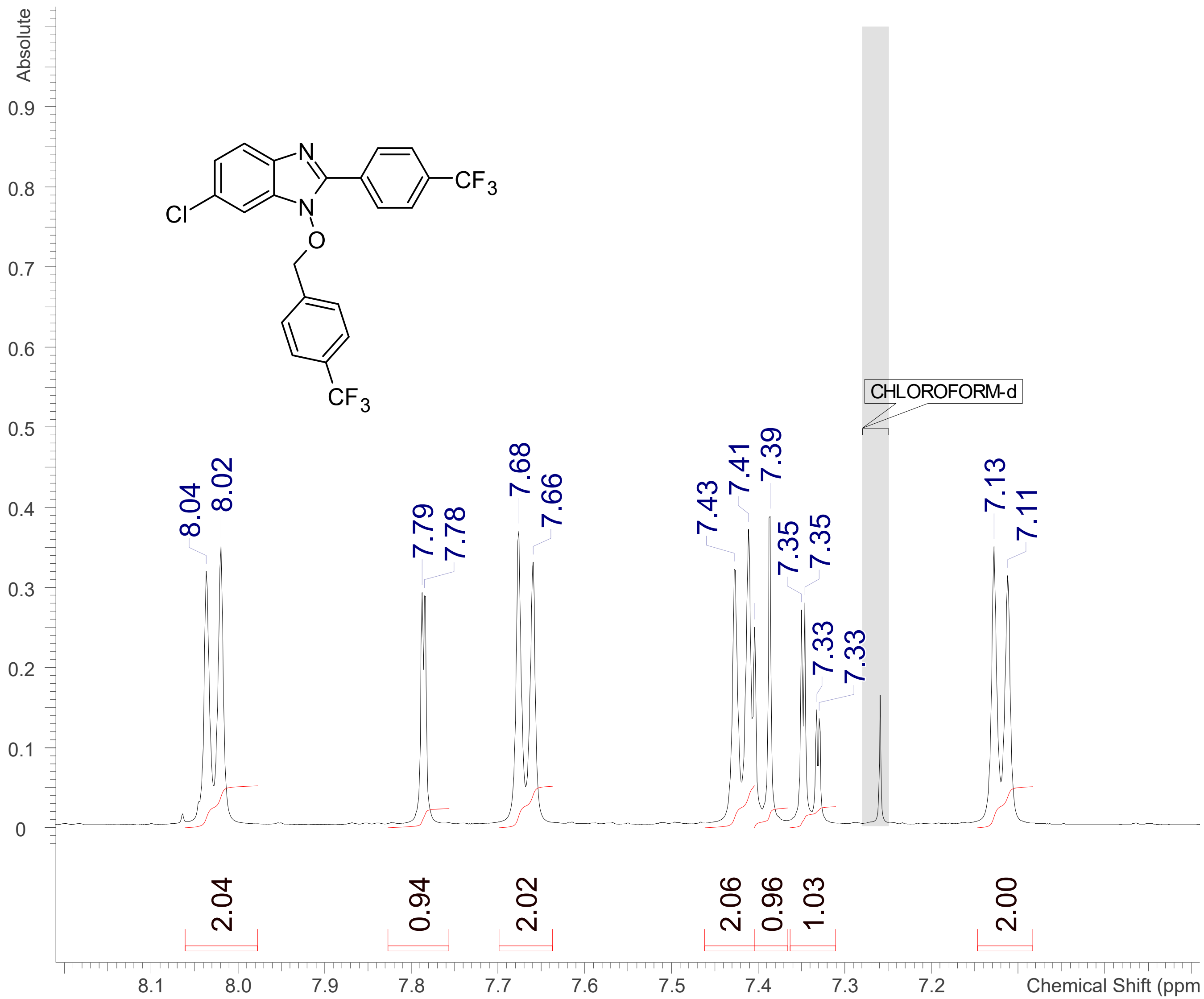

Figure 16S. Expanded region of the ^{1}H-NMR (Chloroform-*d*) spectrum 6-Chloro-1-((4-(trifluoromethyl)benzyl)oxy)-2-(4-(trifluoromethyl)phenyl)-1*H*-benzo[*d*]imidazole **(11)** highlighting the aromatic proton signals.

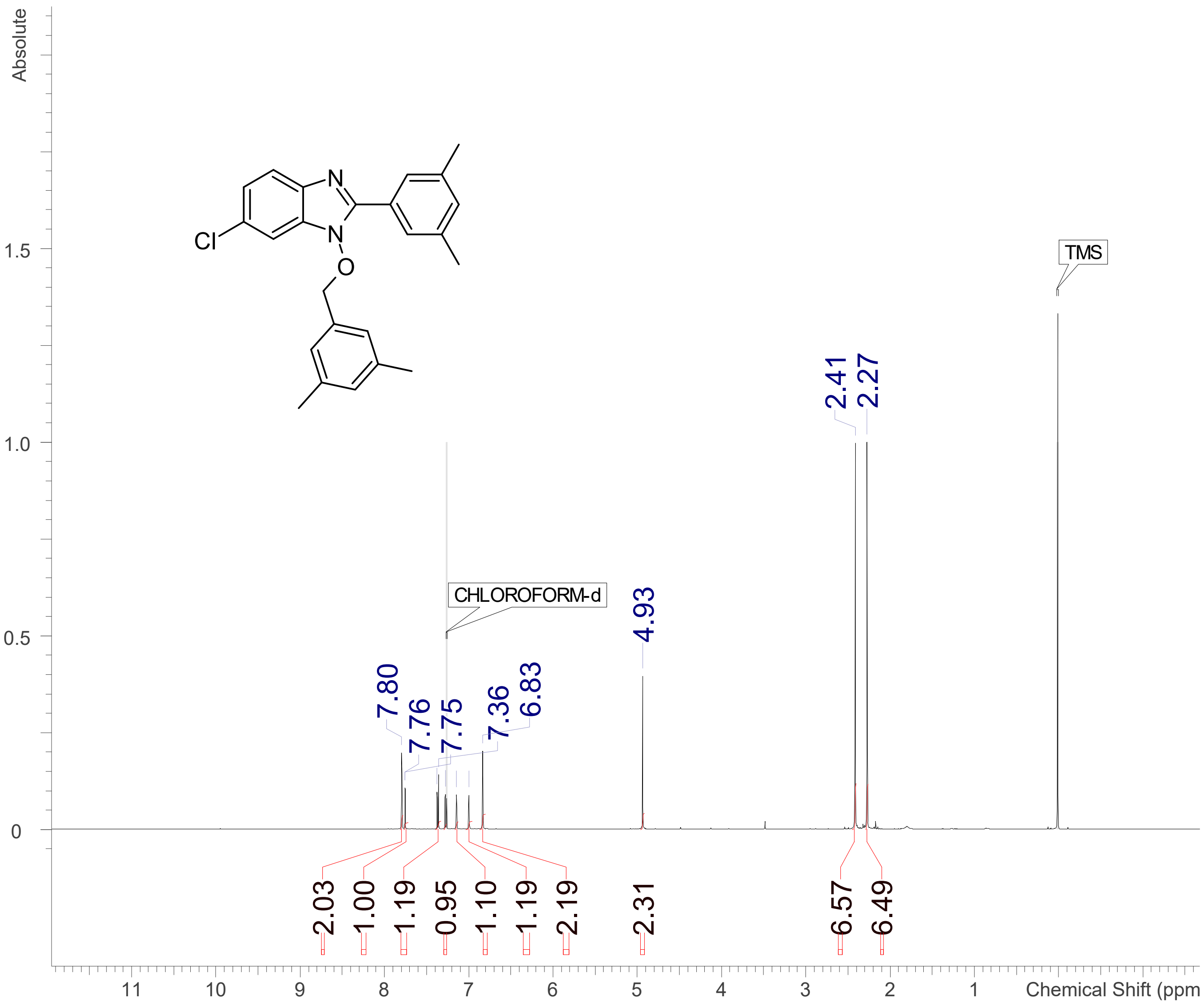

Figure 17S. ^{1}H NMR (500 MHz, CHLOROFORM-*d*) for 6-Chloro-1-((3,5-dimethylbenzyl)oxy)-2-(3,5-dimethylphenyl)-1*H*-benzo[*d*]imidazole **(12)**.

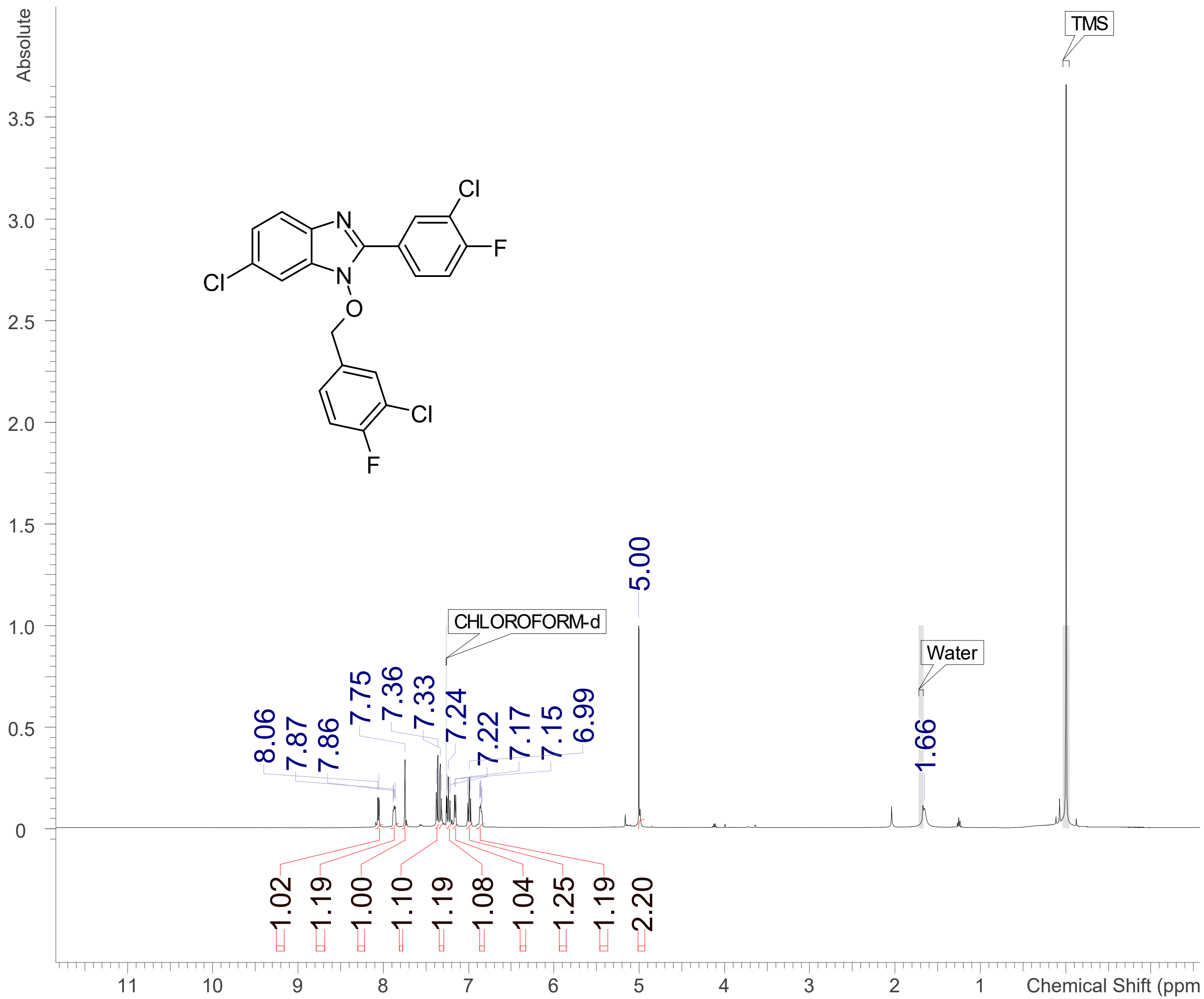

Figure 18S. ^{1}H NMR (500 MHz, CHLOROFORM-*d*) for 6-Chloro-1-((3-chloro-4-fluorobenzyl)oxy)-2-(3-chloro-4-fluorophenyl)-1*H*-benzo[*d*]imidazole **(13)**.

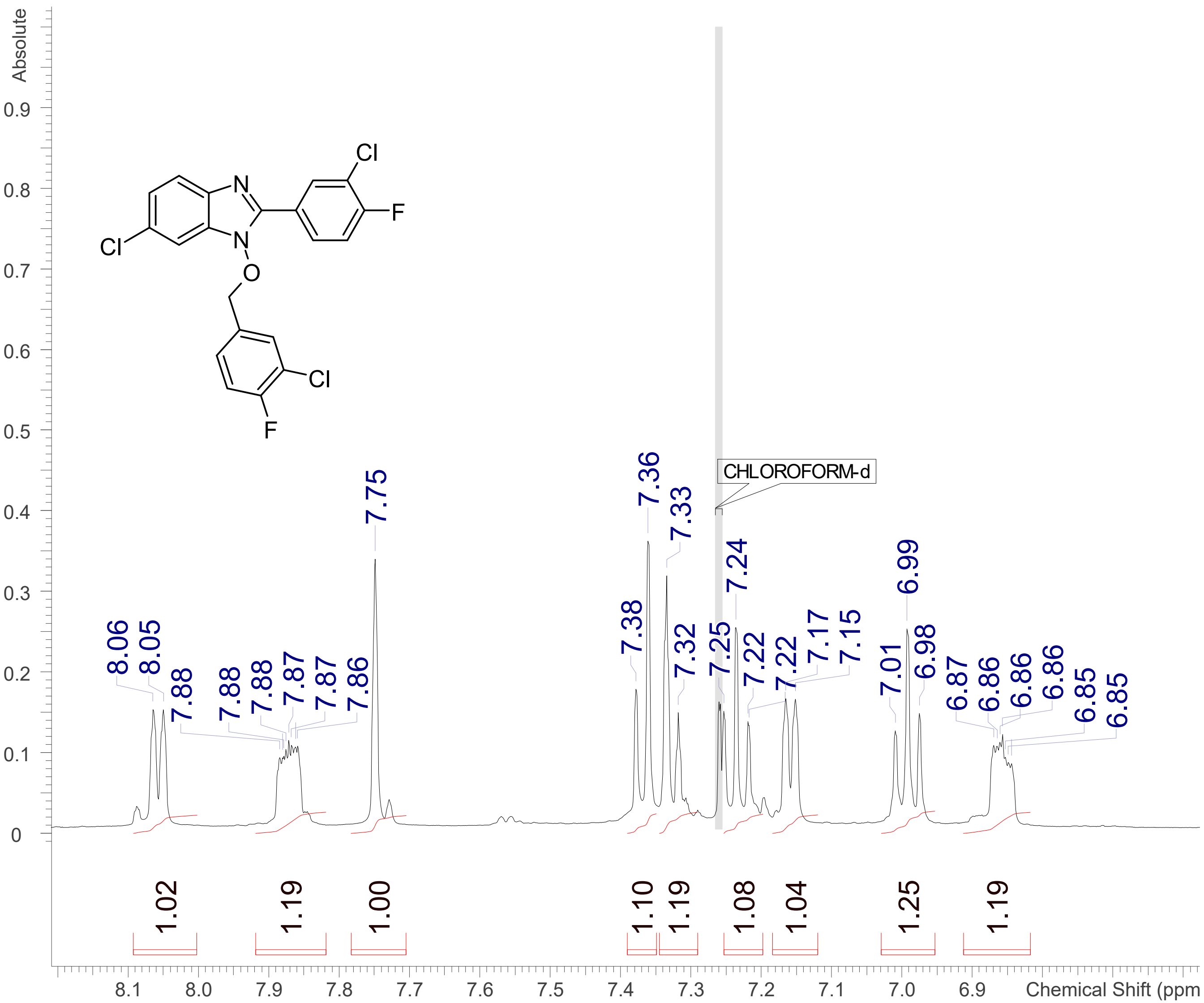

Figure 19S. Expanded region of the ^{1}H-NMR (Chloroform-*d*) spectrum for for 6-Chloro-1-((3-chloro-4-fluorobenzyl)oxy)-2-(3-chloro-4-fluorophenyl)-1*H*-benzo[*d*]imidazole **(13)** highlighting the aromatic proton signals.

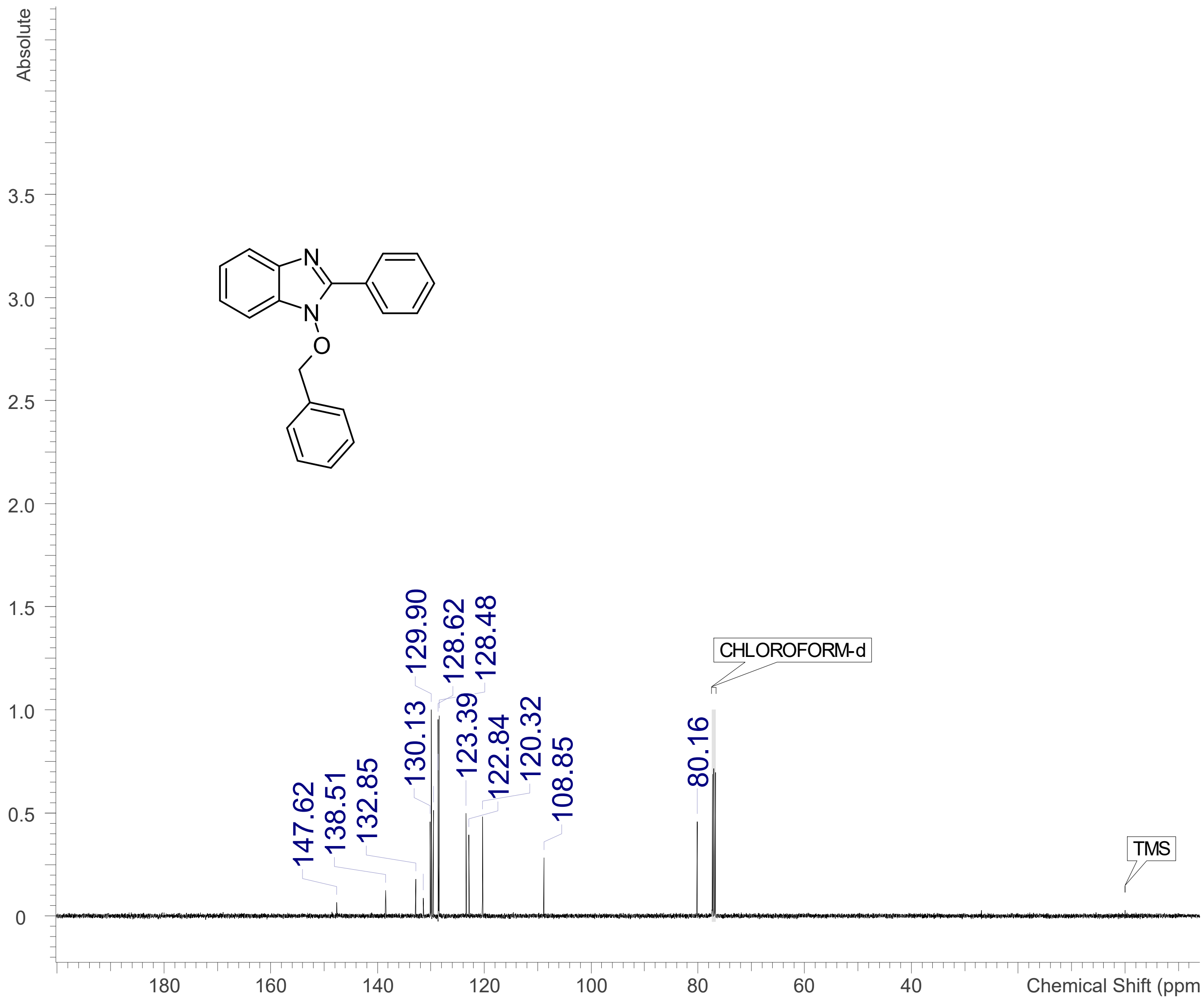

Figure 20S. ^{13}C NMR (126 MHz, CHLOROFORM-*d*) for 1-(Benzyloxy)-2-phenyl-1*H*-benzo[*d*]imidazole **(1)**.

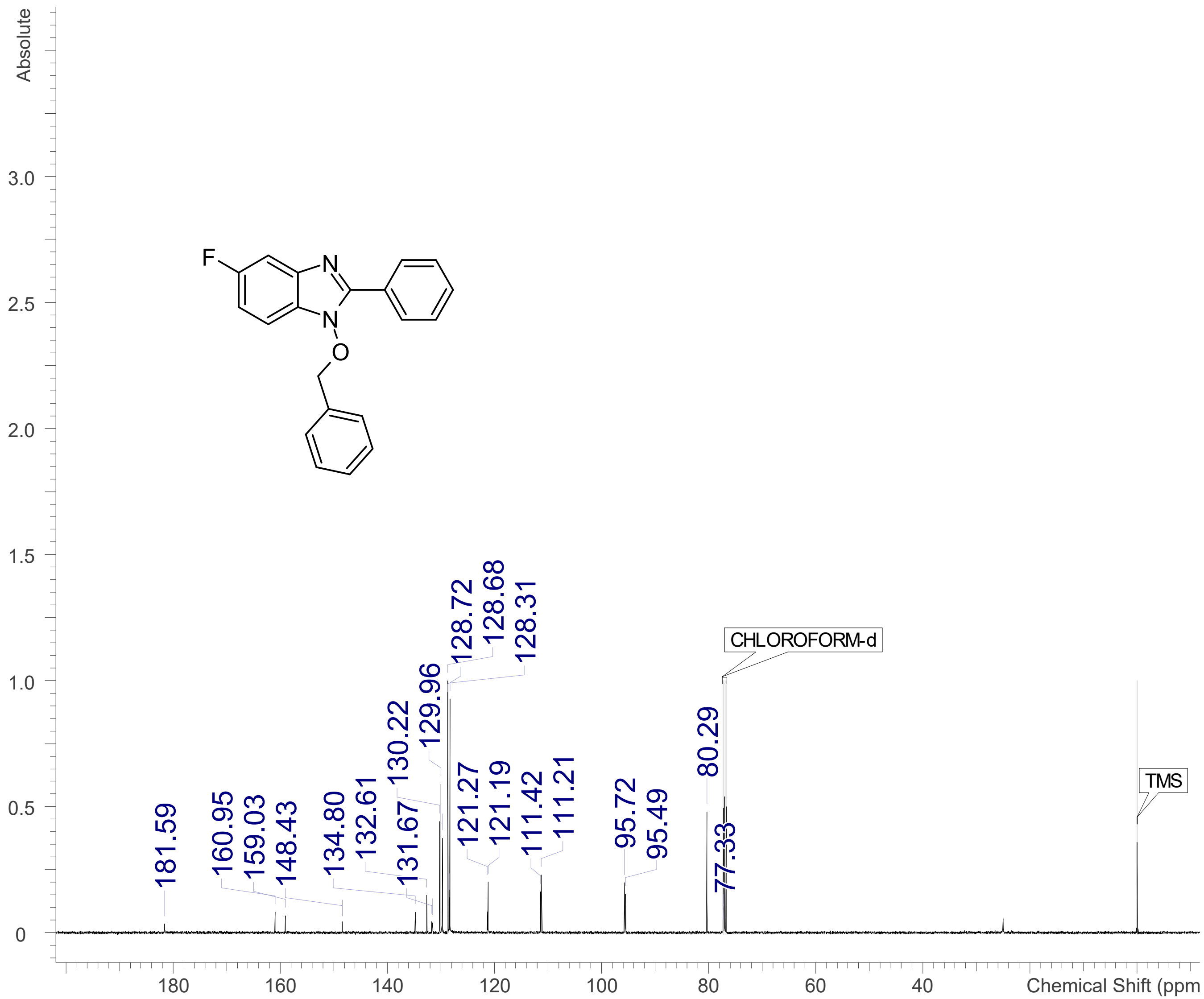

Figure 21S. ^{13}C NMR (126 MHz, CHLOROFORM-*d*) for 1-(Benzyloxy)-5-fluoro-2-phenyl-1*H*-benzo[*d*]imidazole **(2)**.

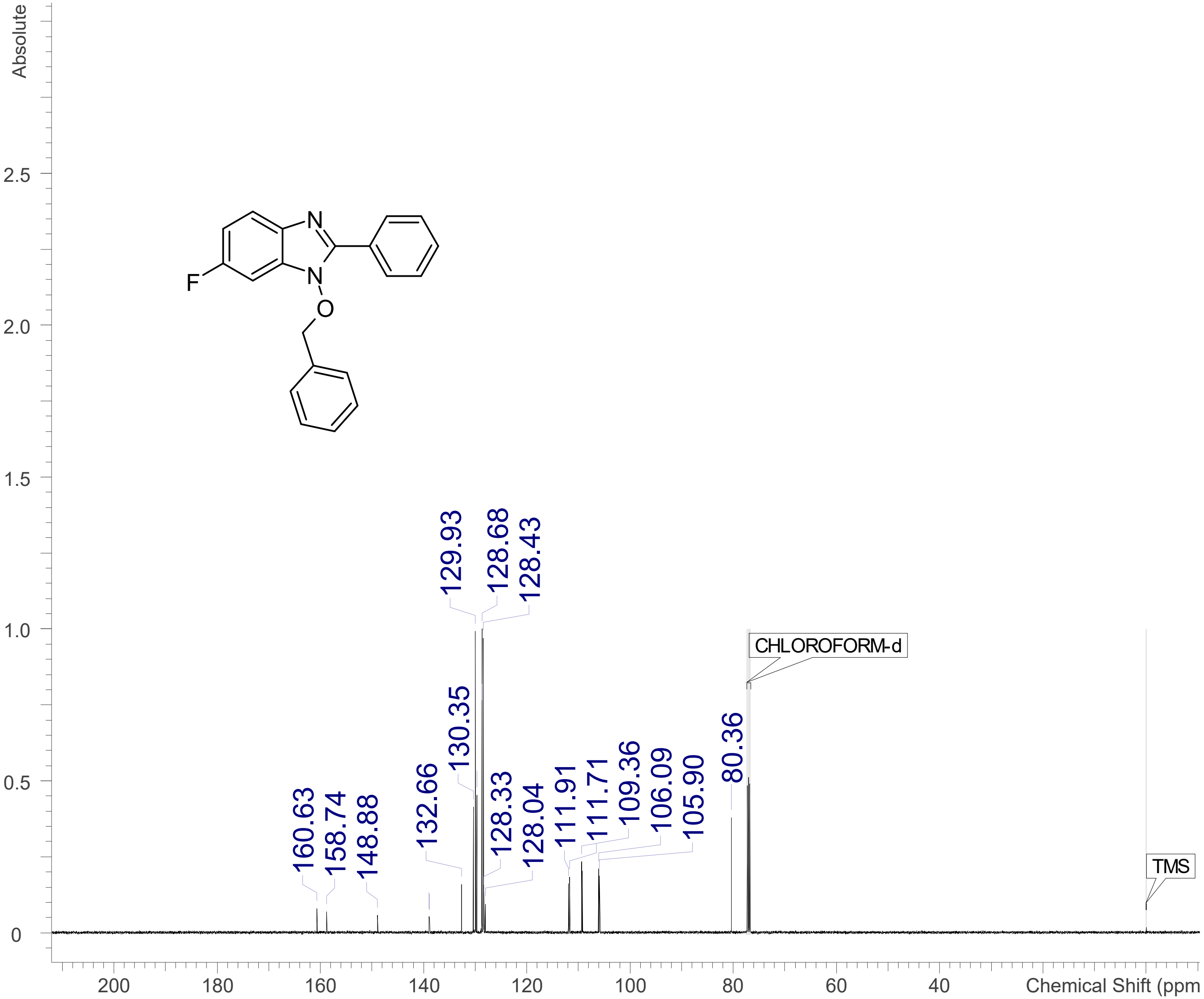

Figure 22S. ^{13}C NMR (126 MHz, CHLOROFORM-*d*) for 1-(Benzyloxy)-6-fluoro-2-phenyl-1*H*-benzo[*d*]imidazole **(3)**.

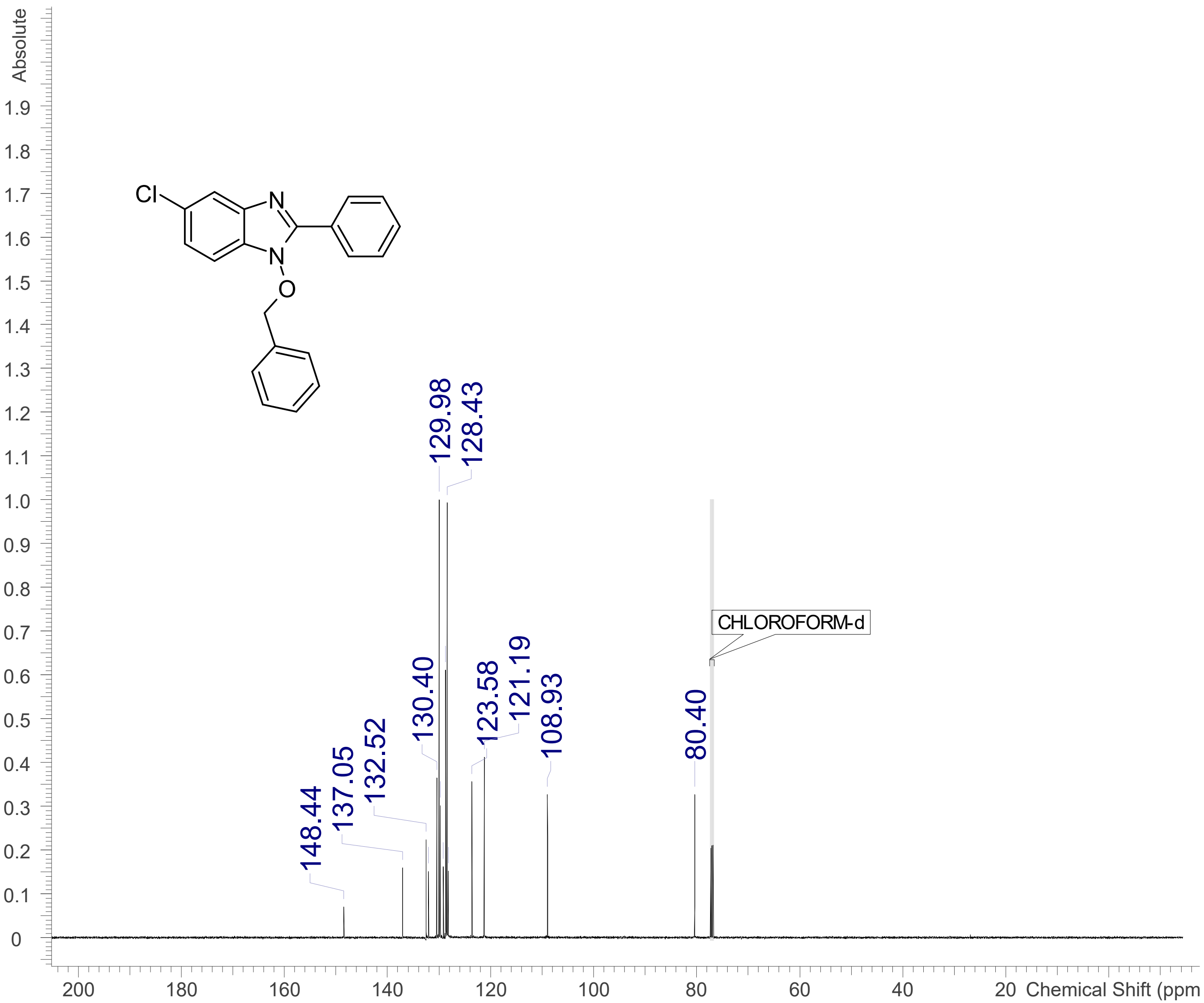

Figure 23S. ^{13}C NMR (126 MHz, CHLOROFORM-*d*) for 1-(Benzyloxy)-5-chloro-2-phenyl-1*H*-benzo[*d*]imidazole **(4)**.

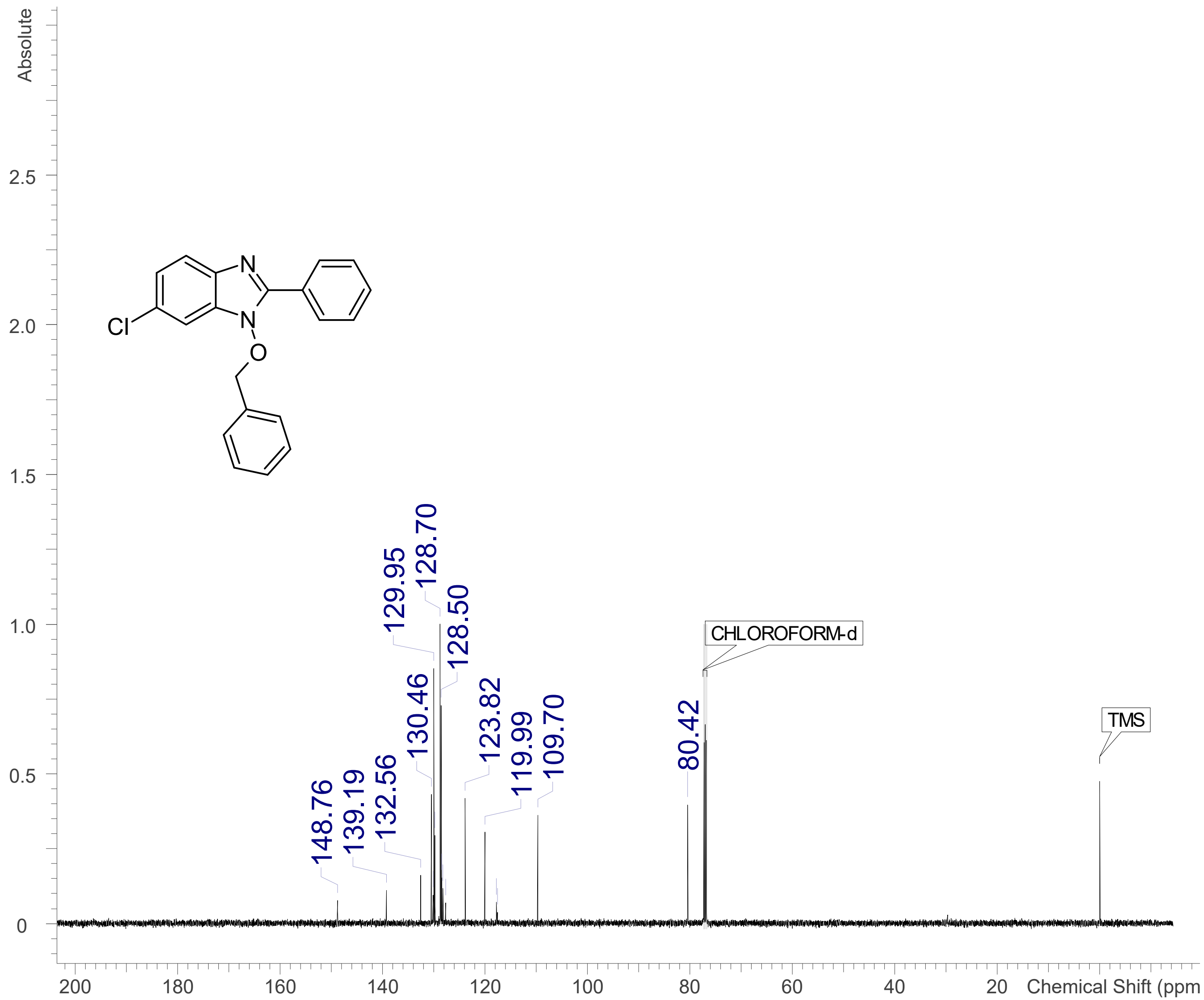

Figure 24S. ^{13}C NMR (126 MHz, CHLOROFORM-*d*) for 1-(Benzyloxy)-6-chloro-2-phenyl-1*H*-benzo[*d*]imidazole **(5)**.

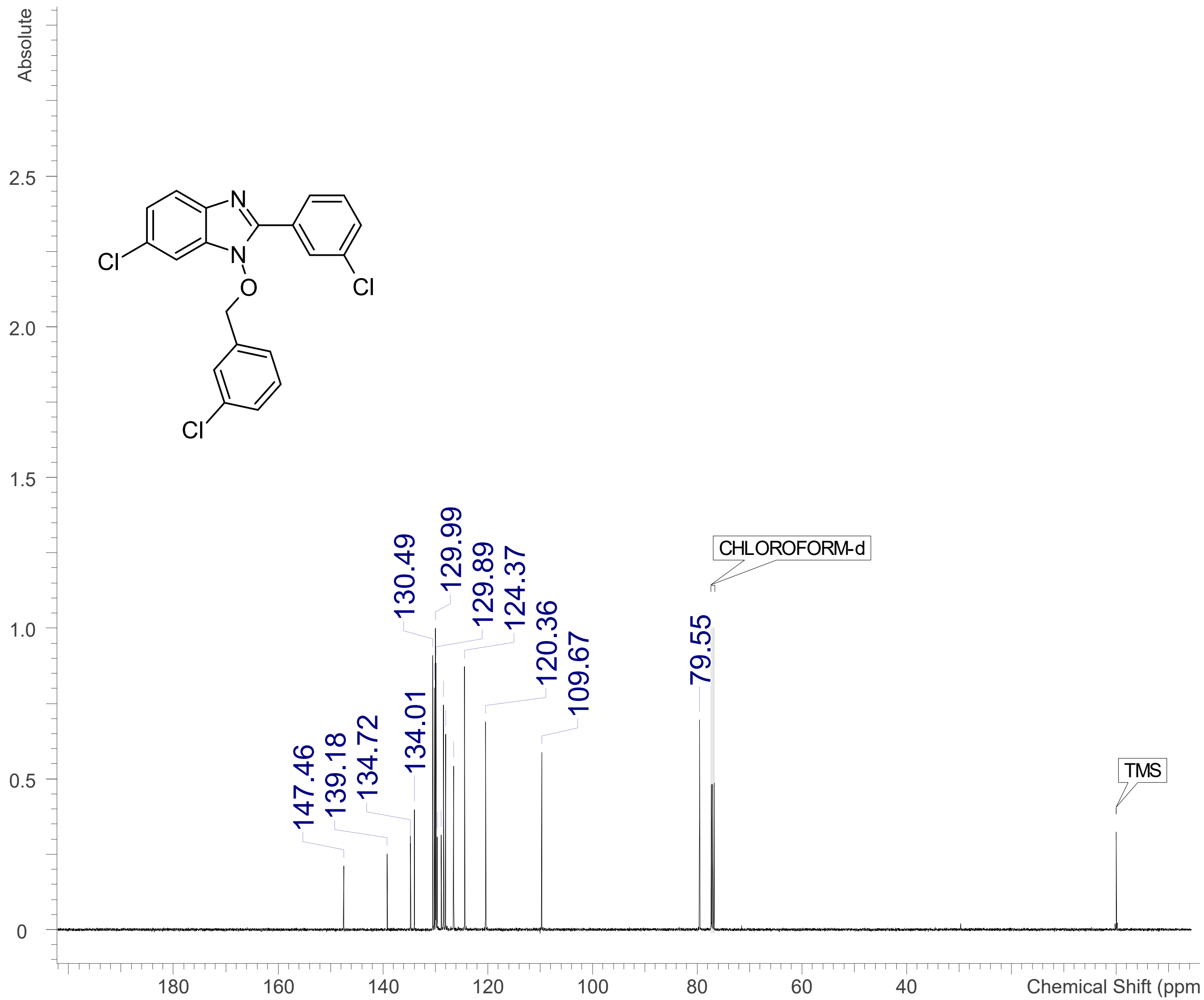

Figure 25S. ^{13}C NMR (126 MHz, CHLOROFORM-*d*) for 6-Chloro-1-((3-chlorobenzyl)oxy)-2-(3-chlorophenyl)-1*H*-benzo[*d*]imidazole **(6)**.

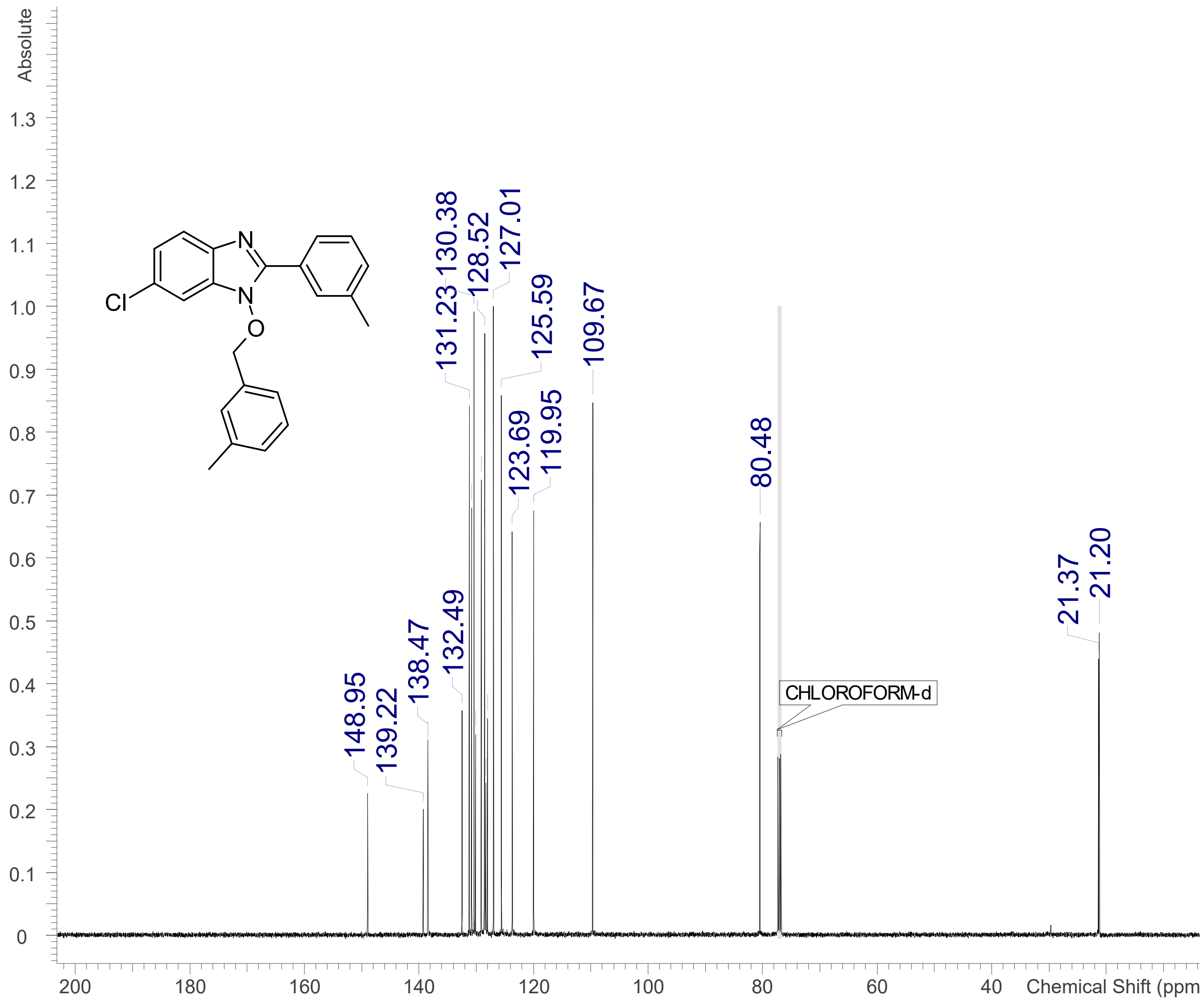

Figure 26S. ^{13}C NMR (126 MHz, CHLOROFORM-*d*) for 6-Chloro-1-((3-methylbenzyl)oxy)-2-(m-tolyl)-1*H*-benzo[*d*]imidazole **(7)**.

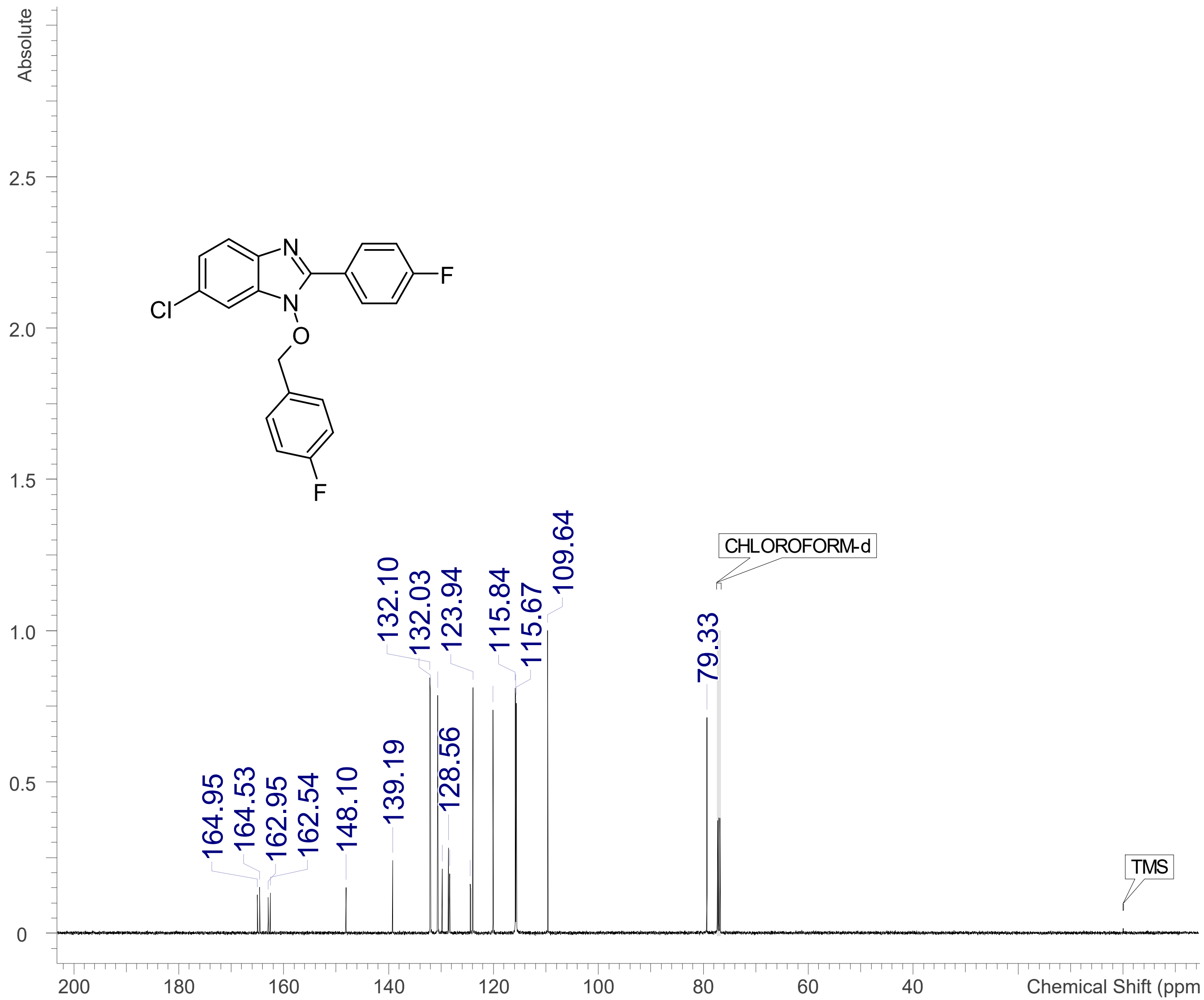

Figure 27S. ^{13}C NMR (126 MHz, CHLOROFORM-*d*) for 6-Chloro-1-((4-fluorobenzyl)oxy)-2-(4-fluorophenyl)-1*H*-benzo[*d*]imidazole **(8)**.

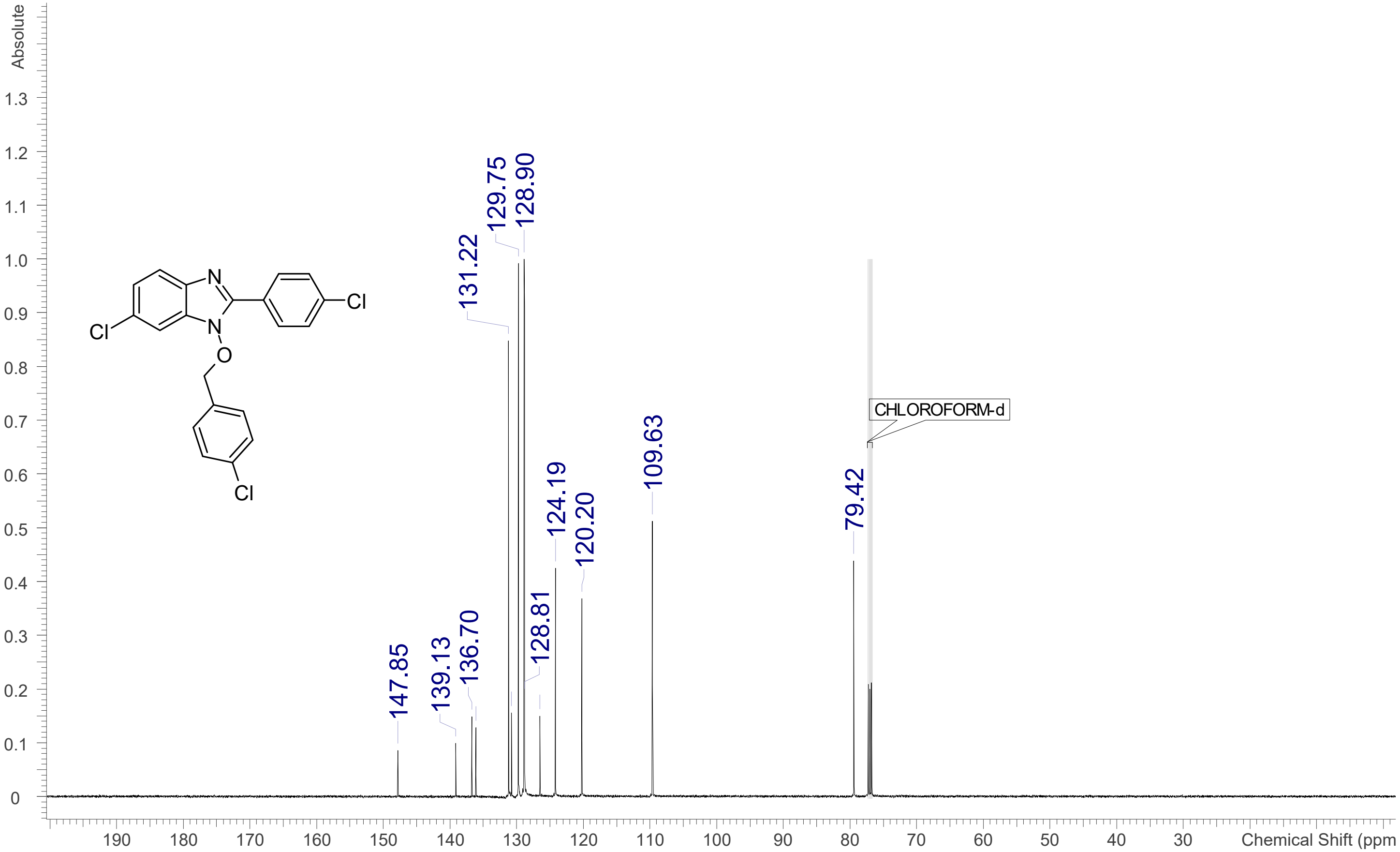

Figure 28S. ^{13}C NMR (126 MHz, CHLOROFORM-*d*) for 6-Chloro-1-((4-chlorobenzyl)oxy)-2-(4-chlorophenyl)-1*H*-benzo[*d*]imidazole **(9)**.

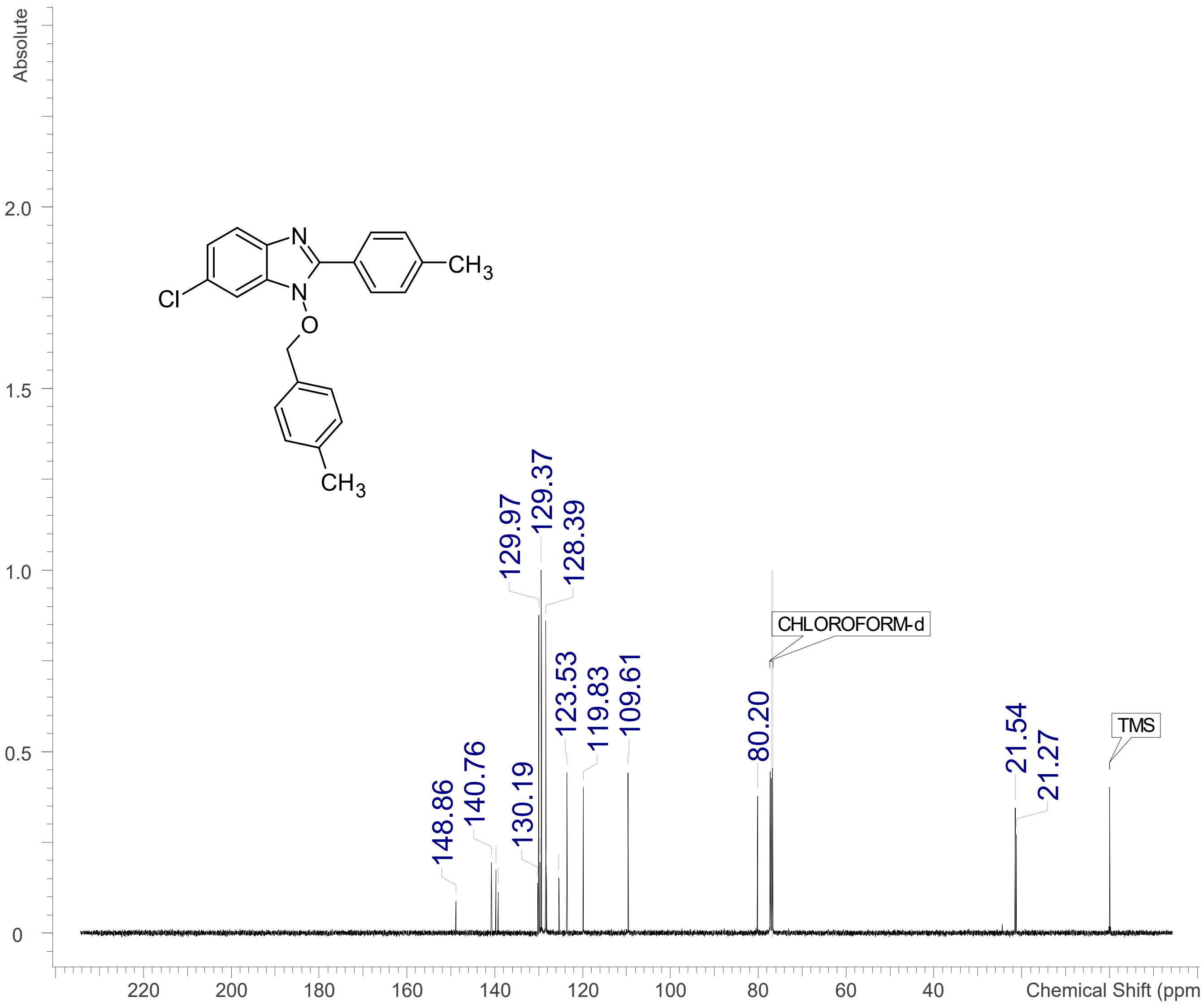

Figure 29S. ^{13}C NMR (126 MHz, CHLOROFORM-*d*) for 6-Chloro-1-((4-methylbenzyl)oxy)-2-(p-tolyl)-1*H*-benzo[*d*]imidazole **(10).**

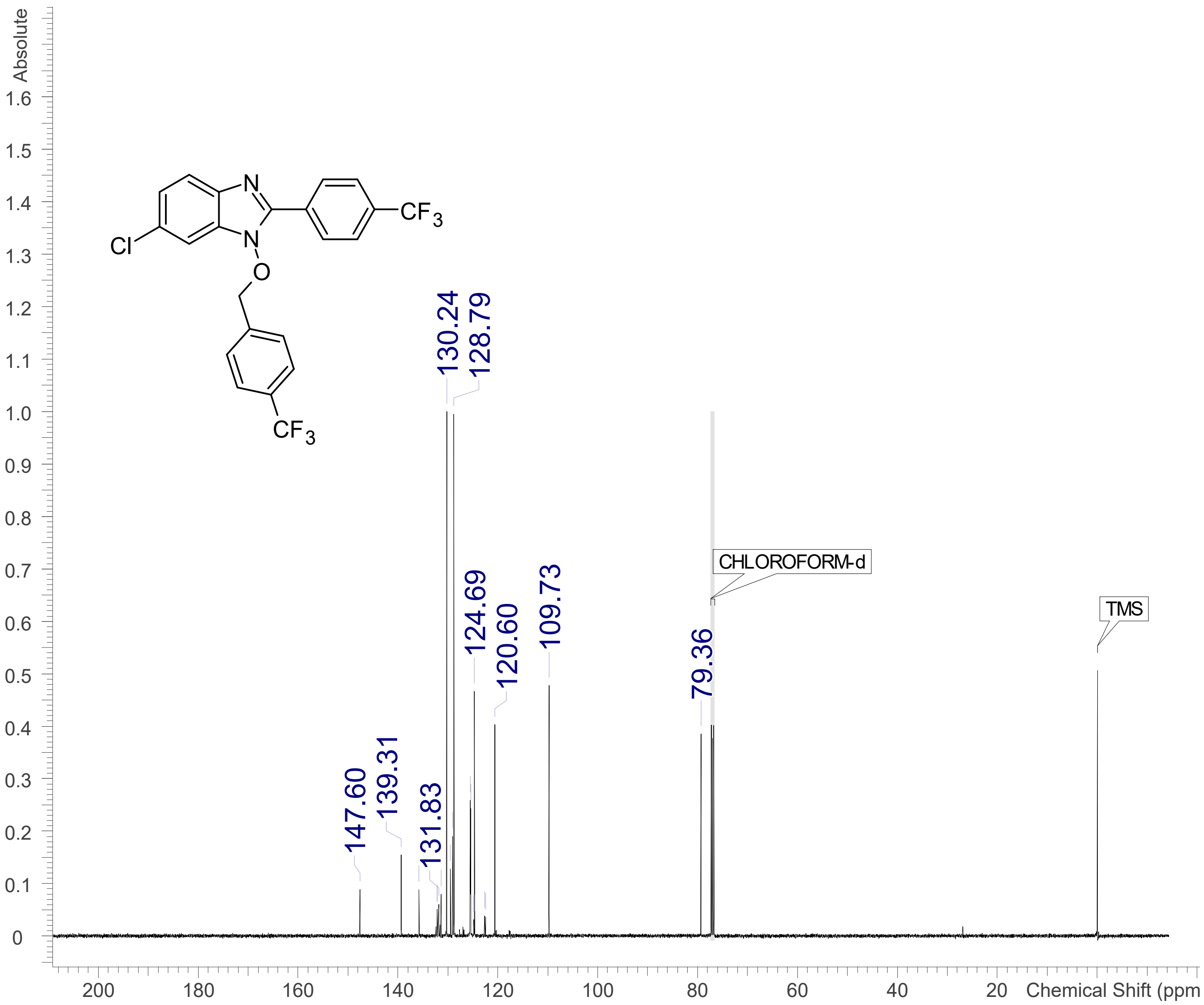

Figure 30S. ^{13}C NMR (126 MHz, CHLOROFORM-*d*) for 6-Chloro-1-((4-(trifluoromethyl)benzyl)oxy)-2-(4-(trifluoromethyl)phenyl)-1*H*-benzo[*d*]imidazole **(11).**

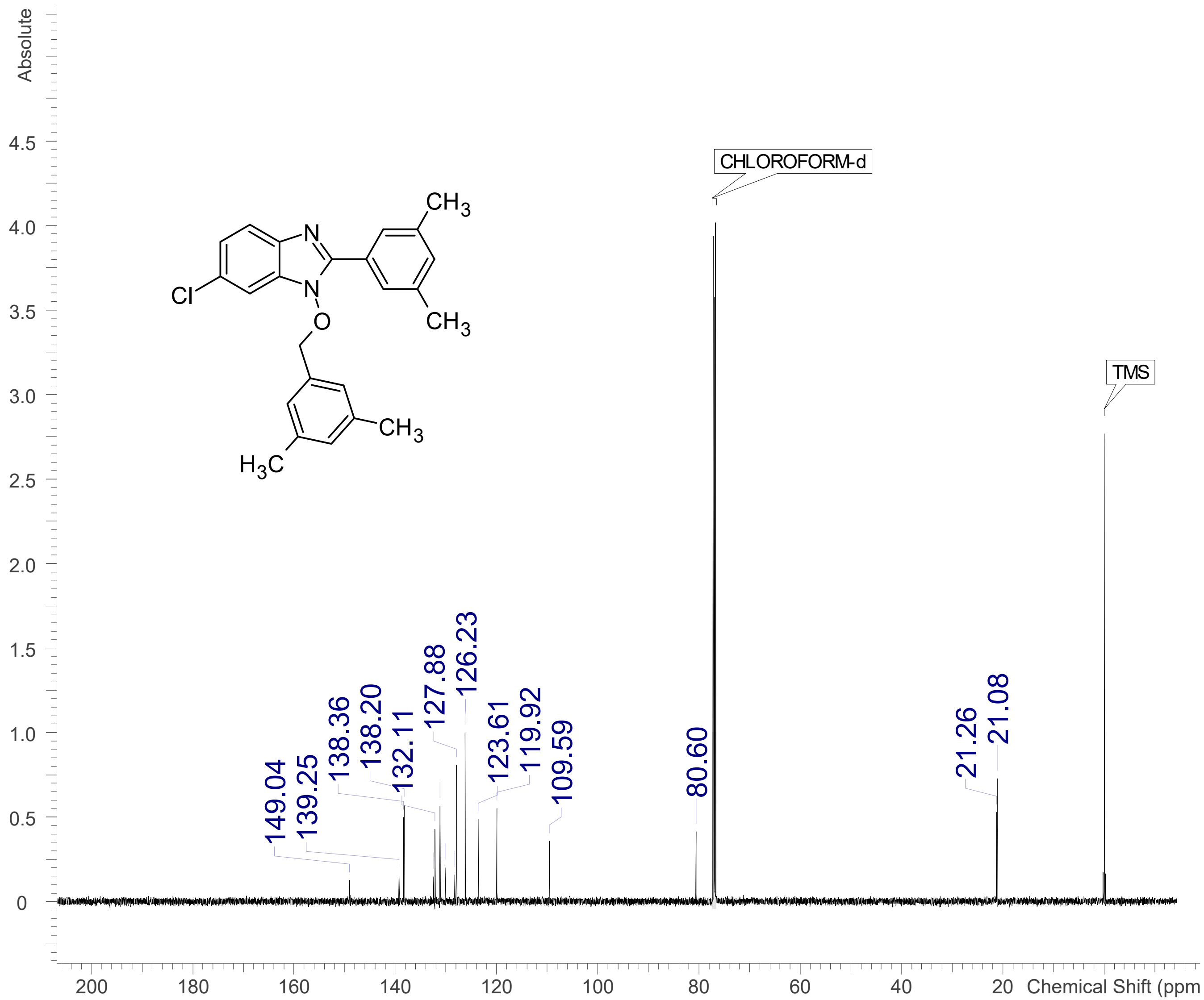

Figure 31S. ^{13}C NMR (126 MHz, CHLOROFORM-*d*) for 6-Chloro-1-((3,5-dimethylbenzyl)oxy)-2-(3,5-dimethylphenyl)-1*H*-benzo[*d*]imidazole **(12).**

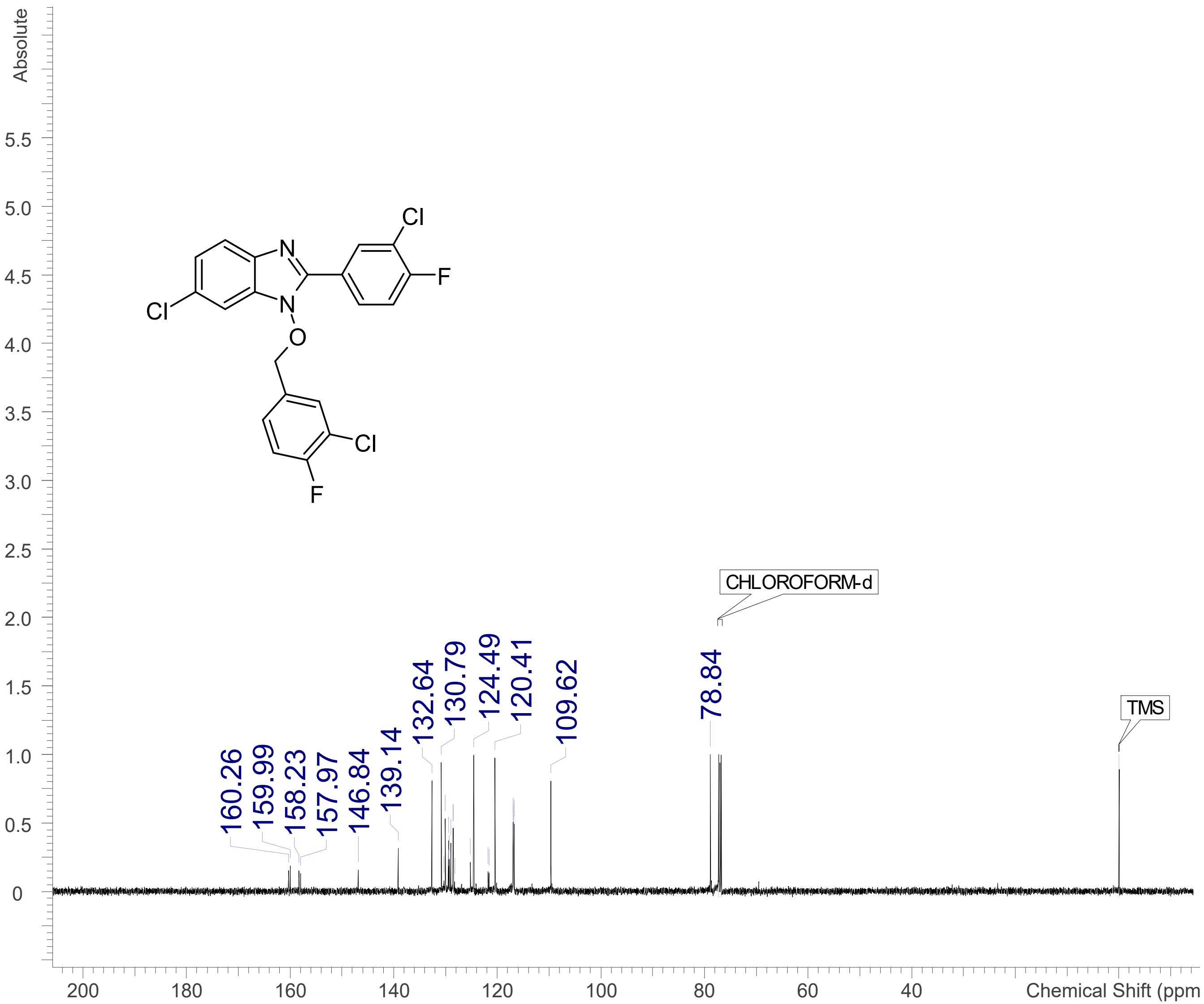

Figure 32S. ^{13}C NMR (126 MHz, CHLOROFORM-*d*) for 6-Chloro-1-((3-chloro-4-fluorobenzyl)oxy)-2-(3-chloro-4-fluorophenyl)-1*H*-benzo[*d*]imidazole **(13).**

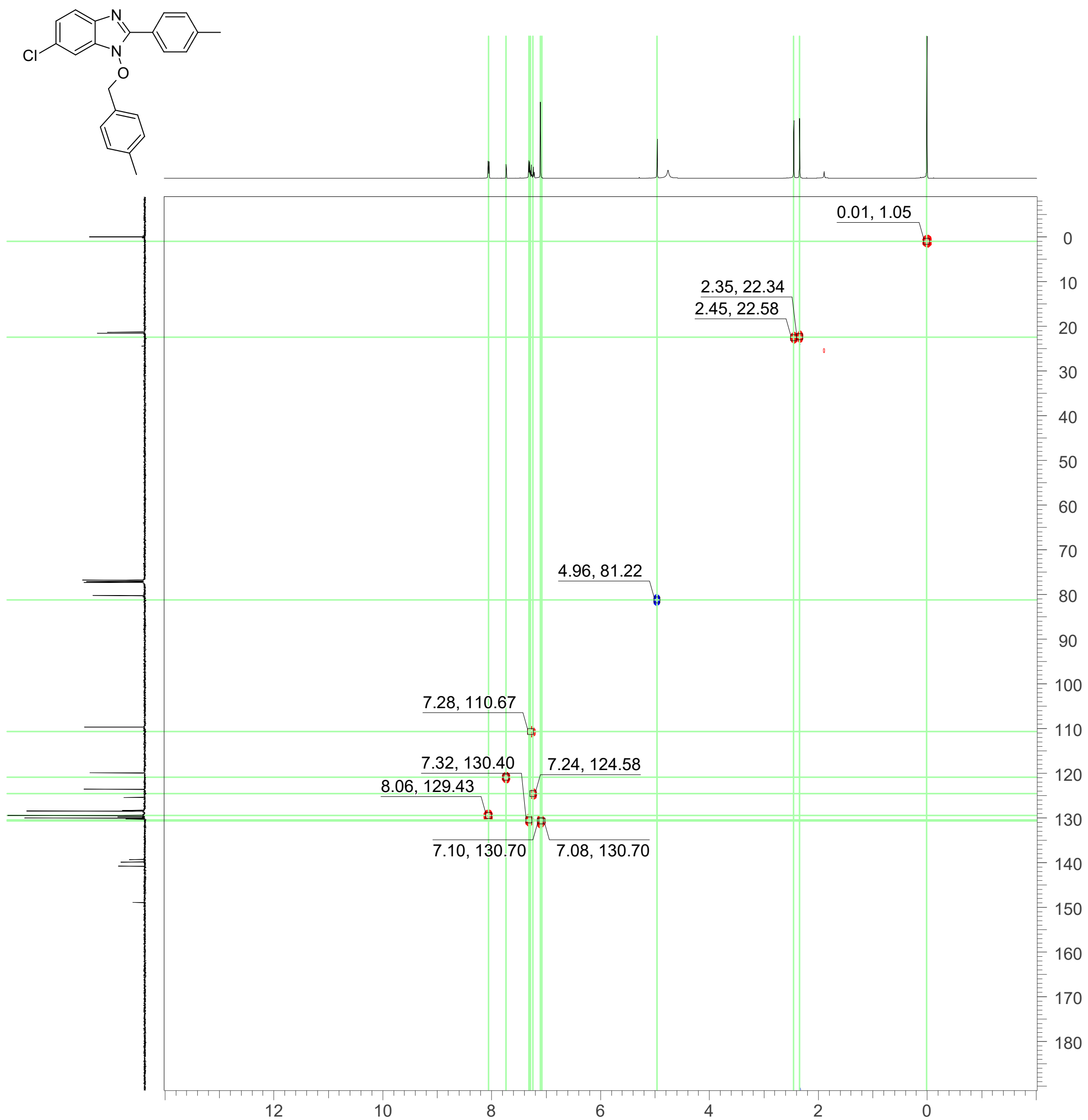

Figure 33S. 2D ^{1}H–^{13}C HSQC NMR spectrum of 6-chloro-1-((4-methylbenzyl)oxy)-2-(*p*-tolyl)-1*H* benzo[*d*]imidazole (**10**), showing the correlations between directly bonded proton and carbon atoms.

Determination of lipophilicity by experimental method

The lipophilicity of compounds **1-13** was evaluated using both computational and experimental approaches. The relative lipophilicity of compounds **1–13** was experimentally evaluated by reversed-phase thin-layer chromatography (RP-TLC) using 20 × 20 cm plates coated with C18 silica RP-18 F254 (Merck, Darmstadt, Germany). Chromatographic separation was performed with a binary acetone–water (2:1, v/v) mobile phase. Stock solutions of the investigated compounds were prepared in ethyl acetate at a concentration of 2.0 mg/mL, and 0.2 µL aliquots were spotted onto chromatographic plates previously activated by drying at 105 °C for 1 h. Development was carried out in ascending TLC chambers at 25 °C until the solvent front reached a distance of 10 cm from the application line. After development, the chromatograms were visualized under UV light at 254 nm. Each compound was analyzed in triplicate by applying the solutions at different positions on the plate. R_F values were determined from the developed chromatograms and reported as the mean of three independent measurements. The relative lipophilicity parameter (R_M) was calculated from the experimental R_F values using the following equation:

$$R_M = \log(1/ R_F - 1)$$

Higher RM values reflect increased lipophilicity.

Computational prediction of ADME properties an drug-likeness

The physicochemical, pharmacokinetic and drug-likeness properties of compounds **1-13** were predicted using SwissADME web server. Computational lipophilicity was assesed by using SwissADME web tool (https://doi.org/10.1038/srep42717). The consesus LogP values was calculated as arithmetic mean of five different lipophilicity prediction models (iLogP, XLOP3, WLOGP, MLOGP and SILICOS-IT) providing a robust estimate of compound lipophilicity, All compounds were predicted to have high gastrointestinal (GI) absorption, with the exception of compounds **11** and **13**. According to the SwissADME analysis, all the investigated compounds (**1–13**) presented a single Lipinski's rule violation, corresponding to an MLOGP value higher than 4.15. No PAINS alerts were detected for compounds **1–13** according to the SwissADME PAINS filter.

Table 1S. Structural and physicochemical descriptors of compounds **1-13**, including SMILES notation, experimentally determined chromatographic retention parameter (R_M), and selected SwissADME descriptors, including consensus LogP values (cLogP), gastrointestinal absorption, PAINS alert, and Lipinski rule violations.

**Cmpd. name**	**R_1**	**R_2**	**SMILES**	**cLogP**	**R_M (exp)**	**GI abs.**	**Lipinski rule**
**1**	H	H	c1ccccc1COn1c(nc2ccccc12)c1ccccc1	4.11	0.308	High	1 violation, MLOGP>4.15
**2**	5-F	H	Fc1cc2nc(n(OCc3ccccc3)c2cc1)c1ccccc1	4.41	0.256	High	1 violation, MLOGP>4.15
**3**	6-F	H	Fc1cc2n(OCc3ccccc3)c(nc2cc1)c1ccccc1	4.41	0.447	High	1 violation, MLOGP>4.15
**4**	5-Cl	H	Clc1cc2nc(n(OCc3ccccc3)c2cc1)c1ccccc1	4.65	0.219	High	1 violation, MLOGP>4.15
**5**	6-Cl	H	Clc1cc2n(OCc3ccccc3)c(nc2cc1)c1ccccc1	4.65	0.403	High	1 violation, MLOGP>4.15
**6**	6-Cl	3-Cl	Clc1ccc2nc(n(OCc3cc(Cl)ccc3)c2c1)c1cccc(Cl)c1	5.84	0.659	High	1 violation, MLOGP>4.15
**7**	6-Cl	3-CH_3	Clc1ccc2nc(n(OCc3cccc(C)c3)c2c1)c1cccc(C)c1	5.35	0.584	High	1 violation, MLOGP>4.15
**8**	6-Cl	4-F	Fc1ccc(cc1)c1nc2ccc(Cl)cc2n1OCc1ccc(F)cc1	5.44	0.382	High	1 violation, MLOGP>4.15
**9**	6-Cl	4-Cl	Clc1ccc2nc(n(OCc3ccc(Cl)cc3)c2c1)c1ccc(Cl)cc1	5.84	0.668	High	1 violation, MLOGP>4.15
**10**	6-Cl	4-CH_3	Clc1cc2n(OCc3ccc(C)cc3)c(nc2cc1)c1ccc(C)cc1	5.35	0.611	High	1 violation, MLOGP>4.15
**11**	6-Cl	4-CF_3	FC(F)(F)c1ccc(cc1)c1nc2ccc(Cl)cc2n1OCc1ccc(cc1)C(F)(F)F	7.10	0.668	Low	1 violation, MLOGP>4.15
**12**	6-Cl	3,5-$(CH_3)_2$	Cc1cc(C)cc(c1)c1nc2ccc(Cl)cc2n1OCc1cc(C)cc(C)c1	6.12	0.865	High	1 violation, MLOGP>4.15
**13**	6-Cl	3-Cl, 4-F	Fc1ccc(cc1Cl)c1nc2ccc(Cl)cc2n1OCc1cc(Cl)c(F)cc1	6.63	0.639	Low	1 violation, MLOGP>4.15

Figure 34S. Ramachandran plot of the α-glucosidase model predicted by AlphaFold3 generated using PROCHECK.

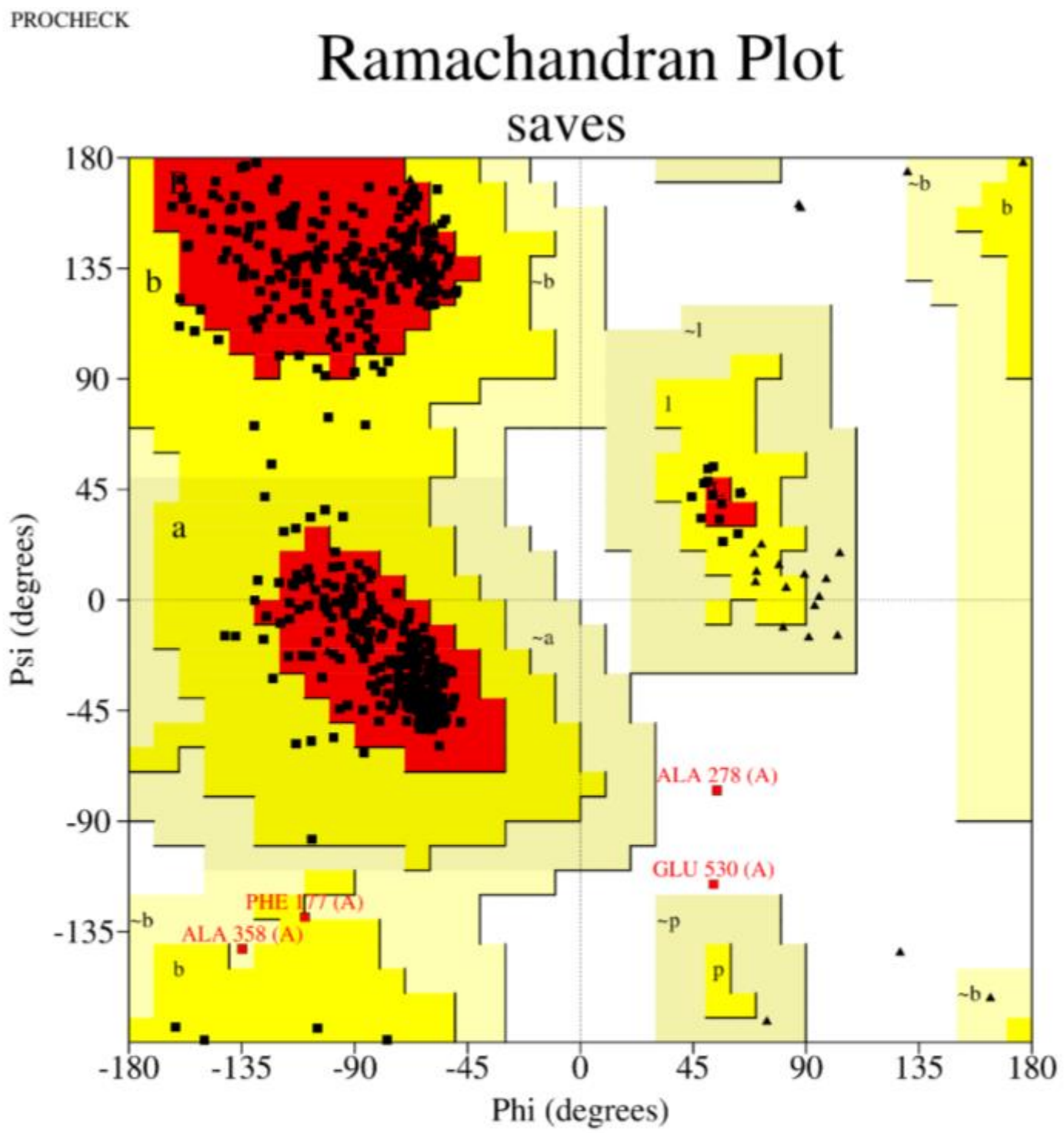

Plot statistics

Residues in most favoured regions [A,B,L]	466	90.1%
Residues in additional allowed regions [a,b,l,p]	47	9.1%
Residues in generously allowed regions [~a,~b,~l,~p]	2	0.4%
Residues in disallowed regions	2	0.4%
	----	------
Number of non-glycine and non-proline residues	517	100.0%
Number of end-residues (excl. Gly and Pro)	2	
Number of glycine residues (shown as triangles)	37	
Number of proline residues	28	

Total number of residues	584	

Based on an analysis of 118 structures of resolution of at least 2.0 Angstroms and R-factor no greater than 20%, a good quality model would be expected to have over 90% in the most favoured regions.

Figure 35S. 2D depiction of the interactions between alpha glucosidase and compounds **6** (A), **13** (B), **1** (C) and **2** (D) predicted by Discovery Studio Visualizer.

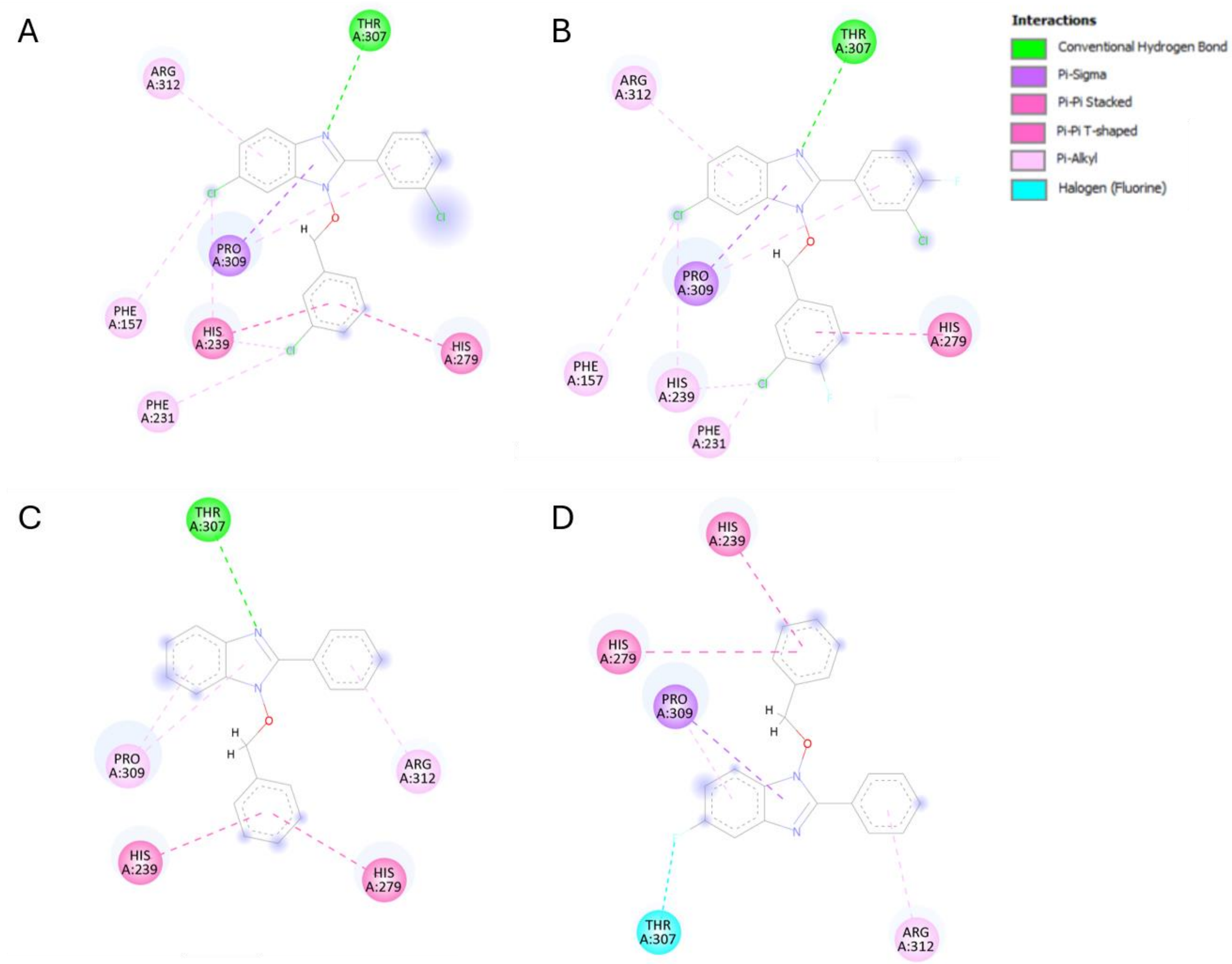

Supplement: Supplementary file 1 [file MD-OLF-D6MD00752J-s001.pdf]
